# Long term evaluation of factors influencing the association of ixodid ticks with birds in Central Europe, Hungary

**DOI:** 10.1038/s41598-024-55021-9

**Published:** 2024-02-29

**Authors:** Gergő Keve, Tibor Csörgő, Dávid Kováts, Sándor Hornok

**Affiliations:** 1 Department of Parasitology and Zoology, University of Veterinary Medicine, Budapest, Hungary; 2 HUN-REN-UVMB Climate Change: New Blood-Sucking Parasites and Vector-Borne Pathogens Research Group, Budapest, Hungary; 3 Department of Anatomy, Cell- and Developmental Biology, Eötvös Loránd University, Budapest, Hungary; 4 Ócsa Bird Ringing Station, Ócsa, Hungary; 5 BirdLife Hungary, Budapest, Hungary; 6 Hungarian Biodiversity Research Society, Budapest, Hungary

**Keywords:** Ecology, Zoology

## Abstract

Birds play a crucial role in disseminating ticks that carry pathogens of high veterinary-medical importance. The aim of this study was to analyze data of a long-term tick collection from birds at a single stop-over site in Central Europe, Hungary. Over eight years (2015–2022) 5833 ticks (ten species) were collected from 2395 tick-infested birds. The most abundant species were *Ixodes ricinus* (n = 3971) and *Haemaphysalis concinna* (n = 1706). *Ixodes ricinus* nymphs and larvae were the most frequently occurring on resident and short-distance migratory birds with forest habitat but *Ha. concinna* was the most abundant species on reed-associated, long-distance migrants. *Haemaphysalis concinna* occurred mostly on birds feeding above the ground level, while *I. ricinus* predominated on ground feeding birds. Infestation with *I. ricinus* nymphs always peaked in the first half of the year, in contrast to larvae which were more abundant on avian hosts in the autumn. At the same time, *Ha. concinna* larvae and nymphs had their peak numbers in the summer. This is the first long-term study on the tick infestation of birds in Central Europe. The study shows that, migration distance, habitat type, and typical feeding level of birds, as well as characteristics of tick life cycle are all key factors in the role of birds as tick disseminators. It was revealed that Savi’s Warbler (*Locustella luscinioides*) is the most frequent hosts of *Ha. concinna* in Central Hungary.

## Introduction

Hard ticks (Acari: Ixodidae) are common carriers of pathogens that can affect both humans and animals, so it is not surprising that they are considered one of the world’s most important arthropod vectors^1^. Because of this, research on their ecology and distribution is of utmost importance^2^. This is especially true nowadays, as ecological systems are transforming rapidly due to climate change^3^.

The role of birds in the dissemination of ticks is unquestionable. Migratory birds can transport ticks between continents, for instance from Africa into Europe^4^. Thermophilic tick species, most notably from the genus *Hyalomma*, have long been considered adventitious, but in recent years these species have been reported in Europe with increasing numbers north of the Mediterranean Basin, and were even able to establish a resident population^5–8^. In this context, the importance of synanthropic, resident bird species (e.g. the Blackbird (*Turdus merula*)) is also crucial, regarding the local dissemination of ticks, as they can introduce ticks to urban areas^9^. The monitoring of bird-tick relationships is a long-standing and intensively researched field that has greatly contributed to what is known about ticks and the epidemiology of the pathogens they transmit^10–12^.

Research on this topic has been conducted in Hungary for decades, particularly at Ócsa Bird Ringing Station^8,13–17^. At this station more than 15 thousand birds are caught yearly. The area has several different habitat types (e.g. forest, arable field, reedbed)^18^ and is an important stop-over site for birds migrating along the Adriatic Flyway through Central Europe. Therefore, it is suitable for the examination of both migratory and resident birds of different habitats.

In order to evaluate factors that influence the association of ixodid ticks with avian hosts, ticks were collected from birds at Ócsa Bird Ringing Station between March 2015 and November 2022. The data collected over the course of eight years ensure a better understanding of ecological and ornithological factors that influence the epidemiological significance of birds as tick carriers.

## Results

### Identification of tick species and their occurrence on infested birds

During the study period 2395 tick-infested birds were captured, belonging to 51 different species, and a total of 5833 ticks were collected. The most frequent host species were the European Robin (*Erithacus rubecula*) (n_infested_ = 521, n_captured_ = 14,809), followed by the Blackbird (*T. merula*) (n_infested_ = 359, n_captured_ = 1525). The mean number of ticks found on infested birds (i.e., intensity of infestation) was 2.44, while the median intensity was 1.

Based on morphological characteristics, eight tick species were identified, namely *Ixodes ricinus, Ixodes frontalis, Ixodes lividus, Ixodes festai, Ixodes arboricola, Haemaphysalis concinna, Haemaphysalis punctata* and* Dermacentor reticulatus* (Table 1). Concerning molecular identification of *Hyalomma* species, one nymph showed 100% identity to sequences of *Hyalomma rufipes* reported in Africa and in Malta (cox1 gene: OQ540949 from Kenya; 12S rRNA gene: OL352890 from Malta, 16S rRNA gene: MK737649 from Egypt). Two further nymphs proved to be *Hyalomma marginatum*, one of them with 100% sequence identities to ticks reported from the eastern-middle-western Mediterranean region (cox1 gene: LC508365 from Portugal; 12S rRNA gene: OL352894 from Malta; 16S rRNA gene: KT391060 from Israel) and another with 99.8–100% sequence identities to specimens reported from the western Mediterranean region (cox1 gene: LC508365 from Portugal; 16S rRNA gene: LC508322 from Portugal).

**Table 1:** Total number of tick species and stages found during the study (2015–2022).

Tick species	Larva	Nymph	Female	Male	Total
*Ixodes ricinus*	1229	2742	0	0	3971
*Ixodes frontalis*	52	38	12	0	102
*Ixodes lividus*	0	1	12	0	13
*Ixodes festai*	0	0	6	2	8
*Ixodes arboricola*	0	1	0	0	1
*Haemaphysalis concinna*	698	1008	0	0	1706
*Haemaphysalis punctata*	28	0	0	0	28
*Dermacentor reticulatus*	0	0	1	0	1
*Hyalomma marginatum*	0	2	0	0	2
*Hyalomma* *rufipes*	0	1	0	0	1

Altogether, 1872 (78.16%) of the captured, tick-infested birds carried *I. ricinus*, while 592 (24.72%) of them carried *Ha. concinna*. *Ixodes frontalis* was present on 51 birds (2.13%). The remaining seven tick species were carried by a combined total of 13 (0.54%) birds. One hundred thirty-one birds carried two tick species, and on one Song Thrush (*Turdus philomelos*) three different tick species were present simultaneously (*I. ricinus*, *I. frontalis* and *Ha. concinna*).

The 2395 tick-carrying birds captured during this study are summarized in Table 2, according to the characteristics that were examined. In the table, we also indicated how many bird species belonged to the given category. More detailed information can be found in Supplementary Tables 2 and 3.

**Table 2:** Total number of birds, and bird species found to be tick-infested between 2015 and 2022, according to their migration habit, habitat and feeding place.

Migration habit		Habitat		Feeding place	
R	78 (5 species)	Reed	621 (11 species)	Above ground	463 (23 species)
SDM	536 (12 species)	Forest	1356 (30 species)	Ground	1877 (23 species)
MDM	4 (1 species)	Meadow	47 (7 species)	Ground/Above ground	51 (4 species)
R/SDM	912 (10 species)	Forest/Meadow	367 (2 species)	Only flying insects	4 (1 species)
R/MDM	32 (2 species)	Sand walls	4 (1 species)		
LDM	833 (21 species)				

R: resident, SDM: short-distance migrants, MDM: middle-distance migrants, R/SDM: resident or short-distance migrants, R/MDM: residents or middle-distance migrants, LDM: long-distance migrants.

”Sand walls” and ”Only flying insects” are categories in which only the Sand Martin (*Riparia riparia*) belongs. On this bird, only *I. lividus* has been found.

More information can be found in Supplementary Tables 2 and 3.

### Number and temporal occurrence of tick species, their host associations according to habitat and migration characteristics

**Figure 1:**
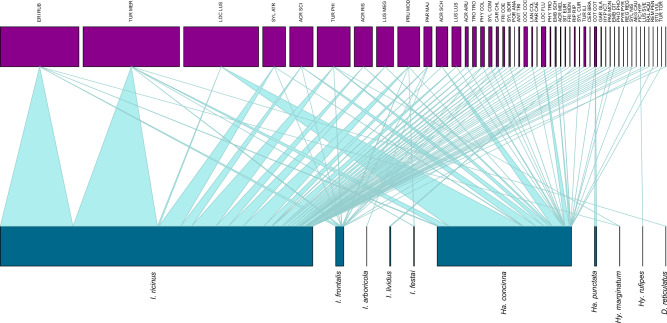
Tick-host associations visualized on a plotweb.

All tick-host associations are listed in Supplementary Tables 1 and 2 and visualized in Fig. 1 and Supplementary Fig. 1 Statistical analyses on ticks and their hosts according to their habitat and migration characteristics were only calculated for the most abundant tick species (*I. ricinus* and *Ha. concinna*), due to the fact that the limited numbers of other tick species precluded robust statistical analyses.

***Ixodes ricinus*** (n_total_ = 3971, n_larvae_ = 1229, n_nymphs_ = 2742)**:** *Ixodes ricinus* subadults occurred most frequently on resident and/or short-distance migrating birds with forest or forest and meadow habitats (Tables 3, 4), This category includes the two species on which the most ticks were found: Blackbirds (n_ticks_ = 1073, n_birds_ = 326), and European Robins (n_ticks_ = 924, n_birds_ = 490) (Table 5). *Ixodes ricinus* ticks were present on birds year-round, with nymphs reaching their peak in the first half of the sampling periods (1st of March–30th of June), while larvae were the most abundant in the second half (1st of July–31 of October). This was consistent over the course of eight years. The difference between the numbers of nymphs and larvae regarding their half-yearly activity was significant (p < 0.0001) (Supplementary Table 1, Supplementary Fig. 2).

**Table 3:** Number of ticks according to the typical habitat of their avian hosts.

Tick species and stage	Meadow	Forest	Meadow/forest	Reed
*Ixodes ricinus* L	22	981	105	121
*Ixodes ricinus* N	41	1482	981	238
*Haemaphysalis concinna* L	3	107	45	543
*Haemaphysalis concinna* N	6	128	84	790
*Ixodes frontalis* L	0	39	13	0
*Ixodes frontalis* N	0	32	4	2
*Ixodes frontalis* F	1	9	1	1
*Ixodes festai* F + M*^1^	0	3	5	0
*Hyalomma marginatum* N	0	2	0	0
*Hyalomma rufipes* N	0	1	0	0

L: larva, N: nymph, F: female, M: male.

*^1^: we treated males and females together, as the two males we found were in copulation with females.

**Table 4:** Number of ticks according to the migration habit of their avian hosts .

Tick species and stage	R	SDM	MDM	R/SDM	R/MDM	LDM
*Ixodes ricinus* L	16	278	1	586	3	345
*Ixodes ricinus* N	96	700	4	1450	70	422
*Haemaphysalis concinna* L	0	32	0	61	0	605
*Haemaphysalis concinna* N	1	95	0	109	2	801
*Ixodes frontalis* L	0	10	0	42	0	0
*Ixodes frontalis* N	2	7	0	26	1	2
*Ixodes frontalis* F	5	3	0	3	1	0
*Ixodes festai* F + M*^1^	0	3	0	5	0	0
*Hyalomma marginatum* N	0	1	0	1	0	0
*Hyalomma rufipes* N	0	0	0	0	0	1

L: larva, N: nymph, F: female, M: male.

R: resident, SDM: short-distance migrants, MDM: middle-distance migrants, R/SDM: resident or short-distance migrants, R/MDM: residents ot middle-distance migrants, LDM: long-distance migrants.

*^1^: we treated males and females together, as the two males we found were in copulation with females.

**Table 5:** The five most common avian hosts of *Ixodes ricinus* (A) and *Ha. concinna* (B).

(A)				
Bird species	Number of tick-infested birds during our study	Total number of ticks	Number of *I. ricinus*	Medians of total *I. ricinus *larva/nymph infestations
TUR MER	359	1226	1073	1/2
ERI RUB	521	1000	924	1/1
TUR PHI	168	419	310	2/1
SYL ATR	199	290	277	1/1
PRU MOD	90	279	262	1/2

(B)				
Bird species	Number of tick-infested birds during our study	Total number of ticks	Number of *Ha. concinna*	Medians of total *Ha. Concinna *larva/nymph infestations
LOC LUS	243	952	933	2/2
ACR SCI	167	297	157	1/1
ACR SCH	54	148	135	2.5/1
TUR MER	359	1226	129	1/1
TUR PHI	168	419	102	1/1

***Ixodes frontalis*** (n_total_ = 102, n_larvae_ = 52, n_nymphs_ = 38, n_female_ = 12)**:** The European Robin was the most common host of this tick species (n_ticks_ = 52, n_birds_ = 21). The second most frequently identified host species was the Blackbird (n_ticks_ = 18, n_birds_ = 4). *Ixodes frontalis* occurred most frequently on resident and/or short-distance migrating birds with forest habitat (Tables 3, 4)**.** All stages of *I. frontalis* were the most abundant in the second half of March every year, during the eight-year-long study period (Supplementary Fig. 3).

***Ixodes lividus*** (n_total_ = 13, n_females_ = 12, n_nymphs_ = 1)***:*** All 13 *I. lividus* ticks were collected from four Sand Martins (*Riparia riparia*).

***Ixodes festai*** (n_total_ = 8, n_females_ = 6, n_males_ = 2)**:** In total eight ticks of this species were collected. Five ticks were removed from two Blackbirds and three ticks from two Dunnocks (*Prunella modularis*). It is important to mention, that the two males collected were not feeding, but were in copulation with females.

***Ixodes arboricola*** (n_total_ = 1, n_nymphs_ = 1): Only one nymph was collected. It was removed from a Great Tit (*Parus major*).

***Haemaphysalis concinna*** (n_total_ = 1706, n_larvae_ = 698, n_nymphs_ = 1008): The majority (933 ticks, 54.7%) of individuals of this tick species were found on Savi’s Warbler (*Locustella luscinioides*) (n_birds_ = 238), making this bird the most common host of *Ha. concinna*. This tick species was also frequently collected from Eurasian Reed Warbler (*Acrocephalus scirpaceus*) (n_ticks_ = 157, n_birds_ = 82). The five most common hosts of *Ha. concinna* are listed in Table 5. Both *Ha. concinna* larvae and nymphs were active in the summer. However, the peaks of their abundance showed differences over the study period. In particular, the peak of infestation of birds with *Ha. concinna* larvae preceded that of its nymphs in 2015 and 2022, but the opposite trend was observed in 2017 and 2018. In other years, the peak larval and nymphal infestations occurred simultaneously (Supplementary Fig. 2).

It was found that the host habitat (reed, forest, meadow, meadow/forest) of *I. ricinus* and *Ha. concinna* was significantly different (p < 0.0001) (Table 3). We also compared the migratory habits (resident, short-distance migrants, middle-distance migrants, resident or short distance migrants, resident or middle-distance migrants, long-distance migrants) of the hosts of *I. ricinus* and *Ha. concinna*. The difference was strongly significant (p < 0.0001) (Table 4). According to our findings, *Ha. concinna* occurred most frequently on long-distance migrant and reed-associated birds, while *I. ricinus* was the most abundant on short-distance migrants, with forest habitat. (Tables 3, 4).

***Haemaphysalis punctata*** (n_total_ = 28, n_larvae_ = 28)**:** A single Common Quail (*Coturnix coturnix*) was found with 28 feeding larvae.

***Hyalomma marginatum*** (n_total_ = 2, n_nymphs_ = 2): One engorged nymph was collected from European Robin, and another from Song Trush (*T. philomelos*). Both were collected in the first half of April (in 2015, and 2016, respectively).

***Hyalomma rufipes*** (n_total_ = 1, n_nymph_ = 1): Only one engorged nymph was found, which was feeding on a European Pied Flycatcher (*Ficedula hypoleuca*) in the second half of April 2015.

***Dermacentor reticulatus*** (n_total_ = 1, n_female_ = 1): One female of this species was found on a Blackbird (*Turdus merula*), though it had not started to feed.

### Host associations of ticks according to bird weight and feeding level characteristics

The mean intensity of infestation with *I. ricinus* nymphs was the highest among bird species with typical body weight above 100 g, whereas this was the lowest among bird species measuring below 10 g (Supplementary Fig. 4). The same was not true in the case of *Ha. concinna* nymphs, because the mean intensity of tick-infestation was the highest on birds weighing between 10.1 and 20 g (Supplementary Fig. 4).

When the numbers of *I. ricinus*, *I. frontalis* and *Ha. concinna*  were compared according to the feeding level of their hosts (only ground level or above ground categories were tested), *I. ricinus* tended to predominate on ground-feeding birds, whereas *Ha. concinna* nymphs and larvae were more often found on birds looking for food items above the ground level (p < 0.0001). On the other hand, the difference was not significant between *I. ricinus* and *I. frontalis* (p = 0.2584) (Table 6).

**Table 6:** Number of ticks according to the typical feeding place of their avian hosts.

Tick species and stage	Ground	Above ground	Ground/above ground
*Ixodes ricinus* L	896	328	5
*Ixodes ricinus* N	1836	890	16
*Haemaphysalis concinna* L	144	554	0
*Haemaphysalis concinna* N	204	804	0
*Ixodes frontalis* L	42	10	0
*Ixodes frontalis* N	31	7	0
*Ixodes frontalis* F	5	7	0
*Ixodes festai* F + M*^1^	5	3	0
*Hyalomma marginatum* N	2	0	0
*Hyalomma rufipes* N	0	1	0

L: larva, N: nymph, F: female, M: male.

*^1^: we treated males and females together, as the two males we found were in copulation with females.

## Discussion

Although there are many similar previous reports from different countries (e.g.^19–24^), this study presents one of the longest continuous bird tick collections in Europe. Over an eight-year-long period, 5833 ticks of 10 species were collected from 2395 birds of 51 species. The dataset itself provides a valuable contribution to the field due to its size.

The two most abundant tick species collected were *I. ricinus* (n_total_ = 3971) and *Ha. concinna* (n_total_ = 1706). Only subadult stages were found in case of both species. *Ixodes ricinus* has long been known as the most common tick species that feeds on birds in the Palearctic region^12^ and is primarily a forests-dwelling tick species^25^. This fact was confirmed by data presented here, as *I. ricinus* occurred most frequently on birds with forest habitat. Interestingly, this was not true for *Ha. concinna*. According to the literature data, *Ha. concinna* can be found in a broad range of different habitats, mainly moist, wooded ecosystems, but in reedbeds as well^25–27^. However, according to our data, this species mainly parasitized reed-associated birds (1333 *Ha. concinna* ticks on birds with reed habitat). The difference between the host habitats of *I. ricinus* and *Ha. concinna* was strongly significant (p < 0.0001). Furthermore, 933 *Ha. concinna* ticks were collected from only one bird species, Savi’s Warbler. According to Csörgő et al.^28^ number of ringed Savi’s Warblers present in Hungary between 1951 and 2006 was 32 083 birds. Interestingly the same numbers of the second and third most common hosts from Ócsa (Eurasian Reed Warbler and Sedge Warbler (*Acrocephalus schoenobaenus*)) were over 220 000 for each species. Savi’s Warbler had the highest level of median infestation in the case of nymphs, and the second highest in the case of larvae. In our opinion however, larvae are not the greatest indicators in this regard, as a low presence of larvae can be overlooked easily due to the small size of the parasites.

Regarding the migration habit of the hosts of *Ha. concinna*, and *I. ricinus*, the results were in line with our previous observation^8^ on a strongly significant difference between the hosts of the two tick species: *Ha. concinna* most commonly occurred on long-distance migrants, whereas *I. ricinus* was most frequently collected from short-distance migrant or resident birds. *Haemaphysalis concinna* is a thermophilic tick species. Its larvae and nymphs have a similar period of peak activity (mainly the summer)^27^ and its typical avian hosts are reed-associated long-distance migrants^28^.

Among the latter, Savi’s Warbler was the most common host in Hungary. According to our hypothesis, the feeding habit of this bird species may explain this phenomenon. Savi’s Warbler shares a very similar ecological niche with *Ha. concinna*, because it mainly feeds on small invertebrates near the water surface in reedbeds. This behavior is consistent during the migration period^28^, and lakeshore vegetation was reported to be a preferred habitat type of *Ha. concinna* in Central Europe^27^.

Taken together, *Ha. concinna* subadults apparently share a much more similar ecological niche with Savi’s Warbler, than with other reed-associated songbirds. It is important to mention that this difference is only true among avian hosts and does not extend to mammals and reptiles, which are also favored hosts of *Ha. concinna* ^27^, especially roe deer^29^.

It was previously reported, that birds with larger body mass carry a higher number of *I. ricinus* nymphs^30^. In this study, the mean intensities of infestations with *I. ricinus* and *Ha. concinna* stages were categorized according to the average body mass of their avian hosts. In the case of *I. ricinus*, the mean intensity of nymphs showed a trend of increase with the average body mass of the hosts, unlike in the case of *Ha. concinna* nymphs. However, this could not be supported by the results of statistical analyses, because of the following reasons. First, the majority of *Ha. concinna* ticks were carried by Savi’s Warblers, which is a small size bird species. Second, *I. ricinus* occurred most frequently on ground feeding birds which in general have a larger body mass (e.g., Blackbirds). On the other hand, data on the body mass of bird individuals examined for tick-infestation in the present study were not available, and this parameter is known to change significantly even within the same bird species between different seasons.

The temporal distribution of *I. ricinus* and *Ha. concinna* developmental stages was also analyzed. *Ixodes ricinus* nymphs always reached their peak infestation on birds during the first half of the annual sampling periods throughout the study, whereas larvae reached their highest abundance from July to October. This is likely a consequence of the prolonged development of *I. ricinus* which takes several years in Central Europe^31^ implying that nymphs and larvae collected in the same year belonged to different generations. By contrast, the nymphal and larval peak activities of *Ha. concinna* were always close to each other, i.e., within 0.5–1.5 month over the summer period. This may reflect that a notable portion of these developmental stages belonged to the same generation, particularly when the larval peak preceded the nymphal peak (e.g., in 2015, 2022). This is in line with observations that under temperate climate this tick species can complete one generation in one year if hosts are available^32^ On the other hand, *Ha. concinna* larvae were also found on birds as early as March and April (e.g., in 2016 and 2017), suggesting overwintering in the larval stage and a generation time longer than one year^27^.

During the eight-year-long study period, 102 *I. frontalis* ticks were collected (n_larvae_ = 52, n_nymphs_ = 38, n_female_ = 12). According to the known literature data, *I. frontalis* reaches its peak in March and in November^33,34^. In this study, the vast majority of all developmental stages were collected in the second half of March. The absence of a peak in November is likely because the main sample collection period was finished at the end of October/beginning of November each year. From November tick collections, there are only a scarce and random amount of data. The fact that this tick is the most abundant at the beginning of spring and the end of autumn explains why *I. frontalis* showed association with resident and/or short-distance migrant birds according to our data (Table 4). *Ixodes frontalis* was almost exclusively found on birds with forest- or mixed meadow/forest habitats (98/102) (Table 3).

Altogether 8 specimens of *I. festai*, six females and two males were also collected during the study period. All hosts were Blackbirds and Dunnocks. The same host species were found to be infested in a previous study from another part of Central Europe, Switzerland ^35^, but literature data are available from multiple other host species as well ^12^.

Three *Hyalomma* nymphs were found in total. According to our molecular analyses, two were *Hy. marginatum* and one was *Hy. rufipes*. Each of these nymphs had 100% sequence identity with at least one specimen reported from the mid-Mediterranean region (Malta), situated along the Adriatic Flyway crossing Hungary toward the north. Thus, these data are highly relevant to their probable geographical origin. *Hyalomma* species are important vectors of sevedunral different pathogens, including the Chrimean-Congo haemorrhagic fever virus^25^. All *Hyalomma ticks* were collected in April (2015, 2016). Findings of *Hyalomma* subadults during the spring migration period are not uncommon in Central Europe^36^ and in Ócsa^16^. It is important to note, however, that the monitoring of *Hyalomma-*carrying birds is of great epidemiological importance.

Thirteen *I. lividus* were identified. This tick is the host-specific parasite of Sand Martin*,* that feeds extremely rarely on other birds^12,25,37^. It is not surprising that we have found all *I. lividus* ticks on Sand Martins. *Ixodes lividus* have been reported from Hungary long before our study^38^.

Twenty-eight *Ha. punctata* larvae were found feeding on a single Common quail. *Haemaphysalis punctata* has been already known in Hungary, as an uncommon parasite on birds ^39^.

Only one *I. arboricola* nymph was collected during this study. This low number is not surprising, since *I. arboricola* is a nidicolous tick that feeds on nestlings during the summer, and therefore adult birds are mostly infested during winter seasons, when roosting ^40^. For this study, sample collection period started in March and ended at the beginning of November each year so did not include the peak season for *I. arboricola*.

Lastly, one *D. reticulatus* female on a Blackbird was found in 2022. This tick was not (yet) feeding, and its occurrence is believed to be accidental, though *D. reticulatus* subadults have been found on Blackbirds (among other birds) before ^41^.

## Conclusions

Based on this 8-year survey, the migration distance, the habitat type, and the feeding habit of birds, as well as the seasonal activity of ticks are all important factors determining the role of birds as tick disseminators. *Haemaphysalis concinna* was the most abundant on long-distance migrant, reed-associated, above-ground feeder birds, in contrast to *I. ricinus*, which predominated on resident or short-distance migrant, ground-feeder birds with forest or meadow habitat. In the study region, Savi’s Wabler (*L. luscinioides*) was by far the most common avian host of *Ha. concinna* larvae and nymphs.

## Methods

### Sample collection

Sample collection took place from March 2015 to November 2022 at Ócsa Bird Ringing Station (N47.2970, E19.2104), approximately 33 km south of Budapest. Ócsa is situated in a humid continental transitional climate zone. Summers are medium warm and dry, with relatively cold winters. The annual average temperature is 10.1 °C (minimum ca. − 15.6 °C, maximum ca. 34.1 °C). Annual precipitation is around 550–580 mm. Annual sunshine is ca. 2000 h. The mean elevation is 100 m above sea level^18^. There are a large variety of different habitats in the area of the station: it is surrounded by arable fields, but forests, shrubs, and garden yards, can also be found here. Due to the fact, that Ócsa is situated on the edge of a wetland, open water surfaces and reedbeds are also common^18^.

Birds were mist-netted for ringing by standard ornithological mist-nets (mesh size 16 mm). During the ringing procedure, birds were examined for the presence of ticks. Ticks were removed with the help of pointed tweezers and were placed and stored in 96% ethanol. Bird ringing and tick collection were constant from the beginning of March to the end of October each year. Only sporadic data were obtained during November and December. During 2020 and 2021, due to COVID-19 restrictions, bird ringing activity was reduced and tick collections were limited. Birds were handled, identified, and released by professional ringers throughout our study. Only data about tick-infested birds were recorded. Some data from 2022 were used for another study in a different context ^8^.

### Morphological identification

Ticks were identified with a stereomicroscope (SMZ-2 T, Nikon Instruments, Japan, illuminated with model 5000-1, Intralux, Switzerland). To identify *Ixodes ricinus, I. frontalis, I. lividus, I. arboricola,* and *Dermacentor reticulatus* to the species level and *Hyalomma* species to the genus level, we used morphological keys provided by Estrada-Peña et al.^25^ *Hyalomma* ticks were identified to the species level with molecular methods^8^. Differentiation of subadults of *Haemaphysalis concinna* and *Ha. punctata* was based on the morphological keys by Filippova^42^**.** For the identification of *I. festai,* we used the manuscripts of Contini et al.^43^ and Hornok et al.^14^.

### DNA extraction and molecular identification of *Hyalomma* species

These steps were carried out as in Keve et al.^8^**.** Purification and sequencing of the PCR products were done by Biomi Ltd. (Gödöllő, Hungary). Quality control and trimming of sequences were performed with the BioEdit program, then alignment with GenBank sequences by the nucleotide BLASTN program (https://blast.ncbi.nlm.nih.gov). New sequences were submitted to GenBank (*cox*1 gene: OR145129–OR145131, 16S rRNA gene: OR145132–OR145134, 12S rRNA gene: OR145138–OR145139).

### Data curation and statistical analysis

All tick-host associations can be found in Supplementary Table 1. Birds were categorized according to their feeding place, minimum and maximum body mass, migration habits, and habitats according to ornithological data and previous reports^14,15,28^ (Supplementary Table 3). In order to categorize birds according to their feeding places, “Above ground” category was created. Birds belonging to this group are feeding on (e.g.) reed trunks, bushes, or branches that do not touch the ground directly but are not far from it either. For this categorization, the expertise of our co-authors (TCS and DK) were used, as well as the available literature data^28^.

Data curation and calculation of mean and median tick intensity was done in Microsoft Office Excel. Mean and median intensities were calculated for each tick and developmental stages according to Reiczigel et al.^44^. For comparing the half-yearly activity of *I. ricinus* larvae and nymphs, and the migration habits and the habitats of the hosts of tick species (*I. ricinus, Ha. concinna*), chi-squared tests were used. Chi-squared test was also used to compare feeding places of hosts of *I. ricinus* and *Ha. concinna*. The comparison of the feeding places of the hosts of *I. ricinus* and *I. frontalis* was done with Fisher’s exact test (R -program 4.3.0.). Results were considered significant if p < 0.05.

The average body mass of each bird species was calculated as the mean of the minimum and the maximum body mass registered and listed in Supplementary Table 3. $(\mathrm{Average\, body\, mass}\hspace{0.17em}=\hspace{0.17em}\frac{\mathrm{Minimum\, body\, mass}+\mathrm{Maximum\, body\, mass}}{2}$).

The mean intensity of *Ha. concinna* and *I. ricinus* infestation was calculated for each group. The results are shown in Supplementary Fig. 4. Intensity was only calculated, if there were 10 or more infested birds in the respective category, to minimize the distortion caused by outliers. Data from one group (≤ 10g Average body mass for *Ha. concinna)* was therefore excluded (n_birds_ = 2).

In Supplementary Fig. 2, Relative, semimonthly numbers (RSN) were calculated as follows:

$${\text{RSN}}=\frac{{\text{SMN}}}{{\text{MON}}}\times 100$$

(SMN = Semimothly number of the tick species and stage; MON = number of the tick species and stage from the respective year, between March 01 - October 31).

English bird species names are capitalized following international recommendations (https://bou.org.uk/britishlist/bird-names/). In our figures and tables, HURING codes are used instead of the complete bird names. These abbreviations are clarified in Supplementary Table 3.

### Ethical approval

The study was carried out according to the national animal welfare regulations (28/1998). License for bird ringing was provided by the National Inspectorate for Environment and Nature (No 14/3858-9/2012.). License for sample collection was provided by the Central Danube Valley Environmental Protection and Nature Conservation Inspectorate (KDV-KTF) (No KTF:27251-1/2014.). Where applicable, the ARRIVE guidelines were followed.

### Supplementary Information

Supplementary Legends.

Supplementary Figure 1.

Supplementary Figure 2.

Supplementary Figure 3.

Supplementary Figure 4.

Supplementary Table 1.

Supplementary Table 2.

Supplementary Table 3.

## Data Availability

The sequences generated during the current study are publicly available in GenBank (https://www.ncbi.nlm.nih.gov/genbank/). The following sequences were submitted: *cox*1 gene: OR145129–OR145131, 16S rRNA gene: OR145132–OR145134, 12S rRNA gene: OR145138–OR145139. All other relevant data are included in the manuscript and its supplementary files.
