# Supplementary material for: Long term evaluation of factors influencing the association of ixodid ticks with birds in Central Europe, Hungary
Source: Sci Rep. 2024 Feb 29;14:4958. doi: 10.1038/s41598-024-55021-9 (PMC10902401; doi:10.1038/s41598-024-55021-9)
Supplement: Supplementary file 1 — Supplementary Legends. [file 41598_2024_55021_MOESM1_ESM.docx]

**Supplementary Figure 1.**

Tick-host associations visualized on a visweb matrix.

**Supplementary Figure 2.**

Temporal occurrence of *Ixodes ricinus* and *Haemaphysalis concinna* larvae and nymphs each year from the beginning of March to the end of October. Relative, semi-monthly numbers (RSN) were calculated as follows: $\mathrm{RSN} =\frac{\mathrm{SMN}}{\mathrm{MON}}$ X 100 (SMN= Semi-monthly number of the tick species and stage; MON = number of the tick species and stage from the respective year, between March 01 - October 31)

**Supplementary Figure 3.**

All *Ixodes frontalis* ticks collected in Ócsa, between 2015 and 2022. Our main sample collection period started at the first days March and ended in the beginning of November each year.

**Supplementary Figure 4.**

Mean intensity of *Ixodes ricinus* and *Haemaphysalis concinna* subadults, according to the body size of their hosts. Darker color implies higher intensity.
