# Supplementary material for: Long term evaluation of factors influencing the association of ixodid ticks with birds in Central Europe, Hungary
Source: Sci Rep. 2024 Feb 29;14:4958. doi: 10.1038/s41598-024-55021-9 (PMC10902401; doi:10.1038/s41598-024-55021-9)
Supplement: Supplementary file 3 — Supplementary Figure 2. [file 41598_2024_55021_MOESM3_ESM.pdf]

2015 *Ixodes ricinus*

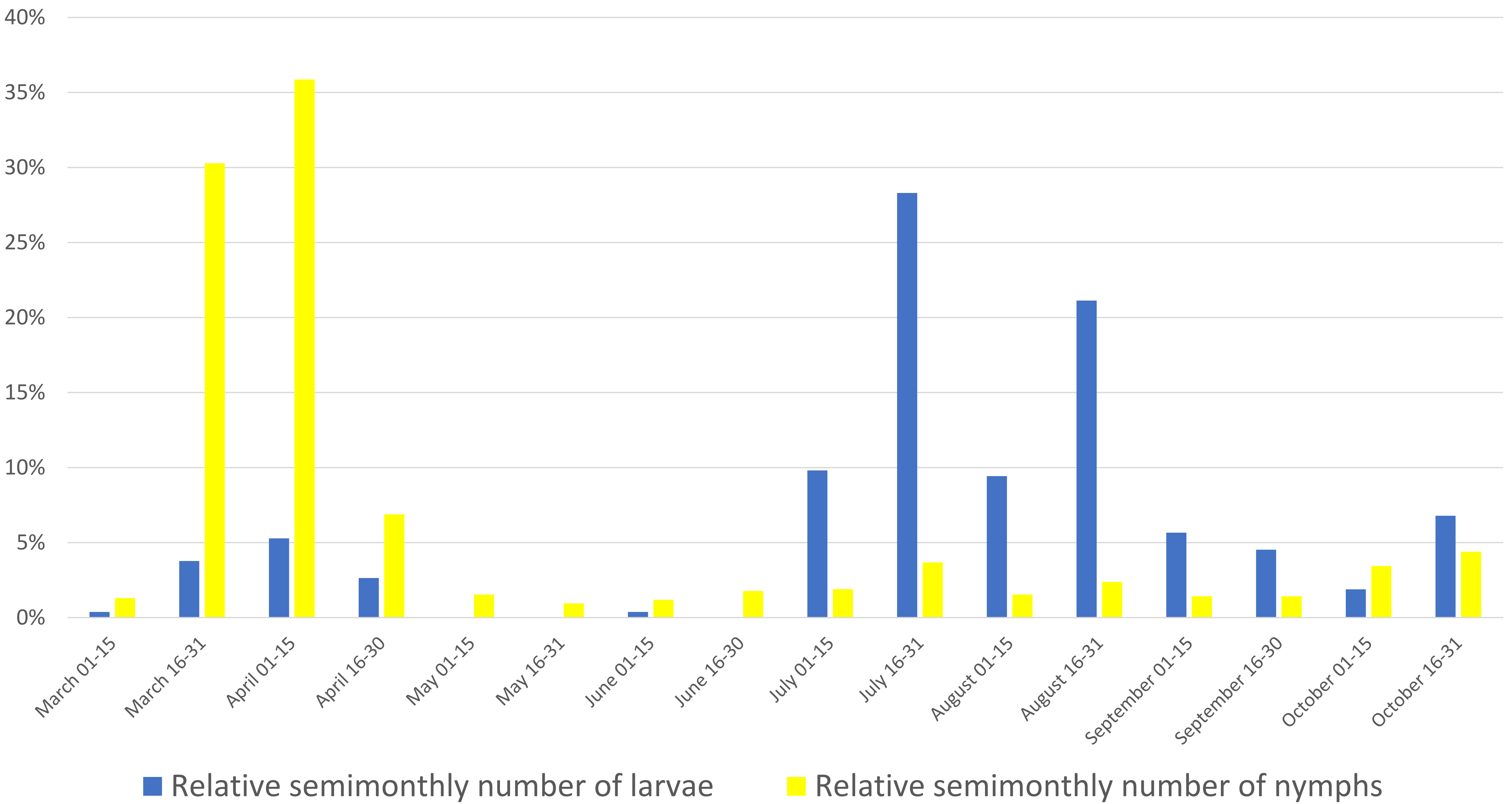

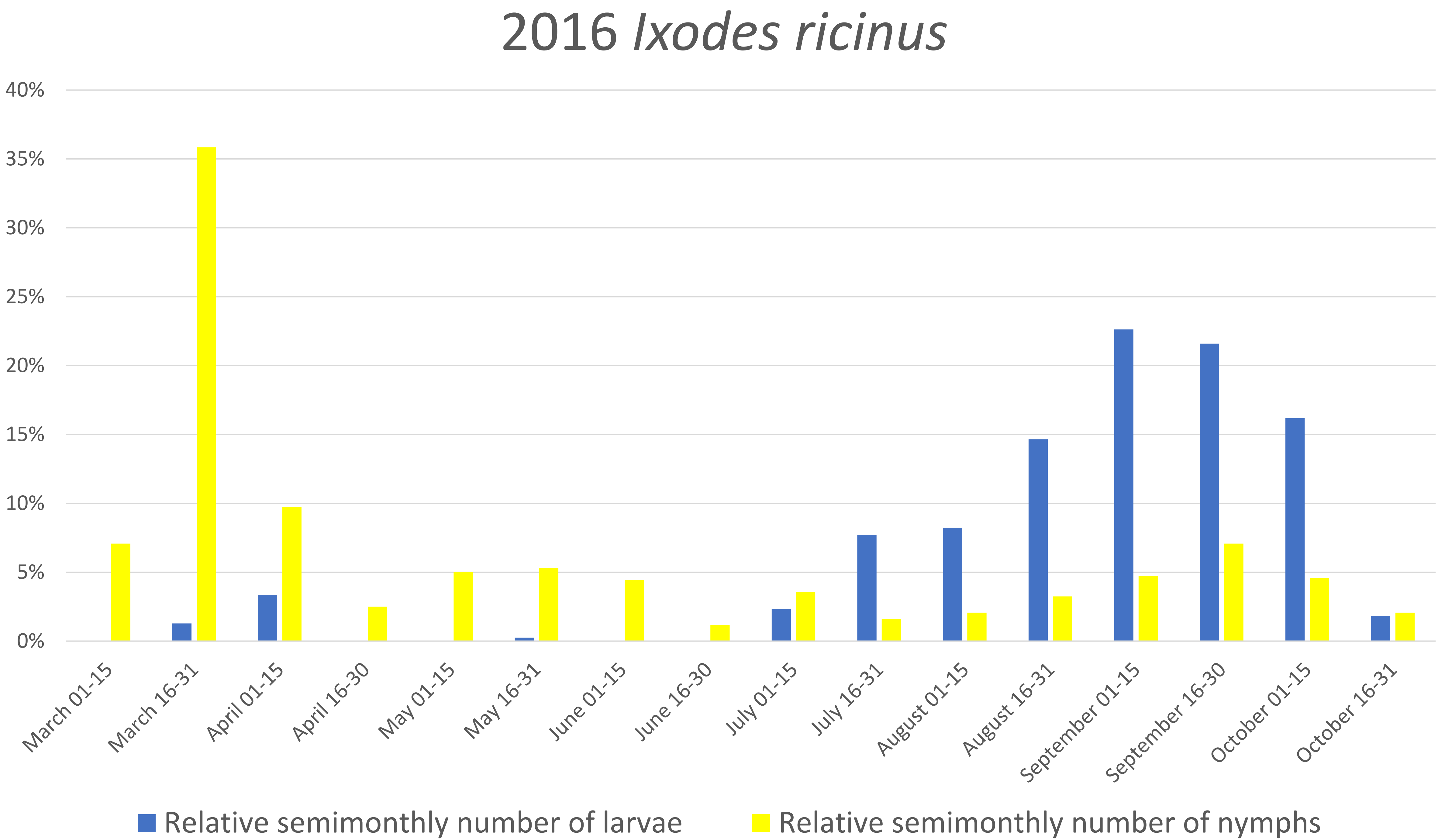

2016 *Ixodes ricinus*
40%
35%
30%
25%
20%
15%
10%
5%
0%
March 01-15
March 16-31
April 01-15
April 16-30
May 01-15
May 16-31
June 01-15
June 16-30
July 01-15
July 16-31
August 01-15
August 16-31
September 01-15
September 16-30
October 01-15
October 16-31
Relative semimonthly number of larvae
Relative semimonthly number of nymphs

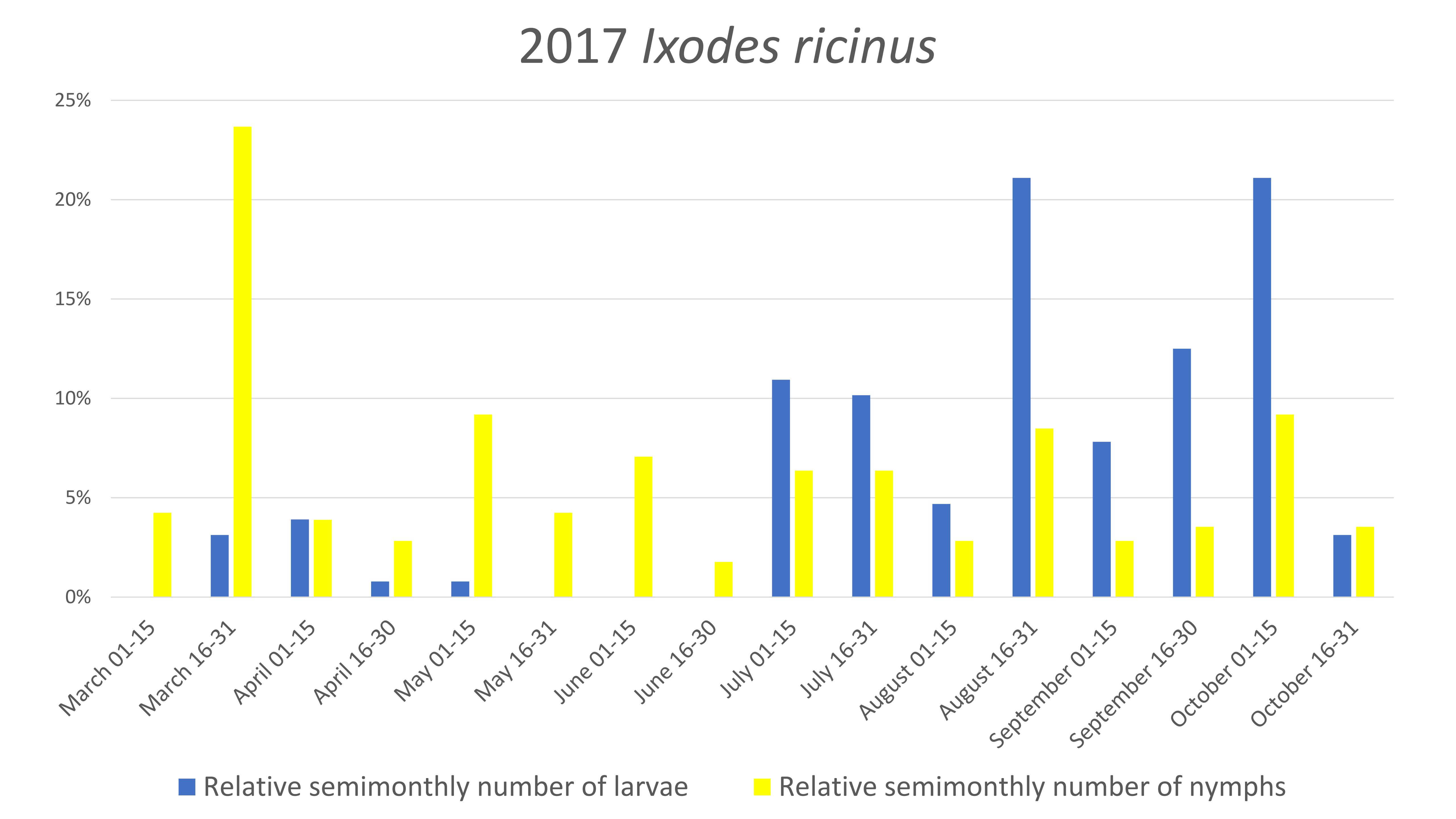
2017 *Ixodes ricinus*
25%
20%
15%
10%
5%
0%
March 01-15
March 16-31
April 01-15
April 16-30
May 01-15
May 16-31
June 01-15
June 16-30
July 01-15
July 16-31
August 01-15
August 16-31
September 01-15
September 16-30
October 01-15
October 16-31
Relative semimonthly number of larvae
Relative semimonthly number of nymphs

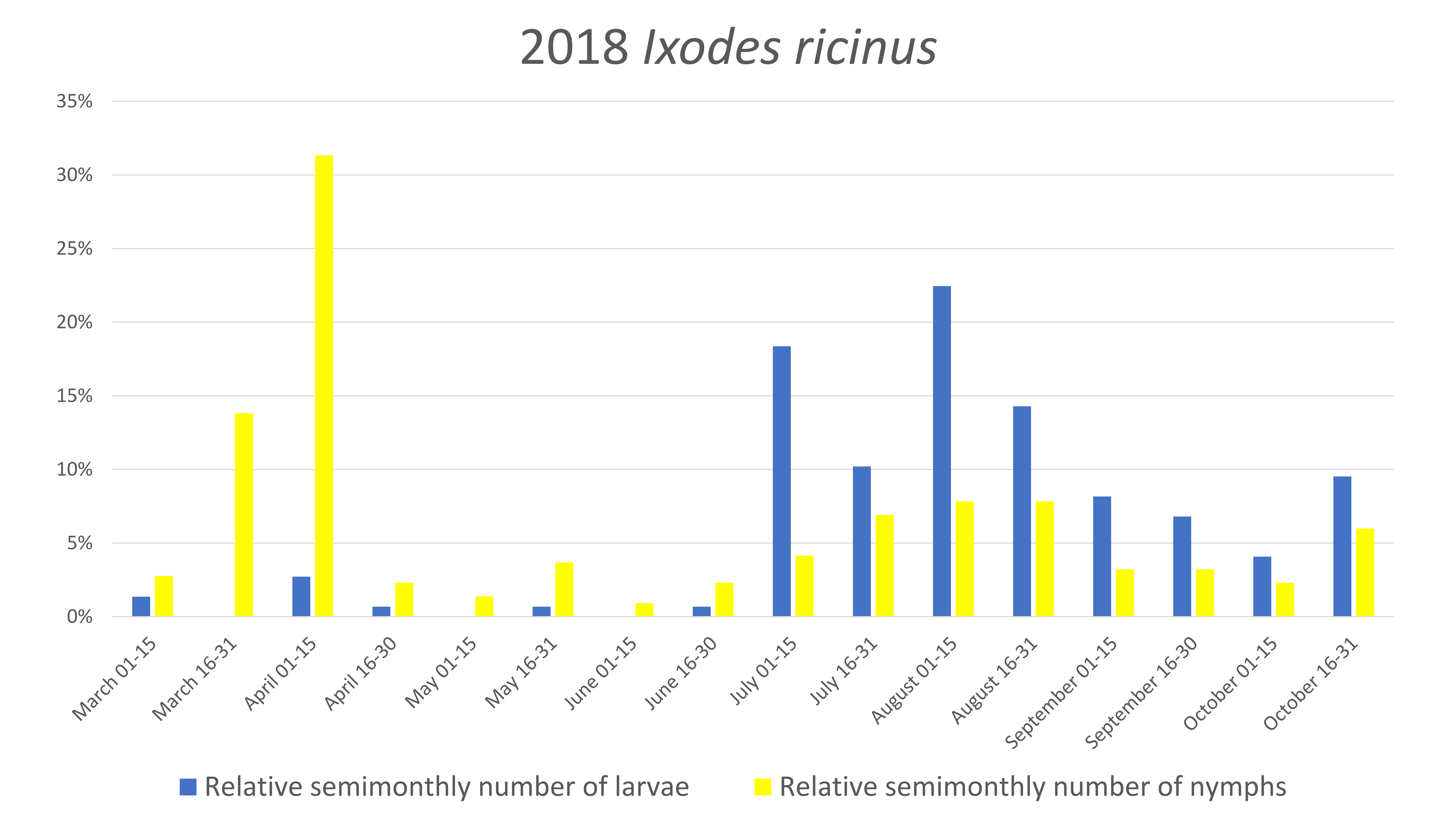

2018 *Ixodes ricinus*
35%
30%
25%
20%
15%
10%
5%
0%
March 01-15
March 16-31
April 01-15
April 16-30
May 01-15
May 16-31
June 01-15
June 16-30
July 01-15
July 16-31
August 01-15
August 16-31
September 01-15
September 16-30
October 01-15
October 16-31
Relative semimonthly number of larvae
Relative semimonthly number of nymphs

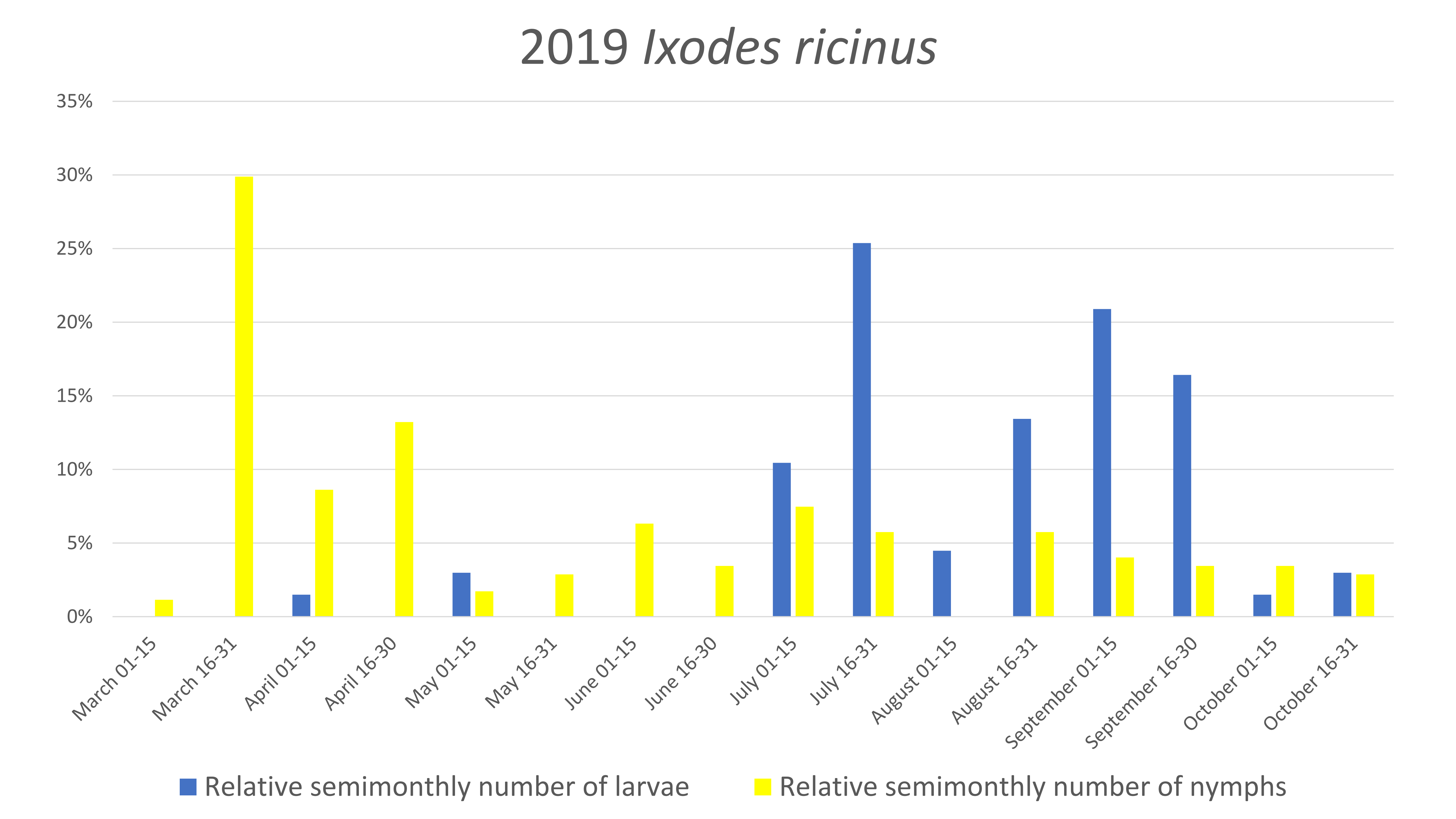

2019 *Ixodes ricinus*
35%
30%
25%
20%
15%
10%
5%
0%
March 01-15
March 16-31
April 01-15
April 16-30
May 01-15
May 16-31
June 01-15
June 16-30
July 01-15
July 16-31
August 01-15
August 16-31
September 01-15
September 16-30
October 01-15
October 16-31
Relative semimonthly number of larvae
Relative semimonthly number of nymphs

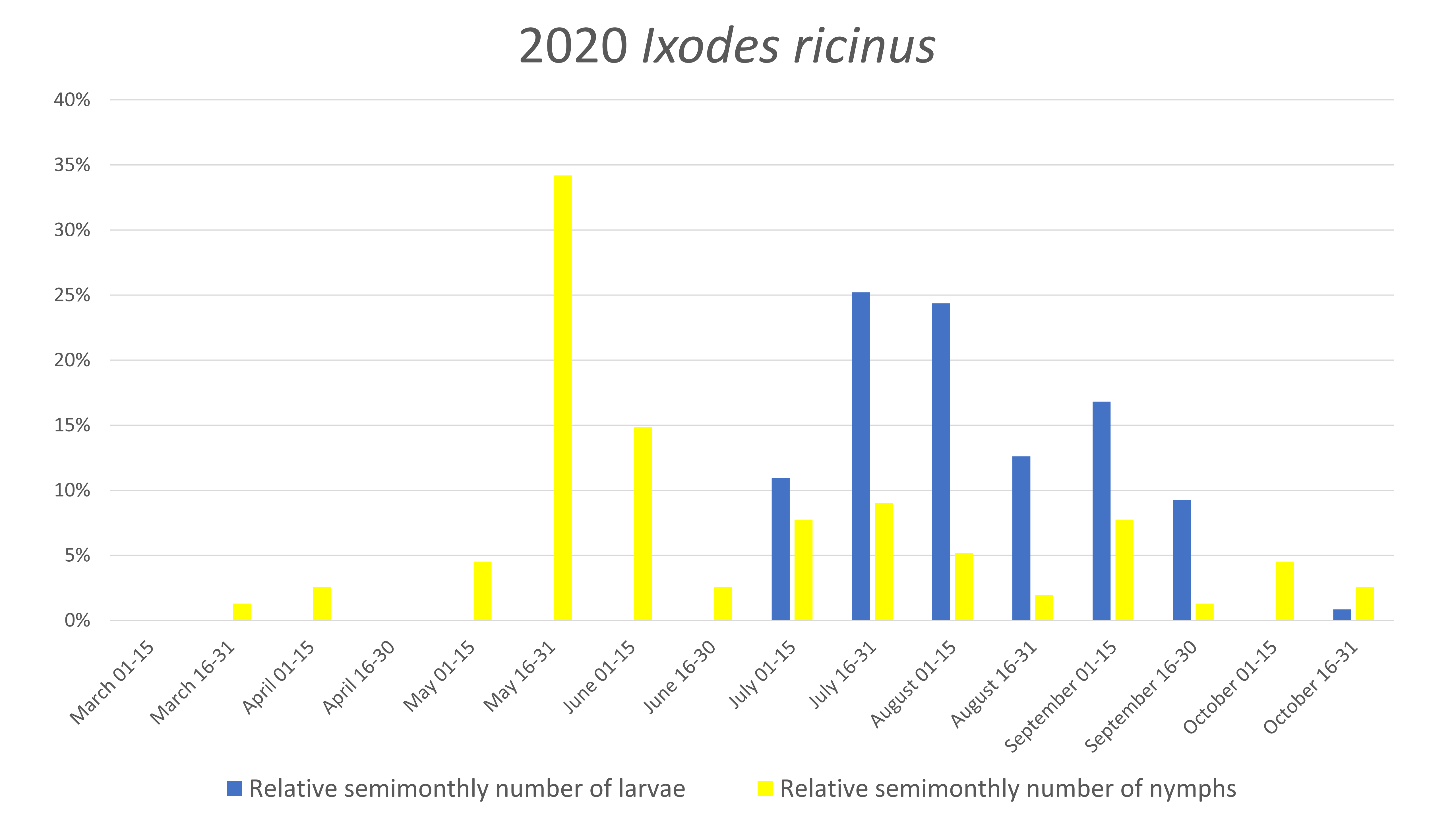

2020 *Ixodes ricinus*
40%
35%
30%
25%
20%
15%
10%
5%
0%
March 01-15
March 16-31
April 01-15
April 16-30
May 01-15
May 16-31
June 01-15
June 16-30
July 01-15
July 16-31
August 01-15
August 16-31
September 01-15
September 16-30
October 01-15
October 16-31
Relative semimonthly number of larvae
Relative semimonthly number of nymphs

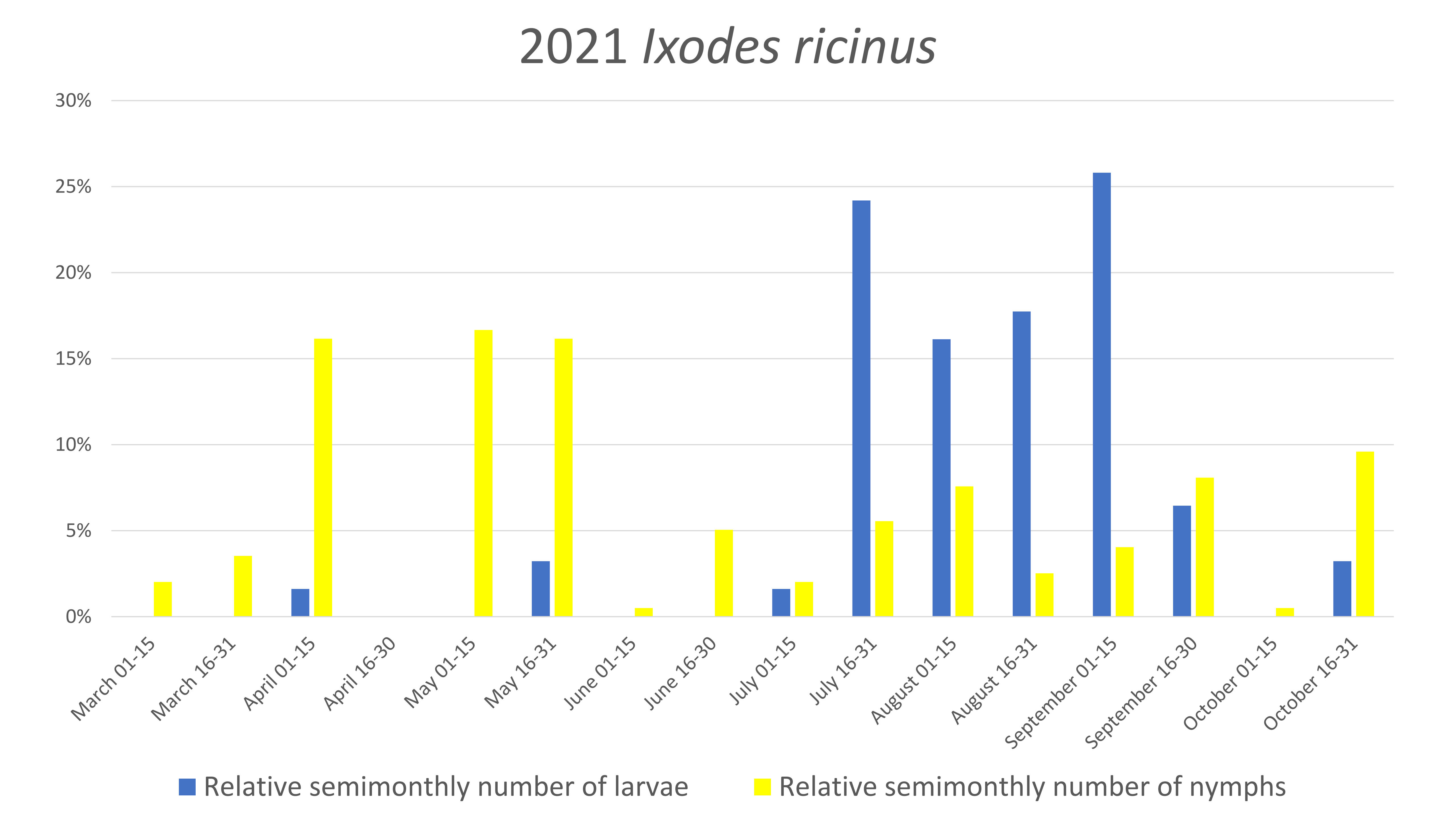
2021 *Ixodes ricinus*
30%
25%
20%
15%
10%
5%
0%
March 01-15
March 16-31
April 01-15
April 16-30
May 01-15
May 16-31
June 01-15
June 16-30
July 01-15
July 16-31
August 01-15
August 16-31
September 01-15
September 16-30
October 01-15
October 16-31
Relative semimonthly number of larvae
Relative semimonthly number of nymphs

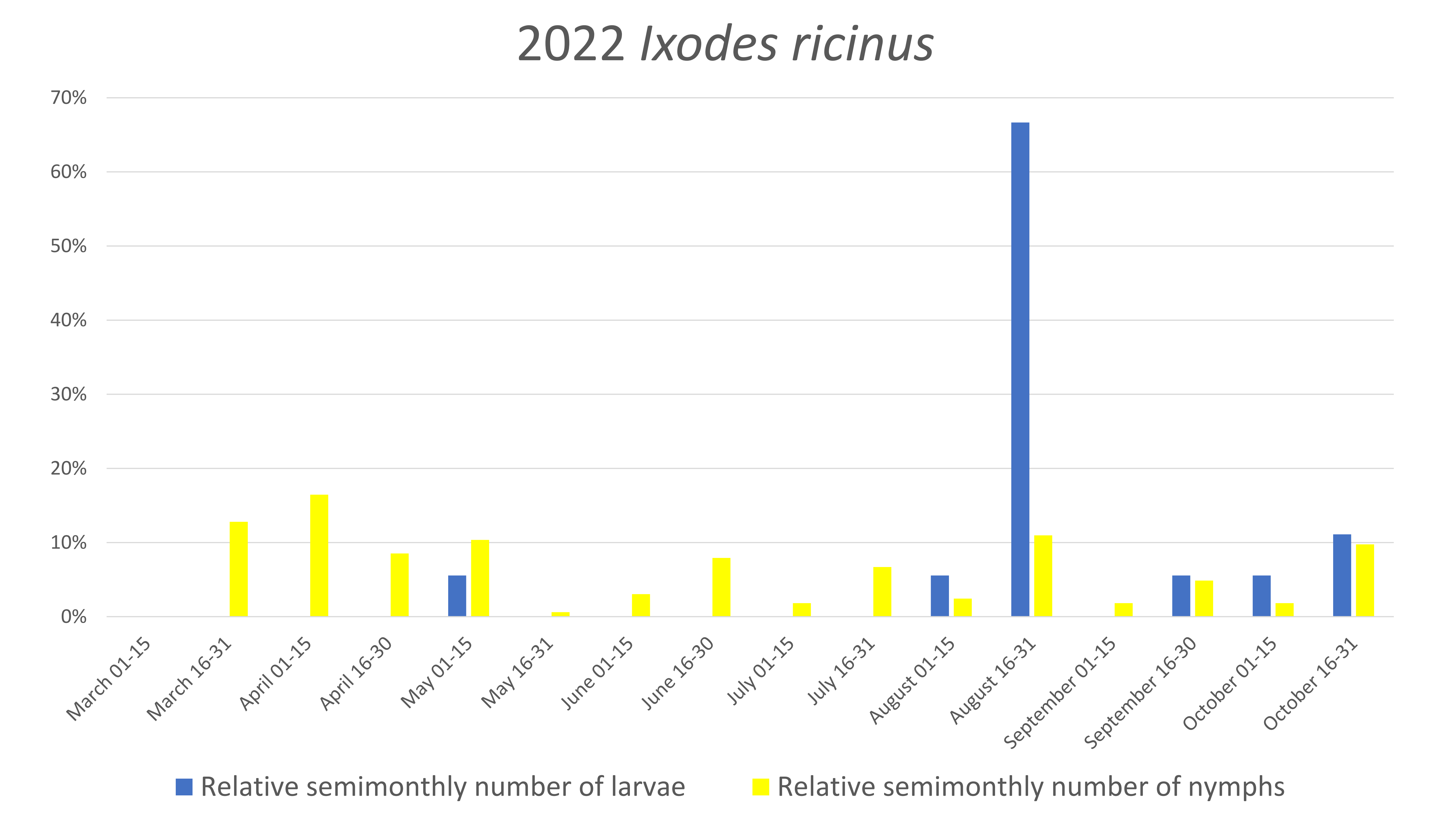

2022 *Ixodes ricinus*
70%
60%
50%
40%
30%
20%
10%
0%
March 01-15
March 16-31
April 01-15
April 16-30
May 01-15
May 16-31
June 01-15
June 16-30
July 01-15
July 16-31
August 01-15
August 16-31
September 01-15
September 16-30
October 01-15
October 16-31
Relative semimonthly number of larvae
Relative semimonthly number of nymphs

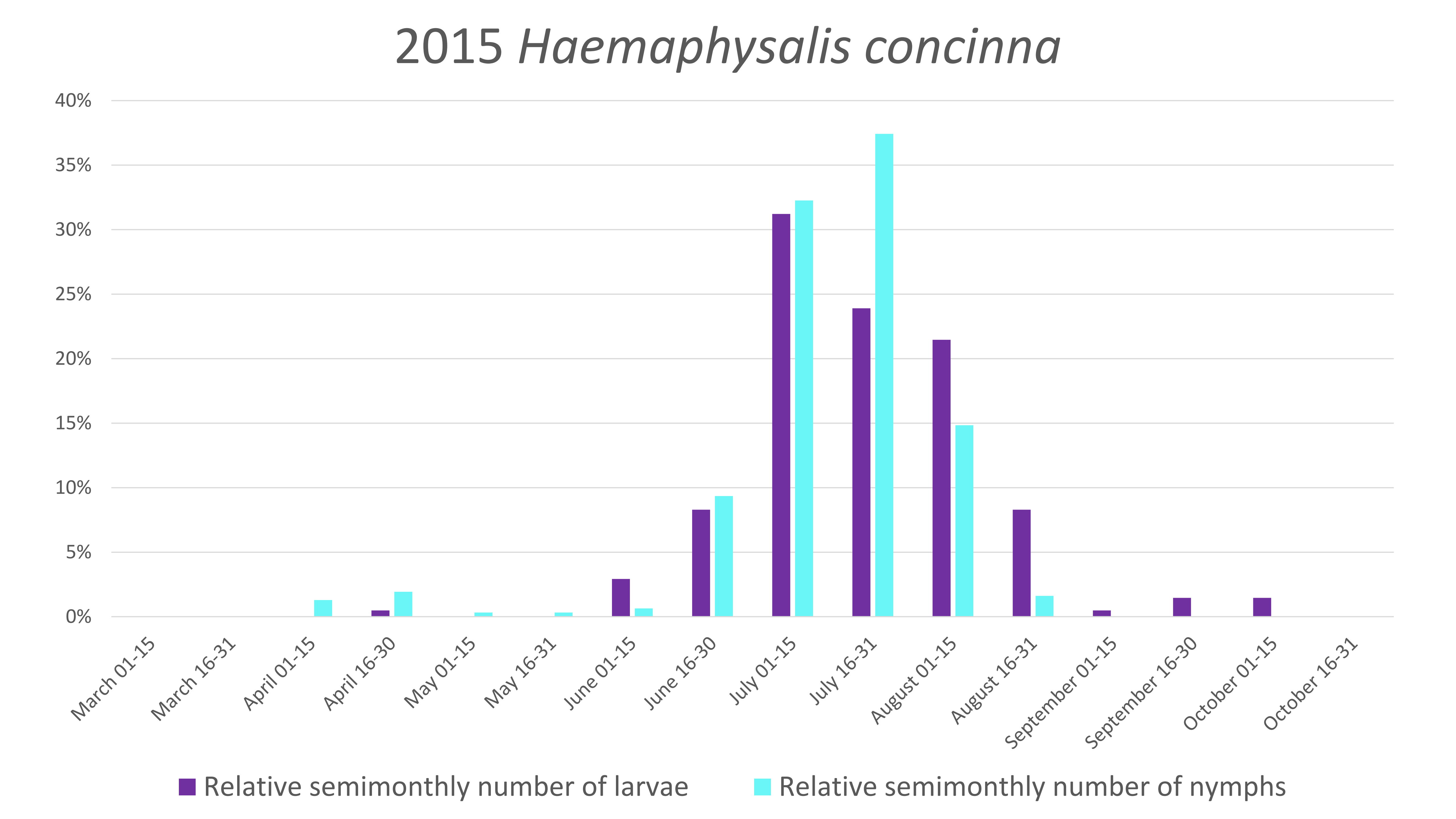
2015 Haemaphysalis concinna
40%
35%
30%
25%
20%
15%
10%
5%
0%
March 01-15
March 16-31
April 01-15
April 16-30
May 01-15
May 16-31
June 01-15
June 16-30
July 01-15
July 16-31
August 01-15
August 16-31
September 01-15
September 16-30
October 01-15
October 16-31
Relative semimonthly number of larvae
Relative semimonthly number of nymphs

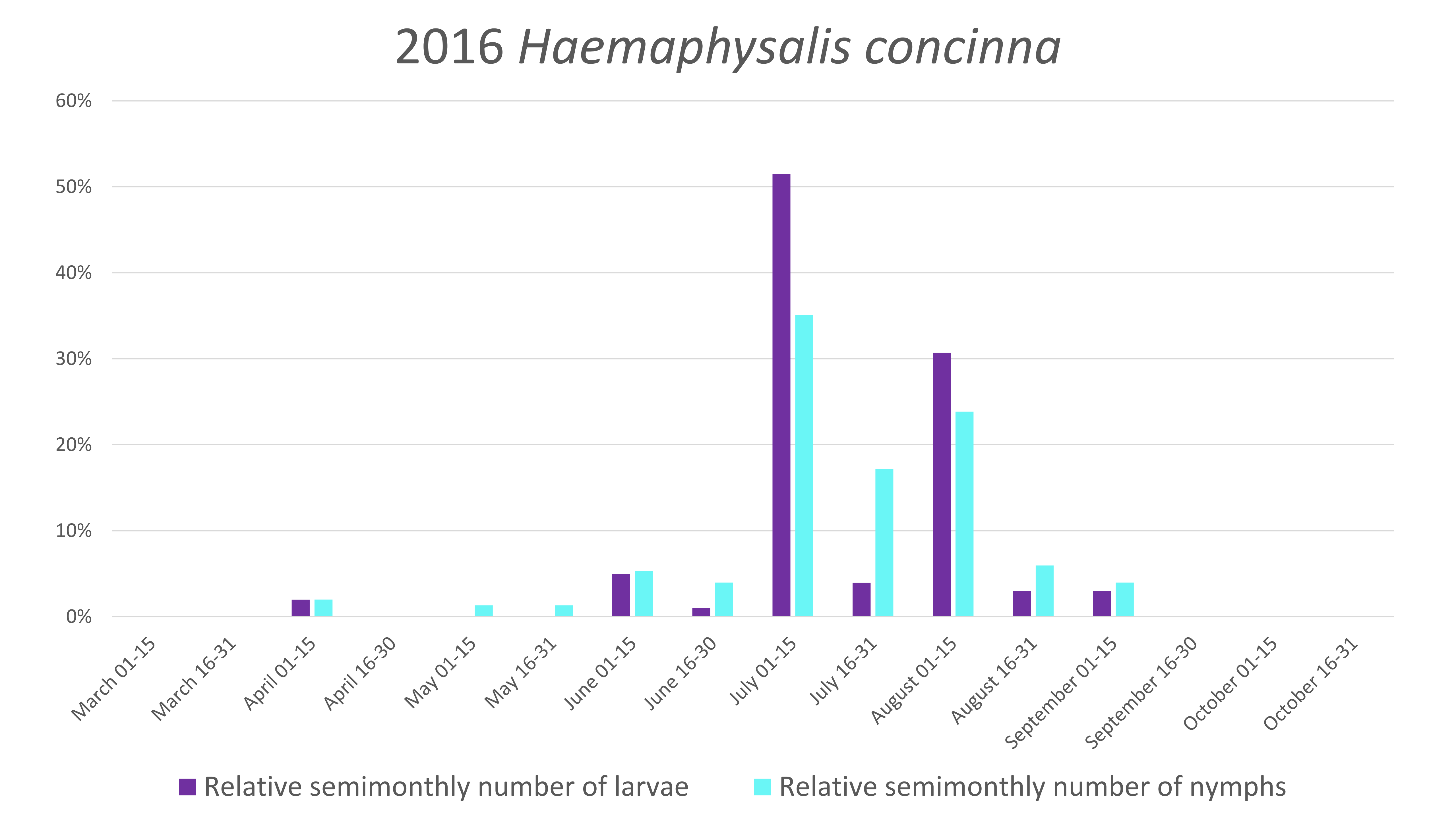
2016 *Haemaphysalis concinna*
60%
50%
40%
30%
20%
10%
0%
March 01-15
March 16-31
April 01-15
April 16-30
May 01-15
May 16-31
June 01-15
June 16-30
July 01-15
July 16-31
August 01-15
August 16-31
September 01-15
September 16-30
October 01-15
October 16-31
Relative semimonthly number of larvae
Relative semimonthly number of nymphs

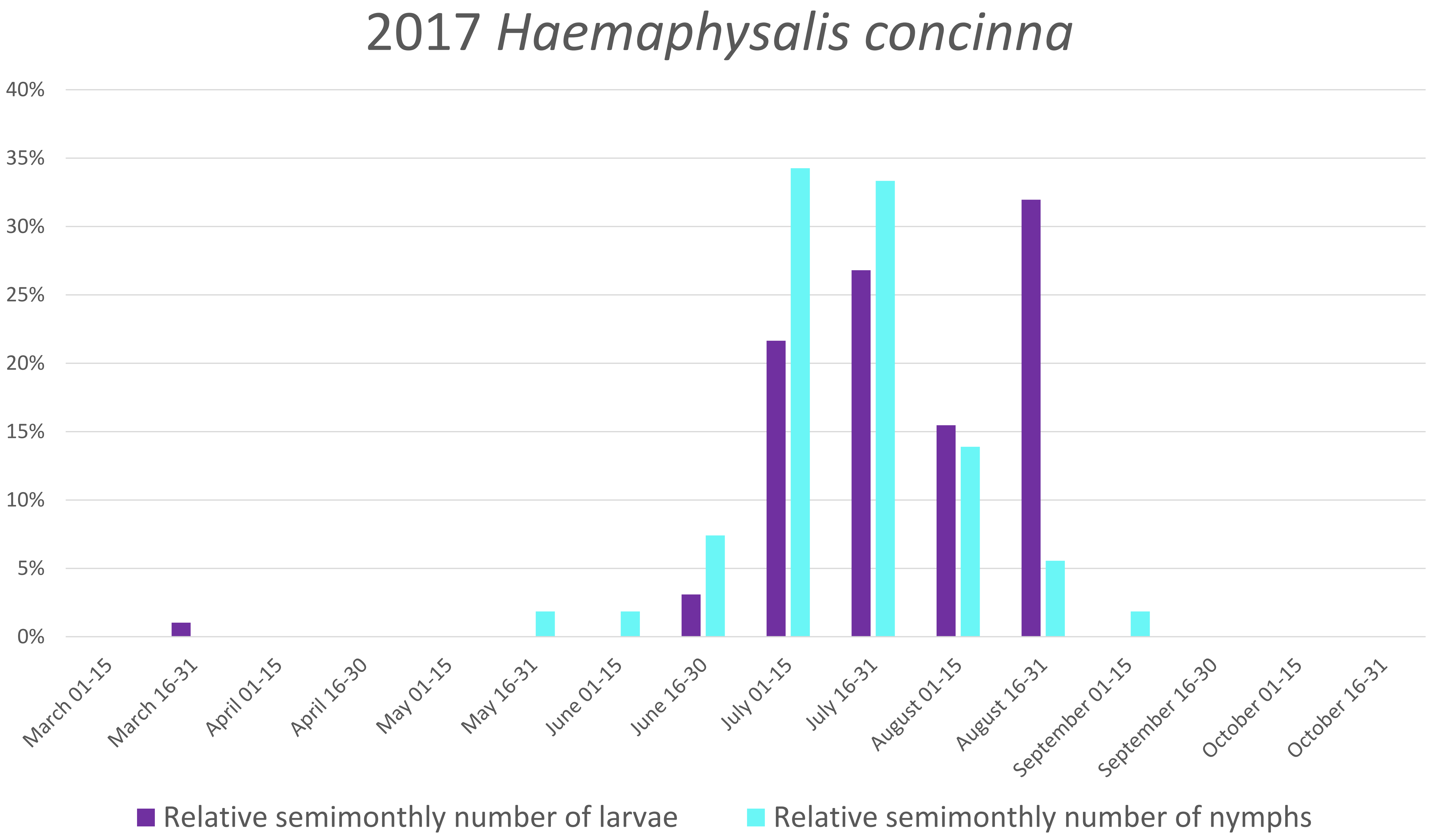

2017 *Haemaphysalis concinna*
40%
35%
30%
25%
20%
15%
10%
5%
0%
March 01-15
March 16-31
April 01-15
April 16-30
May 01-15
May 16-31
June 01-15
June 16-30
July 01-15
July 16-31
August 01-15
August 16-31
September 01-15
September 16-30
October 01-15
October 16-31
Relative semimonthly number of larvae
Relative semimonthly number of nymphs

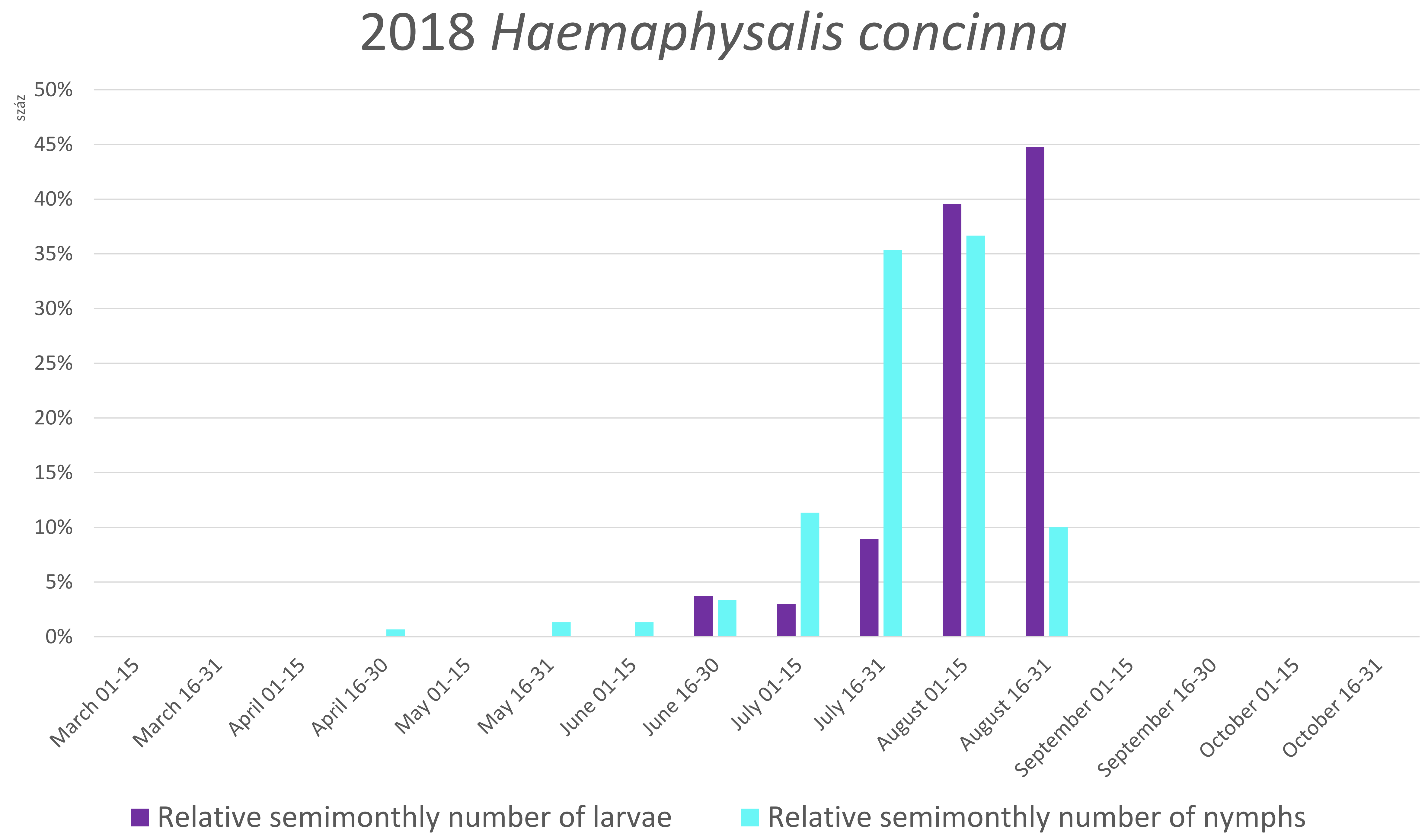
2018 *Haemaphysalis concinna*
száz
50%
45%
40%
35%
30%
25%
20%
15%
10%
5%
0%
March 01-15
March 16-31
April 01-15
April 16-30
May 01-15
May 16-31
June 01-15
June 16-30
July 01-15
July 16-31
August 01-15
August 16-31
September 01-15
September 16-30
October 01-15
October 16-31
Relative semimonthly number of larvae
Relative semimonthly number of nymphs

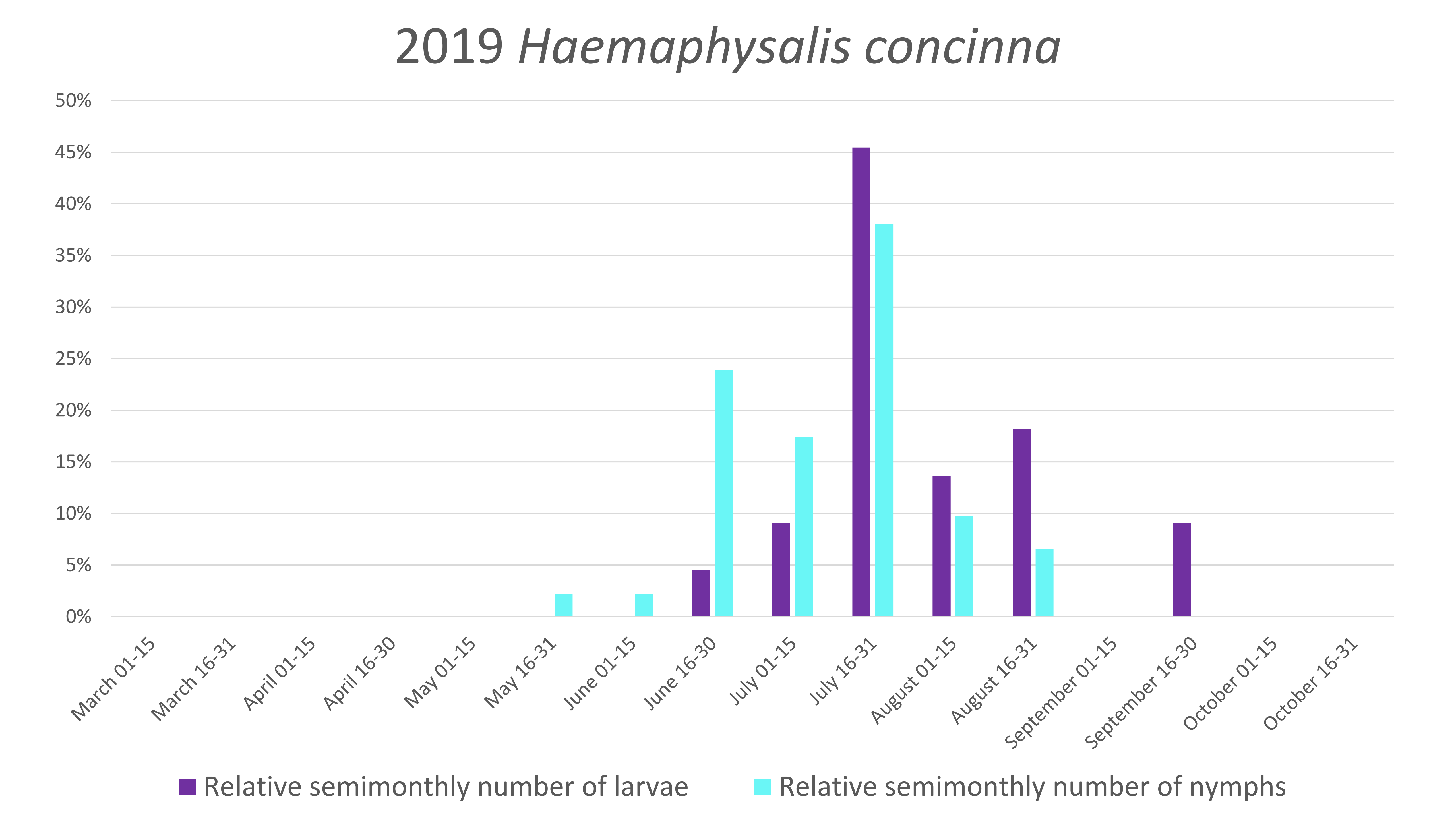
2019 *Haemaphysalis concinna*
50%
45%
40%
35%
30%
25%
20%
15%
10%
5%
0%
March 01-15
March 16-31
April 01-15
April 16-30
May 01-15
May 16-31
June 01-15
June 16-30
July 01-15
July 16-31
August 01-15
August 16-31
September 01-15
September 16-30
October 01-15
October 16-31
Relative semimonthly number of larvae
Relative semimonthly number of nymphs

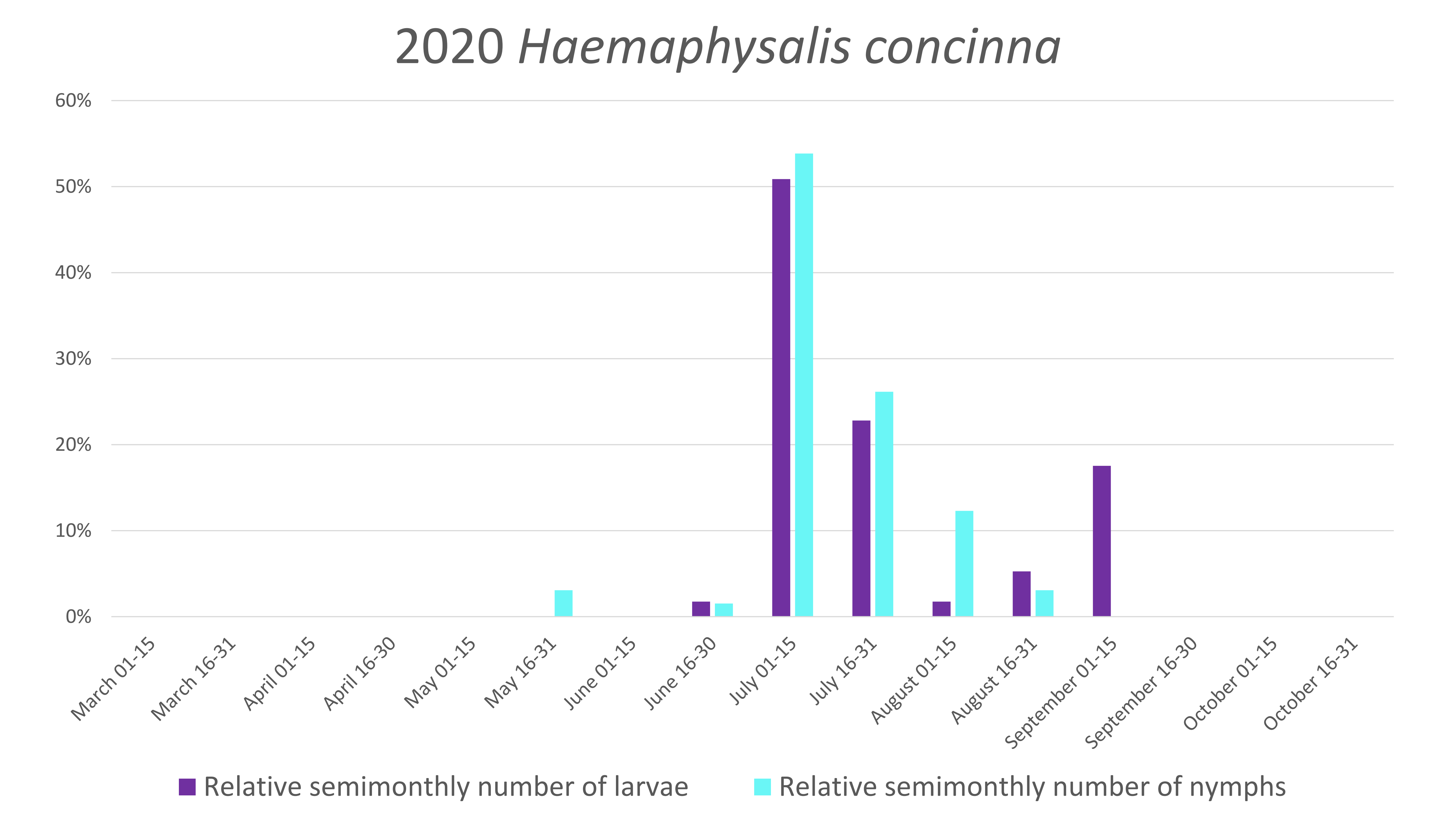
2020 Haemaphysalis concinna
60%
50%
40%
30%
20%
10%
0%
March 01-15
March 16-31
April 01-15
April 16-30
May 01-15
May 16-31
June 01-15
June 16-30
July 01-15
July 16-31
August 01-15
August 16-31
September 01-15
September 16-30
October 01-15
October 16-31
Relative semimonthly number of larvae
Relative semimonthly number of nymphs

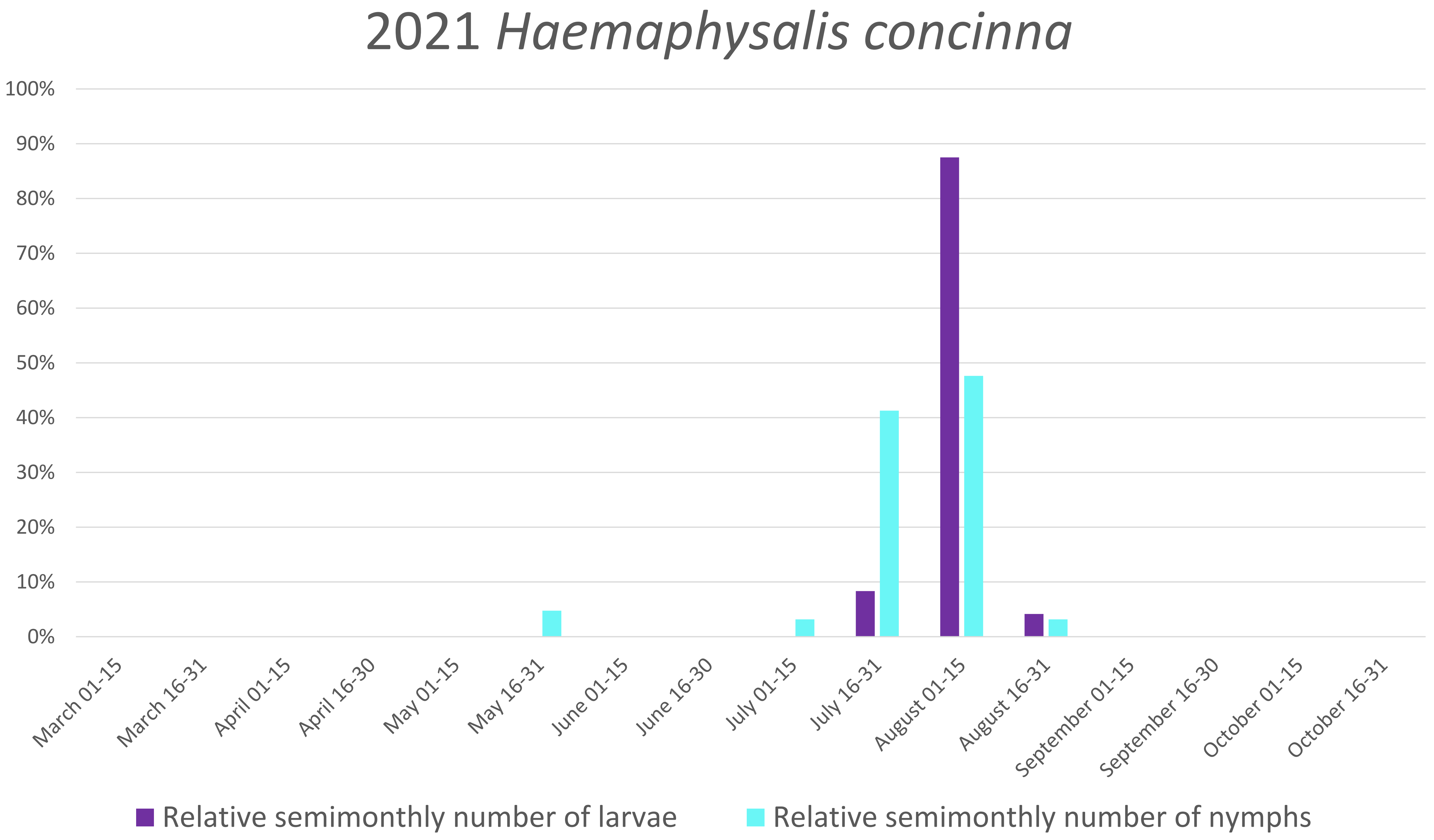

2021 *Haemaphysalis concinna*
100%
90%
80%
70%
60%
50%
40%
30%
20%
10%
0%
March 01-15
March 16-31
April 01-15
April 16-30
May 01-15
May 16-31
June 01-15
June 16-30
July 01-15
July 16-31
August 01-15
August 16-31
September 01-15
September 16-30
October 01-15
October 16-31
Relative semimonthly number of larvae
Relative semimonthly number of nymphs

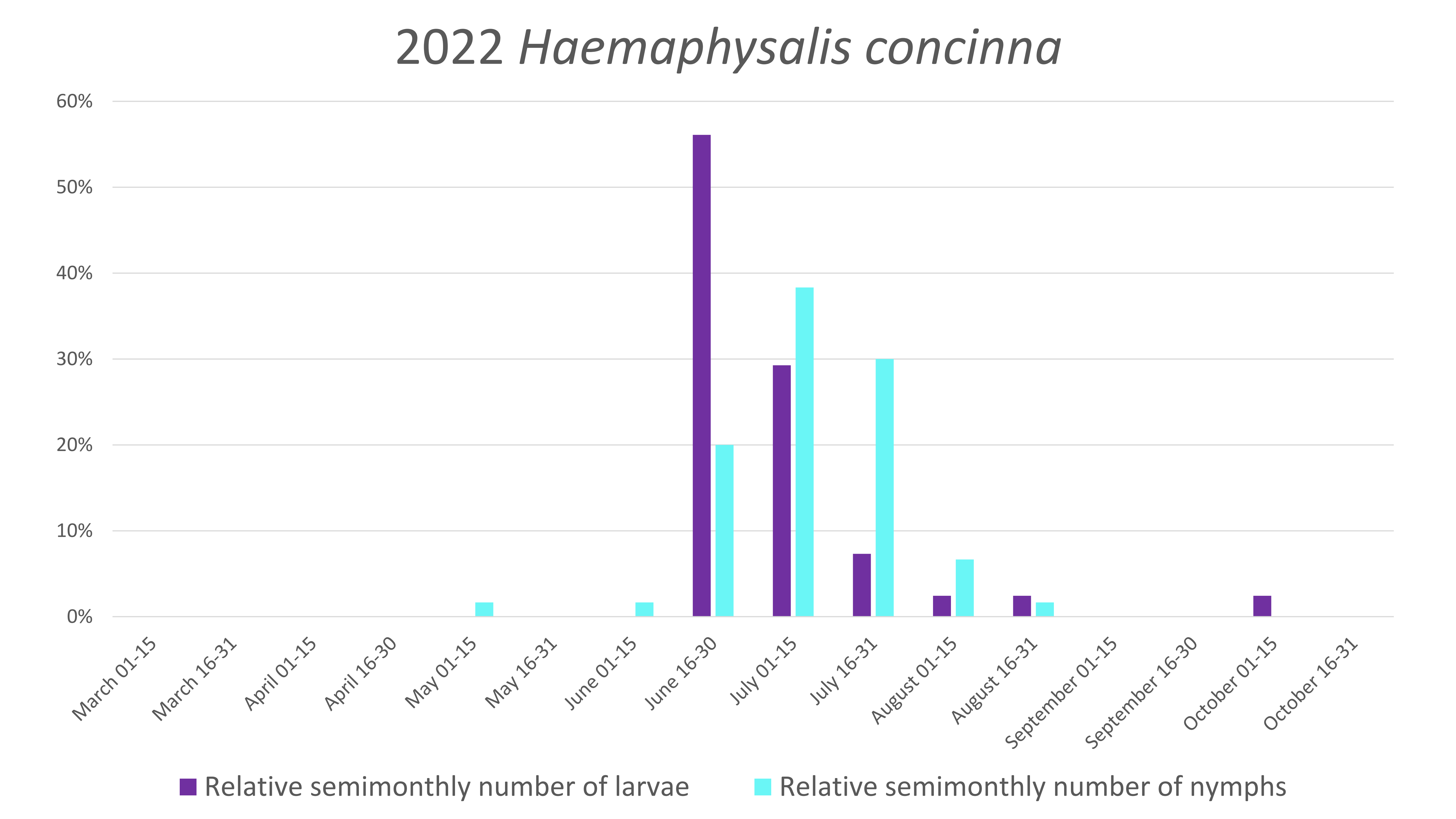
2022 Haemaphysalis concinna
60%
50%
40%
30%
20%
10%
0%
March 01-15
March 16-31
April 01-15
April 16-30
May 01-15
May 16-31
June 01-15
June 16-30
July 01-15
July 16-31
August 01-15
August 16-31
September 01-15
September 16-30
October 01-15
October 16-31
Relative semimonthly number of larvae
Relative semimonthly number of nymphs
