# Supplementary figures and images for: Long term evaluation of factors influencing the association of ixodid ticks with birds in Central Europe, Hungary
Source: Sci Rep. 2024 Feb 29;14:4958. doi: 10.1038/s41598-024-55021-9 (PMC10902401; doi:10.1038/s41598-024-55021-9)

# Total numbers of *Ixodes frontalis* ticks collected from birds

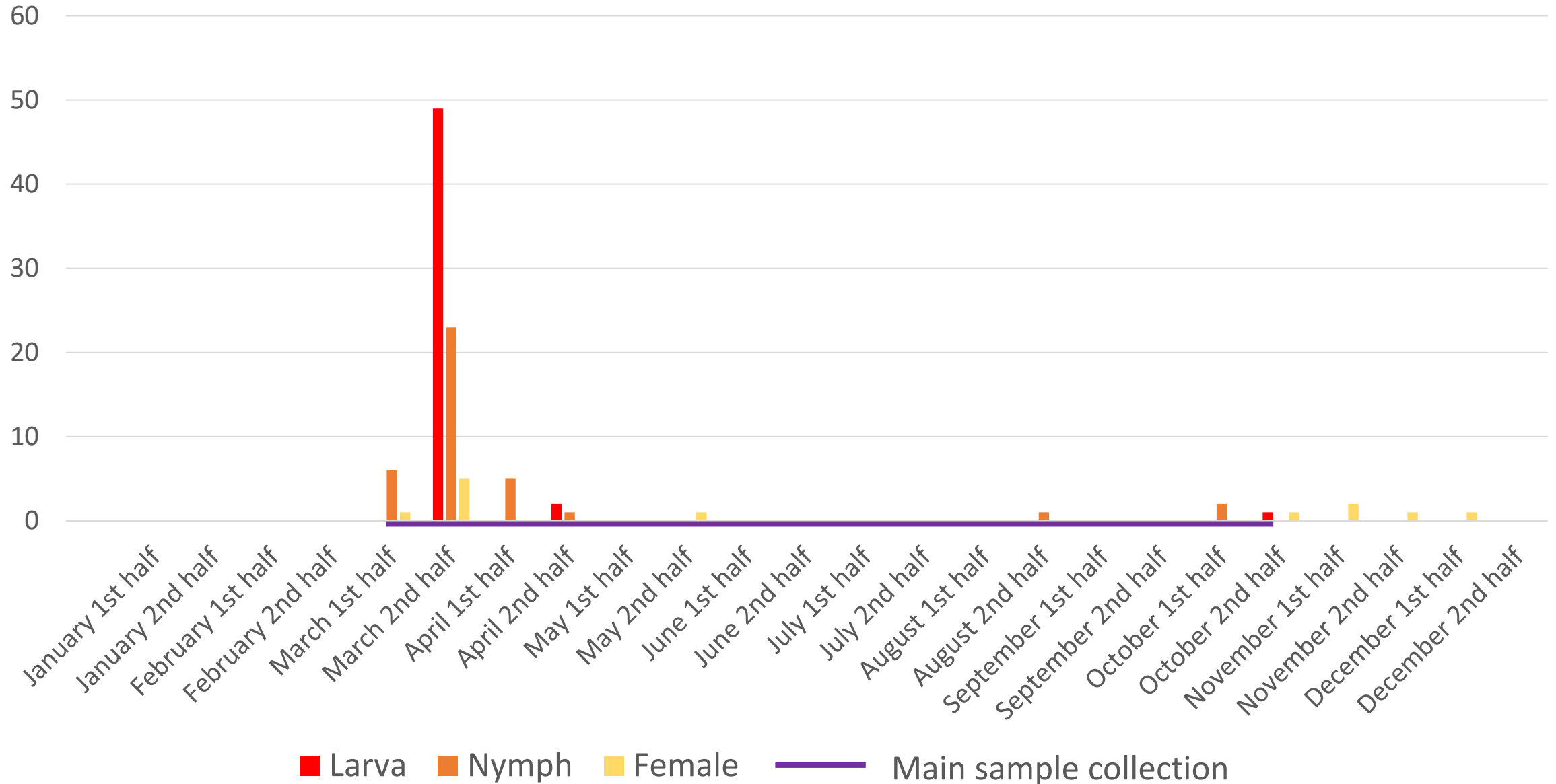

Supplement: Supplementary file 4 — Supplementary Figure 3. [file 41598_2024_55021_MOESM4_ESM.pdf]
