# Supplementary material for: Long term evaluation of factors influencing the association of ixodid ticks with birds in Central Europe, Hungary
Source: Sci Rep. 2024 Feb 29;14:4958. doi: 10.1038/s41598-024-55021-9 (PMC10902401; doi:10.1038/s41598-024-55021-9)
Supplement: Supplementary file 5 — Supplementary Figure 4. [file 41598_2024_55021_MOESM5_ESM.pdf]

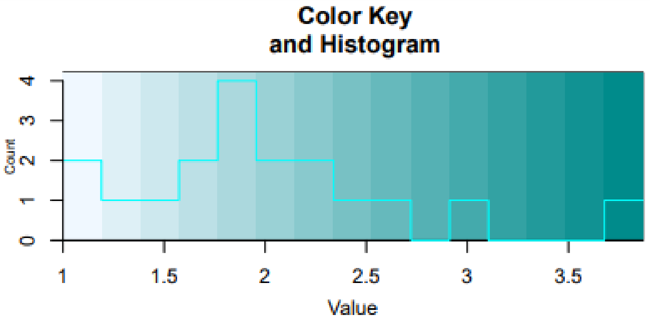

Mean tick intensity on birds, according to their average bodymass

	<=10g	10.1–20g	20.1–50g	50.1–100g	>100g
Ixodes ricinus Larva	Mean = 1.76<br>n_{birds} = 29	Mean = 1.96<br>n_{birds} = 319	Mean = 1.91<br>n_{birds} = 193	Mean =2.65<br>n_{birds} = 31	Mean = 2.29<br>n_{birds} = 45
Ixodes ricinus Nymph	Mean = 1.17<br>n_{birds} = 52	Mean = 1.34<br>n_{birds} = 600	Mean = 1.79<br>n_{birds} = 331	Mean = 2.17<br>n_{birds} = 138	Mean = 3.06<br>n_{birds} = 321
Haemaphysalis coninna Larva	NA	Mean = 3.97<br>n_{birds} = 152	Mean = 1.44<br>n_{birds} = 18	Mean = 1.82<br>n_{birds} = 11	Mean = 2.47<br>n_{birds} = 19
Haemaphysalis concinna Nymph	NA	Mean = 2.07<br>n_{birds} = 379	Mean = 1.5<br>n_{birds} = 38	Mean = 1.80<br>n_{birds} = 46	Mean = 1.57<br>n_{birds} = 54
