# Supplementary material for: Long term evaluation of factors influencing the association of ixodid ticks with birds in Central Europe, Hungary
Source: Sci Rep. 2024 Feb 29;14:4958. doi: 10.1038/s41598-024-55021-9 (PMC10902401; doi:10.1038/s41598-024-55021-9)
Supplement: Supplementary file 6 — Supplementary Table 1. [file 41598_2024_55021_MOESM6_ESM.pdf]

Supplementary Table 1: All ticks found between March 2015 and November 2022, according to their host species and date of collection

SAMPLE ID	HOST	DATE	*I. ricinus* L	*I. ricinus* N	*I. frontalis* L	*I. frontalis* N	*I. frontalis* F	*I. festai* F	*I. festai* M	*I. lividus* N	*I. lividus* F	*Ha. concinna* L	*Ha. concinna* N	*Ha. punctata* L	*I. arboricola* N	*Hyalomma* sp. N	*D. reticulatus* F
HS274	*TUR MER*	2015.03.21		2													
HS275	*TUR MER*	2015.03.21		3													
HS276	*TUR MER*	2015.03.11		2													
HS277	*CAR CHL*	2015.03.11		2													
HS278	*TUR MER*	2015.03.11		1													
HS279	*TUR MER*	2015.03.13	1	1													
HS280	*TUR MER*	2015.03.14		1													
HS281	*TUR MER*	2015.03.14		1													
HS282	*TUR MER*	2015.03.14		1													
HS283	*FRI COE*	2015.03.14		1													
HS284	*TUR MER*	2015.03.15		1													
HS285	*TUR PHI*	2015.03.17	2	1													
HS286	*PAR MAJ*	2015.03.17		1													
HS287	*TUR MER*	2015.03.18		1													
HS288	*TUR MER*	2015.03.18		10													
HS289	*PRU MOD*	2015.03.18		2													
HS290	*PRU MOD*	2015.03.18		1													
HS291	*PRU MOD*	2015.03.18		2													
HS292	*TUR MER*	2015.03.19		4													
HS294	*TUR MER*	2015.03.20		1													
HS295	*PAR CAE*	2015.03.20		1													
HS296	*PAR MAJ*	2015.03.20		1													
HS297	*FRI MON*	2015.03.21		1													
HS298	*PAR MAJ*	2015.03.21		1													
HS299	*ERI RUB*	2015.03.22		1													
HS300	*TUR MER*	2015.03.22		4													
HS302	*PAR MAJ*	2015.03.22	1	3													
HS303	*TUR PHI*	2015.03.22		1													
HS304	*TUR PHI*	2015.03.22		1													
HS305	*TUR PHI*	2015.03.22		1													
HS306	*FRI COE*	2015.03.22				1											
HS307	*TUR MER*	2015.03.23		3													
HS308	*TUR MER*	2015.03.23	1	1													
HS309	*PAR MAJ*	2015.03.23		2													
HS310	*TUR PHI*	2015.03.23		2													
HS311	*TUR PHI*	2015.03.23		2													
HS312	*TUR MER*	2015.03.23	1	1													
HS313	*TUR MER*	2015.03.24		4													
HS314	*TUR MER*	2015.03.24		2													
HS315	*TUR MER*	2015.03.24	1	3													
HS316	*TUR MER*	2015.03.24		2													
HS317	*TRO TRO*	2015.03.24		1													
HS318	*PAR MAJ*	2015.03.24		2													
HS320	*PAR MAJ*	2015.03.25		1													
HS321	*TUR MER*	2015.03.25		1													
HS322	*TUR MER*	2015.03.25					1										
HS323	*ERI RUB*	2015.03.25			1												
HS324	*TUR MER*	2015.03.25		2													
HS325	*COC COC*	2015.03.25		4													
HS326	*TUR MER*	2015.03.25		2													
HS327	*PRU MOD*	2015.03.25		1													
HS328	*FRI MON*	2015.03.25	1	1													
HS329	*PAR MAJ*	2015.03.25		5													
HS330	*PRU MOD*	2015.03.25		6	8												
HS331	*TUR MER*	2015.03.25		18													
HS332	*TUR MER*	2015.03.25		2													
HS333	*PRU MOD*	2015.03.26		3													

SAMPLE ID	HOST	DATE	*I. ricinus* L	*I. ricinus* N	*I. frontalis* L	*I. frontalis* N	*I. frontalis* F	*I. festai* F	*I. festai* M	*I. lividus* N	*I. lividus* F	*Ha. concinna* L	*Ha. concinna* N	*Ha. punctata* L	*I. arboricola* N	*Hyalomma* sp. N	*D. reticulatus* F
HS334	*TUR MER*	2015.03.26		6													
HS335	*TUR MER*	2015.03.27		5													
HS336	*TUR MER*	2015.03.27		3													
HS337	*TUR MER*	2015.03.27						2	1								
HS338	*PRU MOD*	2015.03.27		3													
HS339	*ERI RUB*	2015.03.27				1											
HS340	*TUR MER*	2015.03.27		2													
HS341	*TUR MER*	2015.03.28		2													
HS342	*TUR ILI*	2015.03.28		1													
HS343	*TUR MER*	2015.03.28		3													
HS344	*TUR ILI*	2015.03.28		5													
HS345	*COC COC*	2015.03.28		3													
HS346	*PAR MAJ*	2015.03.28		1													
HS347	*ERI RUB*	2015.03.28		1													
HS348	*PRU MOD*	2015.03.28		3													
HS349	*PHY COL*	2015.03.28			1												
HS350	*COC COC*	2015.03.28		2													
HS351	*PAR MAJ*	2015.03.28		1													
HS352	*PRU MOD*	2015.03.28		2													
HS353	*PRU MOD*	2015.03.28		2													
HS354	*PRU MOD*	2015.03.28		1													
HS355	*TUR MER*	2015.03.28		5													
HS356	*ERI RUB*	2015.03.29		1													
HS357	*TUR MER*	2015.03.29		4													
HS358	*ERI RUB*	2015.03.29		1													
HS359	*ERI RUB*	2015.03.29		1													
HS360	*PRU MOD*	2015.03.29		5													
HS361	*PRU MOD*	2015.03.29		4													
HS362	*COC COC*	2015.03.29		2													
HS364	*TUR MER*	2015.03.29		7													
HS365	*PRU MOD*	2015.03.29		1													
HS366	*ERI RUB*	2015.03.29		1													
HS367	*TUR PHI*	2015.03.29		2													
HS368	*PRU MOD*	2015.03.30		3													
HS369	*TUR ILI*	2015.03.30	2	13													
HS370	*TUR PHI*	2015.03.30		1													
HS371	*TUR PHI*	2015.03.30		1													
HS372	*ERI RUB*	2015.03.30		2													
HS373	*ERI RUB*	2015.03.30		4													
HS374	*TUR MER*	2015.03.30			1												
HS375	*TUR MER*	2015.03.30		2													
HS376	*ERI RUB*	2015.03.30		12													
HS377	*ERI RUB*	2015.03.30		1													
HS378	*PRU MOD*	2015.03.30				1											
HS379	*PRU MOD*	2015.03.30		1													
HS380	*ERI RUB*	2015.03.30		1													
HS381	*ERI RUB*	2015.03.30		1													
HS382	*ERI RUB*	2015.03.30	1	3													
HS383	*PRU MOD*	2015.03.30		2													
HS384	*COC COC*	2015.03.30		8													
HS385	*ERI RUB*	2015.03.30				1											
HS386	*TUR MER*	2015.03.31		6													
HS387	*TUR MER*	2015.04.04		2													
HS388	*ERI RUB*	2015.04.04		1													
HS389	*TUR MER*	2015.04.04		1													
HS390	*TUR MER*	2015.04.04		1													
HS391	*ERI RUB*	2015.04.04		1													
HS392	*ERI RUB*	2015.04.04	1														

SAMPLE ID	HOST	DATE	*I. ricinus* L	*I. ricinus* N	*I. frontalis* L	*I. frontalis* N	*I. frontalis* F	*I. festai* F	*I. festai* M	*I. lividus* N	*I. lividus* F	*Ha. concinna* L	*Ha. concinna* N	*Ha. punctata* L	*I. arboricola* N	*Hyalomma* sp. N	*D. reticulatus* F
HS393	*ERI RUB*	2015.04.04		1													
HS394	*ERI RUB*	2015.04.04		1													
HS395	*TUR MER*	2015.04.04		4													
HS396	*TUR MER*	2015.04.04		1													
HS397	*TUR MER*	2015.04.04		2													
HS398	*ERI RUB*	2015.04.05		1													
HS399	*ERI RUB*	2015.04.05		1													
HS400	*TUR PHI*	2015.04.05		2													
HS401	*PRU MOD*	2015.04.05		2													
HS402	*ERI RUB*	2015.04.06		1													
HS403	*TUR MER*	2015.04.06		2													
HS404	*PRU MOD*	2015.04.06		1													
HS405	*ERI RUB*	2015.04.06		1													
HS406	*ERI RUB*	2015.04.06		1													
HS407	*TUR PHI*	2015.04.06		3													
HS408	*TUR PHI*	2015.04.06		1													
HS409	*TUR PHI*	2015.04.06		13													
HS410	*ERI RUB*	2015.04.06		1													
HS411	*COC COC*	2015.04.06		6													
HS412	*TRO TRO*	2015.04.06		1													
HS413	*ERI RUB*	2015.04.06		1													
HS414	*PRU MOD*	2015.04.06		3									1				
HS415	*ERI RUB*	2015.04.06		2													
HS416	*ERI RUB*	2015.04.07		2													
HS417	*ERI RUB*	2015.04.07		2													
HS418	*COC COC*	2015.04.07		1													
HS419	*PRU MOD*	2015.04.07		9													
HS420	*TUR MER*	2015.04.07		9													
HS421	*ERI RUB*	2015.04.07		2													
HS422	*ERI RUB*	2015.04.07	3	1													
HS423	*ERI RUB*	2015.04.07				1											
HS424	*PRU MOD*	2015.04.07		2													
HS425	*ERI RUB*	2015.04.08		1													
HS426	*TUR MER*	2015.04.08	1	5													
HS427	*ERI RUB*	2015.04.08		1													
HS428	*TUR MER*	2015.04.08		1													
HS429	*COC COC*	2015.04.08		5													
HS430	*GAR GLA*	2015.04.08		2													
HS431	*ERI RUB*	2015.04.09		1													
HS432	*ERI RUB*	2015.04.09		1													
HS433	*ERI RUB*	2015.04.09		1													
HS434	*TUR MER*	2015.04.09		2													
HS435	*TUR MER*	2015.04.09		5													
HS436	*TUR PHI*	2015.04.09		1													
HS437	*TUR MER*	2015.04.10	2	4													
HS438	*ERI RUB*	2015.04.10				1											
HS439	*ERI RUB*	2015.04.10		2													
HS440	*ERI RUB*	2015.04.10		3													
HS441	*ERI RUB*	2015.04.10		1													
HS442	*ERI RUB*	2015.04.10		1													
HS443	*ERI RUB*	2015.04.10		1													
HS445	*ERI RUB*	2015.04.10		2													
HS446	*ERI RUB*	2015.04.10		1													
HS447	*ERI RUB*	2015.04.10		1													
HS448	*ERI RUB*	2015.04.10		1													
HS449	*ERI RUB*	2015.04.10														1	
HS450	*ERI RUB*	2015.04.10		4													
HS451	*SYL ATR*	2015.04.11		1													

SAMPLE ID	HOST	DATE	*I. ricinus* L	*I. ricinus* N	*I. frontalis* L	*I. frontalis* N	*I. frontalis* F	*I. festai* F	*I. festai* M	*I. lividus* N	*I. lividus* F	*Ha. concinna* L	*Ha. concinna* N	*Ha. punctata* L	*I. arboricola* N	*Hyalomma* sp. N	*D. reticulatus* F
HS452	*TUR PHI*	2015.04.12		2													
HS453	*TUR MER*	2015.04.12		7													
HS454	*ERI RUB*	2015.04.12		1		1											
HS455	*PRU MOD*	2015.04.12		4													
HS456	*ERI RUB*	2015.04.12		2													
HS457	*PRU MOD*	2015.04.12		8													
HS458	*ERI RUB*	2015.04.12		1													
HS459	*ERI RUB*	2015.04.13		3													
HS460	*ERI RUB*	2015.04.13		1													
HS461	*TUR MER*	2015.04.13		3													
HS462	*ERI RUB*	2015.04.13		2													
HS463	*ERI RUB*	2015.04.13		1													
HS464	*TUR MER*	2015.04.13		13													
HS465	*TUR MER*	2015.04.13		13													
HS466	*PRU MOD*	2015.04.13		18													
HS467	*TUR PHI*	2015.04.13		1													
HS468	*ERI RUB*	2015.04.13		1													
HS469	*CAR CHL*	2015.04.13		1													
HS470	*PRU MOD*	2015.04.14		5													
HS471	*TUR PHI*	2015.04.14		3									1				
HS472	*TUR MER*	2015.04.14		1									1				
HS473	*ERI RUB*	2015.04.14		1													
HS474	*PRU MOD*	2015.04.14		1													
HS475	*TUR MER*	2015.04.14	1	7													
HS476	*PRU MOD*	2015.04.14		4													
FB1	*TUR MER*	2015.04.14		1													
FB2	*ERI RUB*	2015.04.14		2													
FB3	*ERI RUB*	2015.04.14		1													
FB4	*TUR PHI*	2015.04.14		1													
FB5	*TUR PHI*	2015.04.14		2													
FB6	*ERI RUB*	2015.04.14		3													
FB7	*TUR MER*	2015.04.14		7													
FB8	*ERI RUB*	2015.04.14	1	3													
FB9	*ERI RUB*	2015.04.14											1				
FB10	*COC COC*	2015.04.14	2	9													
FB11	*ERI RUB*	2015.04.14	1														
FB12	*TRO TRO*	2015.04.14		2													
FB13	*TUR MER*	2015.04.14		10													
FB14	*ERI RUB*	2015.04.14		1													
FB15	*TUR MER*	2015.04.15	1	8													
FB16	*TUR PHI*	2015.04.15	1	2													
FB17	*ERI RUB*	2015.04.15		1													
FB18	*ERI RUB*	2015.04.15		1													
FB19	*ERI RUB*	2015.04.15				1											
FB20	*PRU MOD*	2015.04.15		7													
FB21	*SYL ATR*	2015.04.15		1													
FB22	*ERI RUB*	2015.04.15				1											
FB23	*TUR MER*	2015.04.15		2													
FB24	*ERI RUB*	2015.04.16	1	2													
FB25	*TUR MER*	2015.04.16		4													
FB26	*ERI RUB*	2015.04.16		1													
FB27	*TUR MER*	2015.04.16		4													
FB28	*TUR MER*	2015.04.16	1	12													
FB29	*TRO TRO*	2015.04.17	4														
FB30	*TUR MER*	2015.04.17		4													
FB31	*ERI RUB*	2015.04.17		4													
FB32	*PRU MOD*	2015.04.17		1													
FB33	*TUR MER*	2015.04.17		7													

SAMPLE ID	HOST	DATE	*I. ricinus* L	*I. ricinus* N	*I. frontalis* L	*I. frontalis* N	*I. frontalis* F	*I. festai* F	*I. festai* M	*I. lividus* N	*I. lividus* F	*Ha. concinna* L	*Ha. concinna* N	*Ha. punctata* L	*I. arboricola* N	*Hyalomma* sp. N	*D. reticulatus* F
FB34	*GAR GLA*	2015.04.17	1	4													
FB35	*TUR MER*	2015.04.18		11													
FB37	*ERI RUB*	2015.04.18			1												
FB38	*ERI RUB*	2015.04.18			1								1				
FB39	*FIC ALB*	2015.04.18														1	
FB40	*COC COC*	2015.04.18		3													
FB41	*LUS MEG*	2015.04.23		1													
FB42	*ERI RUB*	2015.04.21											1				
FB43	*TUR MER*	2015.04.25										1	4				
FB44	*TUR MER*	2015.05.01		1													
FB45	*CAR CHL*	2015.05.03		1													
FB46	*TUR MER*	2015.05.03		2													
FB47	*SYL ATR*	2015.05.03		1													
FB48	*TUR MER*	2015.05.03		3													
FB49	*TUR MER*	2015.05.04		1													
FB50	*TUR MER*	2015.05.07		4													
FB51	*ACR ARU*	2015.05.08											1				
FB52	*TUR MER*	2015.05.18											1				
FB53	*ACR RIS*	2015.05.18		1													
FB54	*HIP ICT*	2015.05.24		1													
FB55	*ERI RUB*	2015.05.28		1													
FB56	*ACR RIS*	2015.05.28		2													
FB57	*CAR CHL*	2015.05.30		1													
FB58	*CAR CHL*	2015.05.30		1													
FB59	*CAR CHL*	2015.05.30		1													
FB60	*ACR RIS*	2015.06.01		1													
FB61	*ACR RIS*	2015.06.01		1													
FB62	*PAR MAJ*	2015.06.01		3													
FB63	*TUR MER*	2015.06.01		2													
FB64	*ACR SCI*	2015.06.10		1													
FB65	*SYL ATR*	2015.06.11	1	2													
FB66	*TUR PHI*	2015.06.11											1				
FB67	*LOC LUS*	2015.06.14										6	1				
FB68	*TUR PHI*	2015.06.17											1				
FB69	*LOC LUS*	2015.06.17		1								1	3				
FB70	*SYL ATR*	2015.06.17		1									1				
FB72	*ERI RUB*	2015.06.17		1								3					
FB73	*SIT EUR*	2015.06.17		2													
FB74	*TUR MER*	2015.06.17		1													
FB75	*TUR MER*	2015.06.17		4								1	1				
FB76	*RIP RIP*	2015.06.18								1							
FB77	*TUR PHI*	2015.06.20		1													
FB78	*LOC LUS*	2015.06.20										4	18				
FB83	*PAR MAJ*	2015.06.23		1													
FB84	*ACR SCI*	2015.06.24										1	1				
FB85	*LOC LUS*	2015.06.24										4	1				
FB86	*LOC LUS*	2015.06.24										2	1				
FB87	*TUR PHI*	2015.06.24		3								1	2				
FB88	*ACR SCI*	2015.07.04											1				
FB90	*LOC LUS*	2015.07.04											1				
FB91	*LOC LUS*	2015.07.04											2				
FB92	*ACR SCH*	2015.07.04											1				
FB95	*ACR SCI*	2015.07.04											1				
FB96	*ACR SCI*	2015.07.06											1				
FB97	*RIP RIP*	2015.07.07									3						
FB98	*LOC LUS*	2015.07.09											1				
FB99	*LUS MEG*	2015.07.09										3					
FB100	*ERI RUB*	2015.07.10										1					

SAMPLE ID	HOST	DATE	*I. ricinus* L	*I. ricinus* N	*I. frontalis* L	*I. frontalis* N	*I. frontalis* F	*I. festai* F	*I. festai* M	*I. lividus* N	*I. lividus* F	*Ha. concinna* L	*Ha. concinna* N	*Ha. punctata* L	*I. arboricola* N	*Hyalomma* sp. N	*D. reticulatus* F
FB101	*TUR MER*	2015.07.10	1														
FB102	*TUR MER*	2015.07.10		1									2				
FB103	*TUR PHI*	2015.07.10											1				
FB104	*LOC LUS*	2015.07.10											1				
FB106	*TUR MER*	2015.07.10											5				
FB107	*LOC LUS*	2015.07.10											1				
FB109	*TUR MER*	2015.07.10											2				
FB110	*ACR RIS*	2015.07.10		1													
FB111	*TUR PHI*	2015.07.10										2	1				
FB112	*TUR PHI*	2015.07.10		1									1				
FB113	*LOC LUS*	2015.07.10										2	2				
FB114	*ACR RIS*	2015.07.11	1														
FB115	*TUR PHI*	2015.07.11										6					
FB116	*TUR MER*	2015.07.11										10	5				
FB117	*TUR MER*	2015.07.11	2														
FB118	*TUR MER*	2015.07.11		1									1				
FB120	*ACR RIS*	2015.07.11											2				
FB121	*LOC LUS*	2015.07.11										2	4				
FB122	*ACR SCI*	2015.07.12	1									4					
FB123	*LOC LUS*	2015.07.12										1	3				
FB124	*LOC LUS*	2015.07.12											2				
FB125	*LOC LUS*	2015.07.12										1					
FB126	*TUR MER*	2015.07.12											1				
FB127	*ACR SCH*	2015.07.13											3				
FB128	*TUR PHI*	2015.07.13											1				
FB129	*ERI RUB*	2015.07.13	3														
FB130	*ACR RIS*	2015.07.13	1														
FB131	*ACR RIS*	2015.07.13	5	1								2	2				
FB132	*ERI RUB*	2015.07.13	6	2													
FB133	*LOC LUS*	2015.07.14	1									3	2				
FB134	*ACR RIS*	2015.07.14		1								15	5				
FB135	*LOC LUS*	2015.07.14										1	1				
FB136	*LOC LUS*	2015.07.14											2				
FB137	*ACR RIS*	2015.07.14	1														
FB138	*LOC LUS*	2015.07.14										1	1				
FB139	*ACR ARU*	2015.07.14											2				
FB140	*LOC LUS*	2015.07.14										1	1				
FB141	*ACR RIS*	2015.07.14										2					
FB142	*ACR SCH*	2015.07.15											2				
FB143	*LOC LUS*	2015.07.15											1				
FB144	*ERI RUB*	2015.07.15	2														
FB145	*LOC LUS*	2015.07.15											4				
FB146	*LOC LUS*	2015.07.15											4				
FB147	*TUR MER*	2015.07.15	1	2									1				
FB148	*TUR PHI*	2015.07.15		2								1					
FB149	*ACR SCI*	2015.07.15											3				
FB150	*ACR SCH*	2015.07.15										3	4				
FB151	*LOC LUS*	2015.07.15										1	5				
FB152	*ACR SCI*	2015.07.15		1													
FB153	*LOC LUS*	2015.07.15										1	3				
FB154	*ACR SCI*	2015.07.15										1					
FB155	*ACR SCI*	2015.07.15		1													
FB156	*LOC LUS*	2015.07.15											6				
FB157	*ACR SCI*	2015.07.15											1				
FB159	*TUR PHI*	2015.07.15		1													
FB160	*LOC LUS*	2015.07.15											4				
FB161	*TUR MER*	2015.07.15	1														
FB162	*TUR MER*	2015.07.15		1													

SAMPLE ID	HOST	DATE	*I. ricinus* L	*I. ricinus* N	*I. frontalis* L	*I. frontalis* N	*I. frontalis* F	*I. festai* F	*I. festai* M	*I. lividus* N	*I. lividus* F	*Ha. concinna* L	*Ha. concinna* N	*Ha. punctata* L	*I. arboricola* N	*Hyalomma* sp. N	*D. reticulatus* F
FB163	*LUS MEG*	2015.07.16	1	1													
FB164	*TUR MER*	2015.07.16											2				
FB165	*TUR MER*	2015.07.16	1									2	1				
FB166	*ERI RUB*	2015.07.16	3									1					
FB167	*ACR SCI*	2015.07.16										6	1				
FB168	*ACR SCI*	2015.07.16										1					
FB169	*LOC LUS*	2015.07.16										1					
FB170	*TUR MER*	2015.07.16		2									2				
FB171	*TUR PHI*	2015.07.16	4										2				
FB172	*ACR SCI*	2015.07.16											2				
FB173	*TUR MER*	2015.07.16											1				
FB174	*LOC LUS*	2015.07.16											2				
FB175	*TUR MER*	2015.07.16										1	2				
FB176	*ACR SCI*	2015.07.16										4					
FB177	*TUR MER*	2015.07.16		1									2				
FB178	*ACR ARU*	2015.07.17		3									1				
FB179	*ACR SCI*	2015.07.17		1													
FB180	*LOC LUS*	2015.07.17											4				
FB185	*ACR SCI*	2015.07.17											1				
FB190	*ACR SCI*	2015.07.18		1													
FB191	*ERI RUB*	2015.07.19	1	1													
FB192	*ACR RIS*	2015.07.19		1													
FB193	*ERI RUB*	2015.07.19	3														
FB194	*ACR RIS*	2015.07.19		1													
FB195	*ACR RIS*	2015.07.19											1				
FB196	*LOC LUS*	2015.07.19											2				
FB197	*TUR PHI*	2015.07.20											1				
FB198	*LOC FLU*	2015.07.20											2				
FB200	*PHY COL*	2015.07.20	4	1													
FB201	*COT COT*	2015.07.20												28			
FB202	*LOC LUS*	2015.07.20										8	4				
FB203	*ACR ARU*	2015.07.21											1				
FB204	*LUS MEG*	2015.07.21	1														
FB205	*TUR MER*	2015.07.21	1	4													
FB206	*ERI RUB*	2015.07.21	2														
FB207	*LUS MEG*	2015.07.22		1													
FB208	*ACR RIS*	2015.07.22	1														
FB209	*ERI RUB*	2015.07.23	1														
FB210	*ACR SCI*	2015.07.23		3									1				
FB211	*ACR RIS*	2015.07.23	2														
FB212	*ACR RIS*	2015.07.23	1														
FB213	*ACR RIS*	2015.07.23		1													
FB214	*ACR ARU*	2015.07.23		2													
FB215	*ACR RIS*	2015.07.23		1													
FB216	*LOC LUS*	2015.07.24										1	2				
FB217	*ACR SCH*	2015.07.25										1	4				
FB218	*ACR RIS*	2015.07.25		1													
FB219	*ERI RUB*	2015.07.25											1				
FB220	*TUR MER*	2015.07.25											1				
FB221	*LOC LUS*	2015.07.25										1	1				
FB222	*LOC LUS*	2015.07.25											1				
FB223	*LOC LUS*	2015.07.24											5				
FB224	*LOC LUS*	2015.07.24											3				
FB225	*LOC LUS*	2015.07.24										1	1				
FB226	*TUR PHI*	2015.07.27	1														
FB227	*LOC LUS*	2015.07.27										7	3				
FB228	*TUR MER*	2015.07.27	31	2													
FB229	*ACR RIS*	2015.07.27	1														

SAMPLE ID	HOST	DATE	*I. ricinus* L	*I. ricinus* N	*I. frontalis* L	*I. frontalis* N	*I. frontalis* F	*I. festai* F	*I. festai* M	*I. lividus* N	*I. lividus* F	*Ha. concinna* L	*Ha. concinna* N	*Ha. punctata* L	*I. arboricola* N	*Hyalomma* sp. N	*D. reticulatus* F
FB230	*ERI RUB*	2015.07.27		1													
FB231	*LOC LUS*	2015.07.27											1				
FB232	*ERI RUB*	2015.07.27	3														
FB233	*ACR SCI*	2015.07.27											1				
FB234	*LOC LUS*	2015.07.27										9	15				
FB235	*ACR SCI*	2015.07.27											1				
FB236	*LOC LUS*	2015.07.27											1				
FB237	*LOC LUS*	2015.07.27	1									3	1				
FB238	*LOC LUS*	2015.07.27											5				
FB240	*LOC LUS*	2015.07.27											2				
FB241	*LOC LUS*	2015.07.27											3				
FB242	*ACR RIS*	2015.07.28	1	1									2				
FB243	*TUR PHI*	2015.07.28	3										1				
FB246	*ACR RIS*	2015.07.28	1														
FB247	*ACR SCH*	2015.07.28											1				
FB248	*ACR SCI*	2015.07.28											2				
FB249	*TUR MER*	2015.07.28		1													
FB250	*LUS MEG*	2015.07.29	1														
FB251	*ACR SCI*	2015.07.29	1														
FB252	*LOC LUS*	2015.07.29										1	7				
FB253	*LOC LUS*	2015.07.29											7				
FB254	*ACR SCI*	2015.07.30	1														
FB255	*ACR RIS*	2015.07.30											1				
FB256	*TUR PHI*	2015.07.31											1				
FB257	*TUR PHI*	2015.07.31	2														
FB258	*COT COT*	2015.07.31	1														
FB259	*ACR ARU*	2015.07.31	1														
FB260	*LOC LUS*	2015.07.31											1				
FB261	*LOC LUS*	2015.07.31											1				
FB262	*LOC LUS*	2015.07.31										1	5				
FB263	*ACR SCI*	2015.08.01		1													
FB264	*LOC LUS*	2015.08.01										5	14				
FB265	*ACR SCH*	2015.08.01											2				
FB266	*ACR SCI*	2015.08.02	1														
FB267	*ACR RIS*	2015.08.02										4					
FB268	*ACR RIS*	2015.08.02											1				
FB269	*ACR RIS*	2015.08.02		1													
FB270	*LOC LUS*	2015.08.02										2	5				
FB271	*LOC LUS*	2015.08.02										15	3				
FB272	*ACR SCH*	2015.08.02											1				
FB273	*ACR RIS*	2015.08.03	2	2													
FB274	*LOC LUS*	2015.08.03										11	4				
FB275	*ACR RIS*	2015.08.03	2														
FB276	*ACR RIS*	2015.08.04		1													
FB278	*TUR MER*	2015.08.05	2														
FB279	*ACR RIS*	2015.08.05		2													
FB280	*ACR SCI*	2015.08.05		1													
FB281	*TUR PHI*	2015.08.06											1				
FB282	*ACR ARU*	2015.08.06		1													
FB284	*LUS MEG*	2015.08.07	2														
FB285	*LOC LUS*	2015.08.08											7				
FB286	*ACR RIS*	2015.08.08	1	1													
FB287	*LOC LUS*	2015.08.08										4	1				
FB288	*LUS LUS*	2015.08.09	1														
FB289	*ACR SCI*	2015.08.09	2														
FB290	*LOC LUS*	2015.08.09											3				
FB291	*LOC LUS*	2015.08.09										1	1				
FB292	*ACR ARU*	2015.08.11											1				

SAMPLE ID	HOST	DATE	*I. ricinus* L	*I. ricinus* N	*I. frontalis* L	*I. frontalis* N	*I. frontalis* F	*I. festai* F	*I. festai* M	*I. lividus* N	*I. lividus* F	*Ha. concinna* L	*Ha. concinna* N	*Ha. punctata* L	*I. arboricola* N	*Hyalomma* sp. N	*D. reticulatus* F
FB293	*LUS MEG*	2015.08.12										2	1				
FB294	*ACR SCI*	2015.08.12		1													
FB295	*ACR SCI*	2015.08.12											1				
FB296	*LUS MEG*	2015.08.13	1														
FB297	*LUS LUS*	2015.08.13	5														
FB298	*LUS MEG*	2015.08.13	3	1													
FB299	*LUS LUS*	2015.08.13	3	1													
FB300	*LOC LUS*	2015.08.19											1				
FB301	*ACR RIS*	2015.08.21		2													
FB302	*ERI RUB*	2015.08.18	1														
FB303	*LUS LUS*	2015.08.22	3														
FB304	*TUR MER*	2015.08.22										1					
FB305	*LUS LUS*	2015.08.22	7	1													
FB306	*LUS LUS*	2015.08.22	4														
FB307	*ERI RUB*	2015.08.22	1														
FB308	*SYL COM*	2015.08.22	1	1													
FB309	*SYL ATR*	2015.08.22		1													
FB310	*SYL ATR*	2015.08.22		1													
FB311	*SYL ATR*	2015.08.22	3														
FB312	*LOC FLU*	2015.08.23										6					
FB313	*SYL ATR*	2015.08.23	1														
FB315	*LOC LUS*	2015.08.23											2				
FB316	*ACR RIS*	2015.08.24		1													
FB318	*ACR RIS*	2015.08.25		1													
FB319	*TUR PHI*	2015.08.25	1									2	1				
FB320	*SYL COM*	2015.08.25	1														
FB321	*ERI RUB*	2015.08.25	1	1													
FB323	*ACR SCH*	2015.08.26											1				
FB324	*SYL ATR*	2015.08.26	1														
FB325	*SYL ATR*	2015.08.27	2														
FB326	*TRO TRO*	2015.08.27	1														
FB327	*SYL ATR*	2015.08.27	1														
FB328	*LUS LUS*	2015.08.27	8														
FB329	*LOC FLU*	2015.08.27										8					
FB330	*SYL ATR*	2015.08.27	1														
FB331	*TUR MER*	2015.08.27	1	1													
FB332	*ACR SCI*	2015.08.27		1													
FB333	*ACR RIS*	2015.08.27		1													
FB334	*LUS MEG*	2015.08.28	2														
FB335	*SYL ATR*	2015.08.28		1													
FB336	*ERI RUB*	2015.08.28		1													
FB337	*SYL COM*	2015.08.28		1													
FB338	*ERI RUB*	2015.08.29	1	1													
FB339	*ERI RUB*	2015.08.29	1														
FB340	*SYL ATR*	2015.08.29	1														
FB341	*ERI RUB*	2015.08.29	3														
FB342	*LUS LUS*	2015.08.30	1														
FB343	*SYL ATR*	2015.08.30	2														
FB344	*SYL ATR*	2015.08.30	1														
FB345	*SYL ATR*	2015.08.30	2														
FB346	*TRO TRO*	2015.08.30	2														
FB347	*ANT TRI*	2015.08.31	1	1													
FB348	*ANT TRI*	2015.08.31		2													
FB349	*ACR SCI*	2015.08.31		1													
FB350	*ERI RUB*	2015.09.01	2														
FB351	*SYL BOR*	2015.09.01		1													
FB352	*SYL ATR*	2015.09.02	1														
FB353	*ACR SCI*	2015.09.08		1													

SAMPLE ID	HOST	DATE	*I. ricinus* L	*I. ricinus* N	*I. frontalis* L	*I. frontalis* N	*I. frontalis* F	*I. festai* F	*I. festai* M	*I. lividus* N	*I. lividus* F	*Ha. concinna* L	*Ha. concinna* N	*Ha. punctata* L	*I. arboricola* N	*Hyalomma* sp. N	*D. reticulatus* F
FB354	*ANT TRI*	2015.09.08	1														
FB355	*ANT TRI*	2015.09.08		2													
FB356	*ERI RUB*	2015.09.10	1	1													
FB357	*SYL COM*	2015.09.10		1													
FB359	*ANT TRI*	2015.09.10		1													
FB360	*ANT TRI*	2015.09.12	1	1													
FB361	*ERI RUB*	2015.09.13	1														
FB362	*SYL COM*	2015.09.13		1													
FB363	*ERI RUB*	2015.09.14	2														
FB364	*SYL BOR*	2015.09.14	1	1													
FB365	*SYL ATR*	2015.09.14	1														
FB366	*SYL ATR*	2015.09.14	1														
FB367	*LUS LUS*	2015.09.15		1													
FB368	*SYL ATR*	2015.09.15	2														
FB369	*SYL ATR*	2015.09.15		1													
FB370	*PHY COL*	2015.09.15	1														
FB371	*ERI RUB*	2015.09.15										1					
FB372	*PHY TRO*	2015.09.17	1														
FB373	*SYL COM*	2015.09.17		1													
FB374	*SYL ATR*	2015.09.17		2													
FB375	*ERI RUB*	2015.09.17	1														
FB376	*ERI RUB*	2015.09.19	4														
FB377	*ERI RUB*	2015.09.19	2														
FB378	*ERI RUB*	2015.09.19										1					
FB379	*SYL ATR*	2015.09.19		1													
FB380	*PHO PHO*	2015.09.22		4													
FB381	*ERI RUB*	2015.09.24		1													
FB382	*ERI RUB*	2015.09.28	1														
FB383	*ERI RUB*	2015.09.28	1														
FB384	*ACR SCH*	2015.09.28										2					
FB385	*ERI RUB*	2015.09.28	1														
FB386	*ERI RUB*	2015.09.28	1	2													
FB387	*ERI RUB*	2015.09.29		1													
FB388	*ERI RUB*	2015.10.01	1														
FB389	*PRU MOD*	2015.10.01		2								1					
FB390	*SYL ATR*	2015.10.01		1													
FB391	*TUR PHI*	2015.10.01		1													
FB392	*ERI RUB*	2015.10.02	1	2													
FB393	*PRU MOD*	2015.10.02		2													
FB394	*PRU MOD*	2015.10.04		2								1					
FB395	*TUR MER*	2015.10.04		6								1					
FB396	*PAR MAJ*	2015.10.04		1													
FB397	*ACR SCI*	2015.10.05	1														
FB398	*SYL ATR*	2015.10.05		2													
FB399	*ERI RUB*	2015.10.06		2													
FB400	*FRI COE*	2015.10.06		1													
FB401	*PHY COL*	2015.10.08	2														
FB402	*TUR PHI*	2015.10.08		1													
FB403	*ERI RUB*	2015.10.08		1													
FB404	*ERI RUB*	2015.10.08		1													
FB405	*TUR MER*	2015.10.13		1													
FB406	*TUR MER*	2015.10.13		3													
FB408	*ERI RUB*	2015.10.18		1													
FB409	*ERI RUB*	2015.10.18		2													
FB410	*ERI RUB*	2015.10.18	1														
FB411	*PHY COL*	2015.10.18	1	1													
FB412	*PRU MOD*	2015.10.18		3													
FB413	*ERI RUB*	2015.10.18		1													

SAMPLE ID	HOST	DATE	*I. ricinus* L	*I. ricinus* N	*I. frontalis* L	*I. frontalis* N	*I. frontalis* F	*I. festai* F	*I. festai* M	*I. lividus* N	*I. lividus* F	*Ha. concinna* L	*Ha. concinna* N	*Ha. punctata* L	*I. arboricola* N	*Hyalomma* sp. N	*D. reticulatus* F
FB414	*ERI RUB*	2015.10.18	1														
FB415	*ERI RUB*	2015.10.19	2														
FB416	*ERI RUB*	2015.10.19	1														
FB417	*TUR PHI*	2015.10.21		1													
FB418	*ERI RUB*	2015.10.22		2													
FB419	*TRO TRO*	2015.10.22	1														
FB420	*ERI RUB*	2015.10.23		1													
FB422	*TUR MER*	2015.10.23		2													
FB423	*ERI RUB*	2015.10.24	1														
FB425	*TUR MER*	2015.10.25		1													
FB426	*TRO TRO*	2015.10.26		1													
FB427	*ERI RUB*	2015.10.27	6														
FB428	*ERI RUB*	2015.10.27	2														
FB429	*ERI RUB*	2015.10.27		1													
FB430	*TUR MER*	2015.10.28		5													
FB431	*TUR MER*	2015.10.28		1													
FB432	*PHY COL*	2015.10.28		1													
FB433	*TUR MER*	2015.10.28	2	6													
FB435	*TUR MER*	2015.10.29		1													
FB436	*TUR MER*	2015.10.29		2													
FB437	*TUR MER*	2015.10.29		1													
FB438	*ERI RUB*	2015.10.29		1													
FB440	*ERI RUB*	2015.10.31		1													
FB441	*ERI RUB*	2015.10.31		1													
FB442	*TUR MER*	2015.11.04		1													
FB443	*TUR MER*	2015.11.21	1														
FB445	*CAR CHL*	2015.12.12					1										
FB446	*TUR MER*	2015.12.27		1													
FB447	*TUR MER*	2016.02.20		1													
FB448	*TUR MER*	2016.03.10		3													
FB449	*TUR MER*	2016.03.11		1													
FB450	*TUR PHI*	2016.03.11		1													
FB451	*TUR MER*	2016.03.11		1													
FB452	*TUR MER*	2016.03.12		2													
FB453	*TUR PHI*	2016.03.12		1													
FB454	*TUR MER*	2016.03.12		5													
FB455	*TUR MER*	2016.03.12		2													
FB456	*TUR MER*	2016.03.12		1													
FB457	*TUR MER*	2016.03.12		3													
FB458	*TUR MER*	2016.03.12		2													
FB459	*TUR MER*	2016.03.12		2													
FB460	*TUR MER*	2016.03.13		2													
FB461	*TUR PHI*	2016.03.13				1											
FB462	*TUR MER*	2016.03.14		13													
FB463	*TUR MER*	2016.03.15		7													
FB464	*TUR MER*	2016.03.15		2													
FB465	*TUR MER*	2016.03.16		7													
FB466	*TUR PHI*	2016.03.16		1													
FB467	*TUR MER*	2016.03.16		1													
FB468	*TUR MER*	2016.03.16		3													
FB469	*TUR PHI*	2016.03.17		1													
FB470	*REG REG*	2016.03.17		1													
FB471	*PRU MOD*	2016.03.17		1													
FB472	*ERI RUB*	2016.03.17		2													
FB473	*TUR MER*	2016.03.18		1													
FB474	*ERI RUB*	2016.03.18		2													
FB475	*PRU MOD*	2016.03.18		1													
FB476	*PRU MOD*	2016.03.19		2				1									

SAMPLE ID	HOST	DATE	*I. ricinus* L	*I. ricinus* N	*I. frontalis* L	*I. frontalis* N	*I. frontalis* F	*I. festai* F	*I. festai* M	*I. lividus* N	*I. lividus* F	*Ha. concinna* L	*Ha. concinna* N	*Ha. punctata* L	*I. arboricola* N	*Hyalomma* sp. N	*D. reticulatus* F
FB477	*TUR MER*	2016.03.19		2													
FB478	*TUR MER*	2016.03.19		1													
FB479	*PHY COL*	2016.03.19		1													
FB480	*ERI RUB*	2016.03.19				1											
FB481	*ERI RUB*	2016.03.19		1													
FB482	*PRU MOD*	2016.03.20		3													
FB483	*ERI RUB*	2016.03.20		1													
FB484	*PRU MOD*	2016.03.20		1													
FB485	*TUR MER*	2016.03.20		3													
FB486	*PRU MOD*	2016.03.20		1													
FB487	*TUR MER*	2016.03.20		1													
FB488	*PRU MOD*	2016.03.20		1													
FB490	*ERI RUB*	2016.03.21	2	2													
FB491	*ERI RUB*	2016.03.21		1													
FB492	*PRU MOD*	2016.03.21		1													
FB493	*ERI RUB*	2016.03.21				1											
FB494	*TUR PHI*	2016.03.21		1													
FB495	*PRU MOD*	2016.03.21		1													
FB496	*TUR PHI*	2016.03.21		1													
FB497	*PAR MAJ*	2016.03.21				1											
FB498	*TUR MER*	2016.03.21		1													
FB499	*TUR MER*	2016.03.22		6													
FB500	*TUR MER*	2016.03.22		11													
FB501	*PRU MOD*	2016.03.22		1													
FB502	*TUR MER*	2016.03.22	1	6													
FB503	*ERI RUB*	2016.03.23	1														
FB504	*TUR MER*	2016.03.23		1													
FB505	*TUR PHI*	2016.03.23		1													
FB506	*PRU MOD*	2016.03.23		3													
FB507	*PRU MOD*	2016.03.23		4													
FB508	*ERI RUB*	2016.03.23		1													
FB509	*ERI RUB*	2016.03.24		1													
FB510	*TUR MER*	2016.03.24		2													
FB511	*ERI RUB*	2016.03.25		1													
FB512	*TUR MER*	2016.03.25		6													
FB513	*CAR CHL*	2016.03.25		1													
FB514	*REM PEN*	2016.03.25					1										
FB515	*TUR MER*	2016.03.27		1													
FB516	*ERI RUB*	2016.03.27		2													
FB517	*PRU MOD*	2016.03.27		1													
FB518	*TUR MER*	2016.03.27		2													
FB519	*PRU MOD*	2016.03.27		2													
FB520	*TRO TRO*	2016.03.27		1													
FB521	*PRU MOD*	2016.03.27		6													
FB522	*TUR PHI*	2016.03.27				1											
FB523	*ERI RUB*	2016.03.27		1													
FB524	*PRU MOD*	2016.03.27		1													
FB525	*PRU MOD*	2016.03.27		7													
FB526	*ERI RUB*	2016.03.27		1													
FB527	*ERI RUB*	2016.03.27		1													
FB528	*ERI RUB*	2016.03.27		2													
FB529	*ERI RUB*	2016.03.27		1													
FB530	*ERI RUB*	2016.03.27		1													
FB531	*PRU MOD*	2016.03.27		3													
FB532	*TUR PHI*	2016.03.27		1													
FB533	*ERI RUB*	2016.03.28		1													
FB534	*ERI RUB*	2016.03.28		4													
FB535	*ERI RUB*	2016.03.28		2													

SAMPLE ID	HOST	DATE	*I. ricinus* L	*I. ricinus* N	*I. frontalis* L	*I. frontalis* N	*I. frontalis* F	*I. festai* F	*I. festai* M	*I. lividus* N	*I. lividus* F	*Ha. concinna* L	*Ha. concinna* N	*Ha. punctata* L	*I. arboricola* N	*Hyalomma* sp. N	*D. reticulatus* F
FB536	*PRU MOD*	2016.03.28	1	9													
FB537	*PRU MOD*	2016.03.28		7													
FB538	*ERI RUB*	2016.03.28		1													
FB539	*PRU MOD*	2016.03.28		3													
FB540	*ERI RUB*	2016.03.28		1													
FB541	*ERI RUB*	2016.03.30		1													
FB542	*PRU MOD*	2016.03.30		3													
FB543	*TUR PHI*	2016.03.30		3													
FB544	*TUR PHI*	2016.03.30		3													
FB545	*PRU MOD*	2016.03.30		4													
FB546	*PRU MOD*	2016.03.30		4													
FB547	*PRU MOD*	2016.03.30		4													
FB548	*PRU MOD*	2016.03.30		9													
FB549	*PRU MOD*	2016.03.30		3													
FB550	*ERI RUB*	2016.03.30				1											
FB551	*TUR MER*	2016.03.30		1													
FB552	*PRU MOD*	2016.03.30		4													
FB553	*PRU MOD*	2016.03.30		1													
FB554	*PRU MOD*	2016.03.30		3													
FB555	*TUR MER*	2016.03.30		5													
FB556	*PRU MOD*	2016.03.30		2													
FB557	*CAR CHL*	2016.03.30		2													
FB558	*PRU MOD*	2016.03.30		1													
FB559	*ERI RUB*	2016.03.30		7													
FB560	*PRU MOD*	2016.03.30		3													
FB561	*PRU MOD*	2016.03.31		2													
FB562	*PRU MOD*	2016.03.31		1													
FB563	*PRU MOD*	2016.03.31		6													
FB564	*ERI RUB*	2016.03.31		2													
FB565	*ERI RUB*	2016.03.31		1													
FB566	*PRU MOD*	2016.03.31		3													
FB567	*PRU MOD*	2016.03.31		6													
FB568	*PRU MOD*	2016.03.31		5													
FB569	*TUR MER*	2016.04.02		1													
FB570	*ERI RUB*	2016.04.02		1													
FB571	*PRU MOD*	2016.04.02		3													
FB572	*ERI RUB*	2016.04.02	5	11													
FB573	*SYL ATR*	2016.04.02		1													
FB574	*ERI RUB*	2016.04.02		1													
FB575	*ERI RUB*	2016.04.03		1													
FB576	*TUR PHI*	2016.04.03		2													
FB577	*ERI RUB*	2016.04.04		1													
FB578	*PHY COL*	2016.04.04		1													
FB579	*ERI RUB*	2016.04.06		1													
FB580	*CAR CHL*	2016.04.06		2													
FB581	*TUR PHI*	2016.04.07		1												1	
FB582	*TUR MER*	2016.04.07		1													
FB583	*ERI RUB*	2016.04.07	2														
FB584	*TUR MER*	2016.04.07		3													
FB585	*ERI RUB*	2016.04.07		1													
FB586	*ERI RUB*	2016.04.08	1	3													
FB587	*GAR GLA*	2016.04.08		1													
FB588	*ERI RUB*	2016.04.08		1													
FB589	*TUR MER*	2016.04.09		1													
FB590	*TUR PHI*	2016.04.09		1													
FB591	*TUR MER*	2016.04.10		4													
FB592	*TUR MER*	2016.04.10		2													
FB593	*TUR MER*	2016.04.10		3													

SAMPLE ID	HOST	DATE	*I. ricinus* L	*I. ricinus* N	*I. frontalis* L	*I. frontalis* N	*I. frontalis* F	*I. festai* F	*I. festai* M	*I. lividus* N	*I. lividus* F	*Ha. concinna* L	*Ha. concinna* N	*Ha. punctata* L	*I. arboricola* N	*Hyalomma* sp. N	*D. reticulatus* F
FB594	*ERI RUB*	2016.04.13	1														
FB595	*TUR MER*	2016.04.13	4	10								2	3				
FB596	*TUR MER*	2016.04.13		1													
FB597	*TUR MER*	2016.04.14		1													
FB598	*TUR MER*	2016.04.15		2													
FB599	*ACR SCI*	2016.04.15		1													
FB600	*ERI RUB*	2016.04.15		2													
FB601	*ERI RUB*	2016.04.15		1													
FB602	*TUR MER*	2016.04.16		2													
FB603	*TUR MER*	2016.04.16		5													
FB604	*ERI RUB*	2016.04.16		1													
FB605	*TUR MER*	2016.04.23		7													
FB606	*TUR MER*	2016.04.23		2													
FB607	*ACR SCI*	2016.05.05		1													
FB608	*TUR MER*	2016.05.07		18													
FB609	*TUR MER*	2016.05.09		3													
FB610	*EMB CIT*	2016.05.11											2				
FB611	*TUR MER*	2016.05.12		1													
FB612	*TUR MER*	2016.05.14		7													
FB613	*TUR MER*	2016.05.14		3													
FB614	*EMB SCH*	2016.05.14		1													
FB615	*TUR PHI*	2016.05.18		4									1				
FB616	*TUR MER*	2016.05.18		10													
FB617	*TUR MER*	2016.05.18		2													
FB618	*ERI RUB*	2016.05.21		4													
FB619	*TUR PHI*	2016.05.21		3													
FB620	*ACR SCH*	2016.05.24		2													
FB621	*CAR CHL*	2016.05.25	1	5													
FB622	*LOC LUS*	2016.05.28											1				
FB623	*FRI COE*	2016.05.28		4													
FB624	*TUR MER*	2016.05.28		2													
FB625	*PHY COL*	2016.06.01		3													
FB626	*PHY COL*	2016.06.01		5								1					
FB627	*CER BRA*	2016.06.01		1													
FB628	*TUR MER*	2016.06.02		5													
FB629	*SYL ATR*	2016.06.02		1													
FB630	*EMB SCH*	2016.06.03											5				
FB631	*ACR SCI*	2016.06.03		1													
FB632	*LOC LUS*	2016.06.03											1				
FB633	*CAR CHL*	2016.06.03		1													
FB634	*LOC LUS*	2016.06.04											2				
FB635	*SYL ATR*	2016.06.04		1													
FB637	*ERI RUB*	2016.06.08										2					
FB638	*SYL ATR*	2016.06.08		2													
FB639	*SYL ATR*	2016.06.08		1													
FB640	*SYL ATR*	2016.06.10		1								1					
FB641	*PAR MAJ*	2016.06.10		1													
FB642	*SYL ATR*	2016.06.10		1													
FB643	*SYL ATR*	2016.06.10		1													
FB644	*FRI COE*	2016.06.11		1													
FB646	*ACR SCI*	2016.06.11		1													
FB647	*SYL ATR*	2016.06.14		1													
FB648	*ERI RUB*	2016.06.14										1					
FB649	*TUR PHI*	2016.06.14		2													
FB650	*LUS MEG*	2016.06.18		3													
FB651	*ERI RUB*	2016.06.18		2								1					
FB652	*TUR PHI*	2016.06.18		3									5				
FB653	*TUR PHI*	2016.06.25											1				

SAMPLE ID	HOST	DATE	*I. ricinus* L	*I. ricinus* N	*I. frontalis* L	*I. frontalis* N	*I. frontalis* F	*I. festai* F	*I. festai* M	*I. lividus* N	*I. lividus* F	*Ha. concinna* L	*Ha. concinna* N	*Ha. punctata* L	*I. arboricola* N	*Hyalomma* sp. N	*D. reticulatus* F
FB654	*TUR MER*	2016.07.02		1													
FB655	*TUR MER*	2016.07.02										1	2				
FB657	*ACR SCI*	2016.07.02		1													
FB658	*ACR SCI*	2016.07.02											1				
FB659	*PAR MAJ*	2016.07.02		2													
FB660	*LOC LUS*	2016.07.02										1	1				
FB661	*ACR SCI*	2016.07.02		1								1	1				
FB662	*LOC LUS*	2016.07.02											3				
FB663	*LOC LUS*	2016.07.02											2				
FB664	*LOC LUS*	2016.07.02											1				
FB665	*LOC LUS*	2016.07.02											1				
FB666	*LUS MEG*	2016.07.10	1									2					
FB668	*SYL ATR*	2016.07.10	1														
FB669	*ERI RUB*	2016.07.10	4														
FB670	*SYL ATR*	2016.07.10		1													
FB672	*SYL ATR*	2016.07.10		2													
FB673	*ACR SCH*	2016.07.10										7	5				
FB674	*LOC LUS*	2016.07.10										1	2				
FB675	*ACR SCI*	2016.07.10										5	1				
FB676	*TUR MER*	2016.07.10		1									1				
FB677	*ACR RIS*	2016.07.10		1													
FB678	*LOC LUS*	2016.07.10											5				
FB679	*ACR SCI*	2016.07.10											2				
FB680	*PHY COL*	2016.07.10	1														
FB682	*ACR RIS*	2016.07.10		1													
FB683	*LUS MEG*	2016.07.10	1														
FB684	*LOC LUS*	2016.07.10											3				
FB685	*ACR RIS*	2016.07.11		1													
FB686	*TUR PHI*	2016.07.11		2									2				
FB687	*SYL NIS*	2016.07.11		1									1				
FB689	*ACR RIS*	2016.07.11		1													
FB690	*TUR PHI*	2016.07.11		5									4				
FB691	*ACR SCH*	2016.07.12										4	1				
FB692	*LOC FLU*	2016.07.12										27	3				
FB693	*LOC LUS*	2016.07.12										2	4				
FB694	*ACR RIS*	2016.07.12		1													
FB695	*TUR PHI*	2016.07.12		1													
FB696	*ACR SCI*	2016.07.12		1													
FB697	*ERI RUB*	2016.07.13	1														
FB698	*ACR SCI*	2016.07.15											1				
FB699	*ACR SCI*	2016.07.15											1				
FB700	*LOC LUS*	2016.07.15										1					
FB701	*ACR SCH*	2016.07.15											1				
FB702	*ACR SCH*	2016.07.15											2				
FB703	*LOC LUS*	2016.07.15											2				
FB704	*ACR SCI*	2016.07.24											1				
FB705	*LOC LUS*	2016.07.25											3				
FB706	*ERI RUB*	2016.07.25	3														
FB707	*SYL ATR*	2016.07.25	2														
FB708	*TRO TRO*	2016.07.25	1														
FB709	*LOC LUS*	2016.07.25	2														
FB710	*ACR RIS*	2016.07.25		1									1				
FB711	*ACR RIS*	2016.07.25		1													
FB712	*ACR RIS*	2016.07.25											3				
FB713	*LOC LUS*	2016.07.27											4				
FB714	*ACR RIS*	2016.07.27		1													
FB715	*TUR PHI*	2016.07.27										2	3				
FB716	*ERI RUB*	2016.07.27	1														

SAMPLE ID	HOST	DATE	*I. ricinus* L	*I. ricinus* N	*I. frontalis* L	*I. frontalis* N	*I. frontalis* F	*I. festai* F	*I. festai* M	*I. lividus* N	*I. lividus* F	*Ha. concinna* L	*Ha. concinna* N	*Ha. punctata* L	*I. arboricola* N	*Hyalomma* sp. N	*D. reticulatus* F
FB717	*ERI RUB*	2016.07.27	5														
FB718	*ACR SCI*	2016.07.27	2	1													
FB719	*TUR PHI*	2016.07.27											4				
FB720	*ACR SCI*	2016.07.27		2								1					
FB721	*ACR MEL*	2016.07.28											1				
FB722	*LOC LUS*	2016.07.28											1				
FB723	*SYL CUR*	2016.07.28		1													
FB724	*LOC LUS*	2016.07.28											1				
FB725	*LOC LUS*	2016.07.28											2				
FB726	*ACR SCI*	2016.07.28										1					
FB727	*ACR RIS*	2016.07.29	1	1													
FB728	*TUR MER*	2016.07.30		1													
FB729	*SYL ATR*	2016.07.30	2														
FB730	*TUR MER*	2016.07.30		1													
FB731	*ACR SCI*	2016.07.30											1				
FB732	*SYL ATR*	2016.07.30	8														
FB733	*SYL BOR*	2016.07.31											1				
FB734	*ACR ARU*	2016.07.31		1													
FB735	*SYL ATR*	2016.07.31	3														
FB736	*LUS MEG*	2016.08.01	1														
FB737	*TUR PHI*	2016.08.01		1													
FB738	*TUR PHI*	2016.08.02		1									1				
FB739	*SYL ATR*	2016.08.02											1				
FB740	*ACR RIS*	2016.08.02	1	1													
FB741	*ACR RIS*	2016.08.02	1														
FB742	*ACR RIS*	2016.08.02											1				
FB743	*ACR RIS*	2016.08.02	1														
FB744	*ACR RIS*	2016.08.02	1														
FB745	*LOC LUS*	2016.08.02											1				
FB746	*ACR ARU*	2016.08.02											1				
FB747	*ACR ARU*	2016.08.02											1				
FB748	*LOC LUS*	2016.08.02										1					
FB749	*SYL ATR*	2016.08.03	1														
FB750	*ACR SCI*	2016.08.03		1													
FB751	*ACR SCI*	2016.08.04	1	1									1				
FB752	*ACR RIS*	2016.08.04	4										2				
FB753	*LUS MEG*	2016.08.04	1														
FB754	*SYL ATR*	2016.08.04	1														
FB755	*SYL ATR*	2016.08.04	3														
FB756	*ACR ARU*	2016.08.04	1														
FB757	*SYL COM*	2016.08.05		1													
FB758	*LUS MEG*	2016.08.05	2									1	2				
FB759	*ACR SCH*	2016.08.05											1				
FB760	*ACR SCI*	2016.08.05		1													
FB761	*LOC LUS*	2016.08.05											1				
FB762	*SYL ATR*	2016.08.05											1				
FB763	*SYL ATR*	2016.08.05											1				
FB764	*ACR ARU*	2016.08.05										1					
FB765	*ACR SCI*	2016.08.06	1														
FB766	*SYL COM*	2016.08.07										1					
FB767	*ACR RIS*	2016.08.07	1														
FB768	*ACR ARU*	2016.08.07		1													
FB769	*LOC LUS*	2016.08.07											1				
FB770	*ACR RIS*	2016.08.07	1														
FB771	*ACR RIS*	2016.08.07	1	1													
FB773	*LOC LUS*	2016.08.08											2				
FB775	*LOC LUS*	2016.08.09										2	8				
FB776	*LOC LUS*	2016.08.09											2				

SAMPLE ID	HOST	DATE	*I. ricinus* L	*I. ricinus* N	*I. frontalis* L	*I. frontalis* N	*I. frontalis* F	*I. festai* F	*I. festai* M	*I. lividus* N	*I. lividus* F	*Ha. concinna* L	*Ha. concinna* N	*Ha. punctata* L	*I. arboricola* N	*Hyalomma* sp. N	*D. reticulatus* F
FB777	*ACR RIS*	2016.08.09	1														
FB778	*LUS MEG*	2016.08.10	2														
FB779	*ACR SCI*	2016.08.10		1								14					
FB780	*ACR SCI*	2016.08.10		1													
FB781	*ACR SCI*	2016.08.11	2	1								1	2				
FB782	*SYL NIS*	2016.08.12	1														
FB783	*ACR RIS*	2016.08.12	1														
FB784	*ACR SCI*	2016.08.13											1				
FB785	*LOC LUS*	2016.08.13										10	5				
FB786	*LUS LUS*	2016.08.14	1	1													
FB787	*ERI RUB*	2016.08.15	1														
FB788	*ACR RIS*	2016.08.15		1													
FB789	*ACR SCI*	2016.08.16	1	1													
FB790	*TUR PHI*	2016.08.16	2										4				
FB791	*ACR SCI*	2016.08.17		1													
FB792	*LUS MEG*	2016.08.17	1														
FB793	*TUR MER*	2016.08.17	3	2								2	2				
FB794	*TRO TRO*	2016.08.18	1														
FB795	*SYL BOR*	2016.08.18		1													
FB796	*ERI RUB*	2016.08.19	7														
FB797	*SYL ATR*	2016.08.24	1														
FB798	*ERI RUB*	2016.08.24	2														
FB799	*SYL COM*	2016.08.24	1														
FB800	*ACR SCH*	2016.08.24											1				
FB801	*ERI RUB*	2016.08.24	1														
FB802	*ERI RUB*	2016.08.25	5														
FB804	*SYL ATR*	2016.08.25	2														
FB805	*SYL ATR*	2016.08.25	1														
FB806	*LUS MEG*	2016.08.25	1														
FB807	*LUS LUS*	2016.08.25	9	3													
FB808	*ACR RIS*	2016.08.25		1													
FB809	*SYL ATR*	2016.08.25	1	1													
FB810	*SYL COM*	2016.08.25		1													
FB811	*TUR MER*	2016.08.25	3	2													
FB812	*TUR MER*	2016.08.25	4	1													
FB813	*LUS LUS*	2016.08.26	2														
FB814	*ERI RUB*	2016.08.26	2														
FB815	*TUR PHI*	2016.08.26	2														
FB816	*SYL ATR*	2016.08.26	2														
FB817	*ERI RUB*	2016.08.26	1														
FB818	*ERI RUB*	2016.08.26	1														
FB819	*LUS LUS*	2016.08.27		3													
FB820	*TUR MER*	2016.08.27											1				
FB821	*ACR RIS*	2016.08.27		1													
FB822	*LUS MEG*	2016.08.28		1													
FB823	*ACR SCH*	2016.08.29										1	1				
FB824	*SYL ATR*	2016.08.30	1														
FB825	*SYL ATR*	2016.08.31		1													
FB826	*SYL COM*	2016.08.31		2													
FB827	*SYL ATR*	2016.09.01	1														
FB829	*LAN COL*	2016.09.01	1														
FB830	*TUR MER*	2016.09.01		2									1				
FB831	*TUR MER*	2016.09.01	5	2									2				
FB832	*ERI RUB*	2016.09.01	1														
FB833	*TUR PHI*	2016.09.02		3													
FB834	*ERI RUB*	2016.09.02	1														
FB835	*ERI RUB*	2016.09.03	4														
FB836	*SYL ATR*	2016.09.03		1													

SAMPLE ID	HOST	DATE	*I. ricinus* L	*I. ricinus* N	*I. frontalis* L	*I. frontalis* N	*I. frontalis* F	*I. festai* F	*I. festai* M	*I. lividus* N	*I. lividus* F	*Ha. concinna* L	*Ha. concinna* N	*Ha. punctata* L	*I. arboricola* N	*Hyalomma* sp. N	*D. reticulatus* F
FB837	*ACR RIS*	2016.09.03	1														
FB838	*SYL ATR*	2016.09.03		1													
FB839	*PAR MAJ*	2016.09.03		1													
FB840	*LUS LUS*	2016.09.03		1													
FB841	*ERI RUB*	2016.09.03	1														
FB842	*CAR CHL*	2016.09.05											2				
FB843	*SYL ATR*	2016.09.05	1														
FB844	*LUS MEG*	2016.09.05	2														
FB845	*ERI RUB*	2016.09.05	1														
FB846	*SYL ATR*	2016.09.05	1														
FB847	*ERI RUB*	2016.09.07	2														
FB848	*ERI RUB*	2016.09.07	7	1													
FB849	*ERI RUB*	2016.09.07	3														
FB850	*ERI RUB*	2016.09.07	1														
FB851	*ERI RUB*	2016.09.07										2					
FB852	*LUS SVE*	2016.09.08		1													
FB853	*SYL ATR*	2016.09.08	3	1													
FB854	*LAN COL*	2016.09.08	7	1													
FB855	*TUR MER*	2016.09.08		1									1				
FB856	*ERI RUB*	2016.09.08	3	1								1					
FB857	*ERI RUB*	2016.09.08	2														
FB858	*HIP ICT*	2016.09.08		1													
FB860	*ERI RUB*	2016.09.09	2														
FB861	*ERI RUB*	2016.09.09	1	1													
FB862	*SYL ATR*	2016.09.10	2														
FB863	*ERI RUB*	2016.09.10	1														
FB864	*ERI RUB*	2016.09.10	3														
FB865	*SYL ATR*	2016.09.10	2														
FB866	*SYL ATR*	2016.09.10	1														
FB867	*ERI RUB*	2016.09.10		1													
FB868	*ERI RUB*	2016.09.10		1													
FB869	*PHY TRO*	2016.09.10		1													
FB870	*SYL ATR*	2016.09.10	1														
FB871	*TUR PHI*	2016.09.10	1														
FB872	*LUS MEG*	2016.09.10		1													
FB873	*TUR MER*	2016.09.10		1													
FB874	*ACR ARU*	2016.09.11	1	1													
FB875	*ERI RUB*	2016.09.11	5														
FB876	*SYL ATR*	2016.09.13	2														
FB877	*PHO PHO*	2016.09.13		1													
FB878	*ERI RUB*	2016.09.14	1														
FB879	*ERI RUB*	2016.09.14	2	2													
FB880	*ERI RUB*	2016.09.14	2														
FB881	*TUR PHI*	2016.09.14		2													
FB882	*ERI RUB*	2016.09.14	1														
FB883	*ERI RUB*	2016.09.14	1														
FB884	*ERI RUB*	2016.09.15	1														
FB885	*SYL ATR*	2016.09.15	1														
FB886	*PHY TRO*	2016.09.15		1													
FB887	*ERI RUB*	2016.09.15	3														
FB888	*ERI RUB*	2016.09.15	3														
FB889	*PHY TRO*	2016.09.15	2	1													
FB890	*ERI RUB*	2016.09.15	1														
FB891	*SYL ATR*	2016.09.16	1														
FB892	*SYL ATR*	2016.09.16	1														
FB893	*SYL ATR*	2016.09.16	1														
FB894	*SYL ATR*	2016.09.16	1														
FB895	*ERI RUB*	2016.09.17		1													

SAMPLE ID	HOST	DATE	*I. ricinus* L	*I. ricinus* N	*I. frontalis* L	*I. frontalis* N	*I. frontalis* F	*I. festai* F	*I. festai* M	*I. lividus* N	*I. lividus* F	*Ha. concinna* L	*Ha. concinna* N	*Ha. punctata* L	*I. arboricola* N	*Hyalomma* sp. N	*D. reticulatus* F
FB896	*ACR SCI*	2016.09.18		1													
FB897	*SYL ATR*	2016.09.18		1													
FB898	*ERI RUB*	2016.09.18	1														
FB899	*ERI RUB*	2016.09.18	4														
FB900	*SYL ATR*	2016.09.20		1													
FB901	*SYL ATR*	2016.09.20	1	1													
FB902	*ERI RUB*	2016.09.20		1													
FB903	*SYL COM*	2016.09.20		2													
FB904	*SYL ATR*	2016.09.21		1													
FB905	*SYL ATR*	2016.09.21	1														
FB906	*SYL ATR*	2016.09.21		1													
FB907	*ERI RUB*	2016.09.21		1													
FB908	*ERI RUB*	2016.09.21	3	1													
FB909	*ERI RUB*	2016.09.21	1														
FB910	*SYL ATR*	2016.09.21		1													
FB911	*ERI RUB*	2016.09.21	4	1													
FB912	*ERI RUB*	2016.09.21		1													
FB913	*SYL ATR*	2016.09.22		1													
FB914	*SYL ATR*	2016.09.22	1														
FB915	*ERI RUB*	2016.09.22	9														
FB916	*SYL ATR*	2016.09.22		1													
FB917	*ERI RUB*	2016.09.22	2	1													
FB918	*ERI RUB*	2016.09.22	1														
FB919	*ERI RUB*	2016.09.23	3														
FB920	*TUR MER*	2016.09.23	1	1													
FB921	*ERI RUB*	2016.09.23	1	2													
FB922	*ERI RUB*	2016.09.23	1														
FB923	*ERI RUB*	2016.09.23	2														
FB924	*ERI RUB*	2016.09.24	2	2													
FB925	*ERI RUB*	2016.09.24	3	1													
FB927	*SYL ATR*	2016.09.24	1														
FB928	*SYL ATR*	2016.09.24	1														
FB929	*SYL ATR*	2016.09.24		1													
FB930	*SYL ATR*	2016.09.24	2														
FB931	*ERI RUB*	2016.09.24	1														
FB933	*ERI RUB*	2016.09.24	1	1													
FB934	*ERI RUB*	2016.09.24	1														
FB936	*ERI RUB*	2016.09.24		1													
FB937	*ERI RUB*	2016.09.24	2														
FB938	*ERI RUB*	2016.09.24		1													
FB939	*ERI RUB*	2016.09.24	1	2													
FB940	*ERI RUB*	2016.09.25	3	1													
FB941	*TUR PHI*	2016.09.26		2													
FB942	*ERI RUB*	2016.09.26	1	1													
FB943	*ERI RUB*	2016.09.26	2	1													
FB944	*ERI RUB*	2016.09.26		1													
FB945	*SYL ATR*	2016.09.26		1													
FB946	*ERI RUB*	2016.09.26	7	1													
FB947	*ERI RUB*	2016.09.26	9	1													
FB948	*SYL ATR*	2016.09.26		4													
FB949	*ERI RUB*	2016.09.27	1														
FB950	*ERI RUB*	2016.09.27	2														
FB951	*ERI RUB*	2016.09.28		3													
FB952	*ERI RUB*	2016.09.28	1	1													
FB953	*ERI RUB*	2016.09.28	3	1													
FB954	*ERI RUB*	2016.10.01	1														
FB955	*ERI RUB*	2016.10.01	1														
FB956	*ERI RUB*	2016.10.01	12														

SAMPLE ID	HOST	DATE	*I. ricinus* L	*I. ricinus* N	*I. frontalis* L	*I. frontalis* N	*I. frontalis* F	*I. festai* F	*I. festai* M	*I. lividus* N	*I. lividus* F	*Ha. concinna* L	*Ha. concinna* N	*Ha. punctata* L	*I. arboricola* N	*Hyalomma* sp. N	*D. reticulatus* F
FB957	*ERI RUB*	2016.10.01	2														
FB958	*PHY COL*	2016.10.01	1														
FB959	*ERI RUB*	2016.10.01	1														
FB960	*SYL ATR*	2016.10.01		1													
FB961	*SYL ATR*	2016.10.01	3														
FB962	*PRU MOD*	2016.10.01		1													
FB963	*SYL ATR*	2016.10.01		1													
FB964	*ERI RUB*	2016.10.01		2													
FB965	*ERI RUB*	2016.10.01		2													
FB966	*ERI RUB*	2016.10.01	2														
FB967	*ERI RUB*	2016.10.01	1														
FB968	*ERI RUB*	2016.10.01	1														
FB969	*ERI RUB*	2016.10.01	2														
FB970	*ERI RUB*	2016.10.01	1														
FB971	*ERI RUB*	2016.10.01		1													
FB972	*ERI RUB*	2016.10.01	4														
FB973	*TRO TRO*	2016.10.02	2														
FB974	*SYL ATR*	2016.10.02		1													
FB975	*ERI RUB*	2016.10.05		1													
FB976	*ERI RUB*	2016.10.05	3														
FB977	*ERI RUB*	2016.10.05	1														
FB978	*ERI RUB*	2016.10.05	1														
FB979	*TUR PHI*	2016.10.07		2													
FB980	*ERI RUB*	2016.10.07	5														
FB981	*ERI RUB*	2016.10.07	3														
FB982	*ERI RUB*	2016.10.07	9														
FB983	*ERI RUB*	2016.10.08	1														
FB984	*TUR MER*	2016.10.08		4													
FB985	*TRO TRO*	2016.10.08	1														
FB987	*ERI RUB*	2016.10.08		4													
FB988	*TUR MER*	2016.10.08		1													
FB989	*ERI RUB*	2016.10.08		1													
FB991	*ERI RUB*	2016.10.09	4														
FB992	*PAR CAE*	2016.10.09		1													
FB994	*TUR MER*	2016.10.10		4													
FB995	*FRI COE*	2016.10.10		1													
FB996	*FRI COE*	2016.10.10		1													
FB997	*ERI RUB*	2016.10.12		1													
FB998	*ERI RUB*	2016.10.13		1													
FB999	*ERI RUB*	2016.10.15	1														
FB1000	*ERI RUB*	2016.10.16		3													
FB1001	*ERI RUB*	2016.10.19		1													
FB1002	*PRU MOD*	2016.10.19		2													
FB1003	*PRU MOD*	2016.10.19		1													
FB1004	*ERI RUB*	2016.10.23	3														
FB1005	*ERI RUB*	2016.10.23	1														
FB1006	*ERI RUB*	2016.10.23		1													
FB1007	*ERI RUB*	2016.10.23	1	1													
FB1008	*PHY COL*	2016.10.27			1												
FB1009	*ERI RUB*	2016.10.27		1													
FB1010	*TUR MER*	2016.10.28		2													
FB1011	*TRO TRO*	2016.10.31	2														
FB1012	*SIT EUR*	2016.10.31		1													
FB1013	*PAR MAJ*	2016.10.31		1													
FB1014	*PYR PYR*	2016.11.01		1													
FB1015	*PAR MAJ*	2016.11.26					1										
FB1016	*PAR MAJ*	2016.12.03	1														
FB1017	*TUR MER*	2017.03.11		1													

SAMPLE ID	HOST	DATE	*I. ricinus* L	*I. ricinus* N	*I. frontalis* L	*I. frontalis* N	*I. frontalis* F	*I. festai* F	*I. festai* M	*I. lividus* N	*I. lividus* F	*Ha. concinna* L	*Ha. concinna* N	*Ha. punctata* L	*I. arboricola* N	*Hyalomma* sp. N	*D. reticulatus* F
FB1018	*TUR MER*	2017.03.11		8													
FB1019	*ERI RUB*	2017.03.12		1													
FB1020	*FRI COE*	2017.03.15		1													
FB1021	*TUR MER*	2017.03.17		5													
FB1022	*TUR MER*	2017.03.17		4													
FB1023	*PRU MOD*	2017.03.17		1													
FB1024	*ERI RUB*	2017.03.18		2													
FB1025	*TUR MER*	2017.03.18		9													
FB1026	*PRU MOD*	2017.03.18		2													
FB1027	*ERI RUB*	2017.03.18		1													
FB1028	*FRI COE*	2017.03.19		1													
FB1029	*TUR MER*	2017.03.19		1													
FB1030	*TUR MER*	2017.03.21						1	1								
FB1031	*TUR MER*	2017.03.21	1	1	12	3											
FB1032	*TUR MER*	2017.03.21		4													
FB1033	*TUR PHI*	2017.03.21		2		1											
FB1034	*PRU MOD*	2017.03.21	1														
FB1035	*ERI RUB*	2017.03.21		1													
FB1036	*TUR MER*	2017.03.22		1													
FB1037	*CAR CHL*	2017.03.22		1													
FB1038	*ERI RUB*	2017.03.24	1														
FB1039	*TRO TRO*	2017.03.24		1													
FB1040	*ERI RUB*	2017.03.24				1											
FB1041	*TUR PHI*	2017.03.24		2													
FB1042	*PRU MOD*	2017.03.24		1													
FB1043	*TUR MER*	2017.03.24		2													
FB1044	*TUR MER*	2017.03.24		3													
FB1045	*ERI RUB*	2017.03.24			26	5											
FB1046	*ERI RUB*	2017.03.24		1													
FB1047	*ERI RUB*	2017.03.24		1													
FB1048	*ERI RUB*	2017.03.25	1														
FB1049	*SYL ATR*	2017.03.25		1													
FB1050	*TUR PHI*	2017.03.27		1													
FB1051	*TUR PHI*	2017.03.28		1													
FB1052	*TUR MER*	2017.03.28		2													
FB1053	*TUR MER*	2017.03.28										1					
FB1055	*PRU MOD*	2017.03.30		1													
FB1056	*TUR PHI*	2017.03.30		1													
FB1057	*TUR MER*	2017.03.30		7													
FB1058	*TUR PHI*	2017.03.30		4													
FB1059	*ERI RUB*	2017.03.31		1													
FB1060	*AEG CAU*	2017.03.31		1													
FB1061	*ERI RUB*	2017.04.02	3														
FB1062	*PHY COL*	2017.04.02	1														
FB1063	*ERI RUB*	2017.04.02		1													
FB1064	*TUR PHI*	2017.04.04		2													
FB1065	*ERI RUB*	2017.04.04		1													
FB1066	*ERI RUB*	2017.04.08	1														
FB1067	*ERI RUB*	2017.04.08		1													
FB1068	*TUR MER*	2017.04.09		4													
FB1069	*TUR PHI*	2017.04.10		2													
FB1070	*PAR MAJ*	2017.03.13		1													
FB1071	*TUR MER*	2017.04.18		1													
FB1072	*TUR PHI*	2017.04.18		2													
FB1073	*FIC HYP*	2017.04.23	1	1													
FB1074	*TRO TRO*	2017.04.23		1													
FB1075	*TUR MER*	2017.04.29		2													
FB1076	*TUR MER*	2017.04.29		1													

SAMPLE ID	HOST	DATE	*I. ricinus* L	*I. ricinus* N	*I. frontalis* L	*I. frontalis* N	*I. frontalis* F	*I. festai* F	*I. festai* M	*I. lividus* N	*I. lividus* F	*Ha. concinna* L	*Ha. concinna* N	*Ha. punctata* L	*I. arboricola* N	*Hyalomma* sp. N	*D. reticulatus* F
FB1077	*TUR MER*	2017.05.02		6													
FB1078	*PAR MAJ*	2017.05.02		1													
FB1079	*FRI COE*	2017.05.10		4													
FB1080	*TUR MER*	2017.05.11	1	15													
FB1081	*TUR MER*	2017.05.23		2									1				
FB1082	*PAR MAJ*	2017.05.25		2													
FB1083	*SYL ATR*	2017.05.25											1				
FB1085	*TUR MER*	2017.05.26		6													
FB1087	*SYL ATR*	2017.05.29		1													
FB1088	*SYL ATR*	2017.05.31		1													
FB1089	*TUR MER*	2017.06.01											2				
FB1090	*CAR CHL*	2017.06.01		1													
FB1091	*TUR MER*	2017.06.07		4													
FB1093	*TUR MER*	2017.06.07		5													
FB1095	*SYL ATR*	2017.06.08		1													
FB1096	*TUR MER*	2017.06.08		1													
FB1097	*LUS MEG*	2017.06.08		1													
FB1098	*SYL ATR*	2017.06.08		1													
FB1099	*TUR PHI*	2017.06.08		5													
FB1100	*ACR SCI*	2017.06.08		1													
FB1101	*TUR PHI*	2017.06.17		1													
FB1102	*TUR PHI*	2017.06.17		1													
FB1103	*RIP RIP*	2017.06.19									7						
FB1104	*RIP RIP*	2017.06.19									2						
FB1105	*LOC LUS*	2017.06.24										2	6				
FB1106	*ACR SCI*	2017.06.24		1													
FB1107	*TUR MER*	2017.06.27		1								1	2				
FB1108	*TUR MER*	2017.06.27		1													
FB1109	*ACR SCI*	2017.07.04		1													
FB1110	*ERI RUB*	2017.07.04	2									1					
FB1119	*ACR SCH*	2017.07.04											1				
FB1120	*LOC LUS*	2017.07.04										1					
FB1122	*TUR MER*	2017.07.04		1									1				
FB1124	*LOC LUS*	2017.07.04											1				
FB1126	*ERI RUB*	2017.07.04		2													
FB1127	*TUR MER*	2017.07.04											1				
FB1128	*LOC FLU*	2017.07.04											1				
FB1129	*SYL ATR*	2017.07.10		1													
FB1130	*LUS MEG*	2017.07.10	9	1													
FB1131	*ACR SCI*	2017.07.10											1				
FB1133	*ACR SCI*	2017.07.10											1				
FB1135	*TUR MER*	2017.07.10											1				
FB1137	*ERI RUB*	2017.07.10	2														
FB1138	*LOC LUS*	2017.07.11										1					
FB1139	*ACR SCH*	2017.07.11											1				
FB1140	*TUR MER*	2017.07.11		2								1	2				
FB1141	*TUR PHI*	2017.07.11		1									6				
FB1142	*LUS MEG*	2017.07.11		1													
FB1143	*ACR SCI*	2017.07.12		1													
FB1144	*ACR RIS*	2017.07.12	1	2									2				
FB1145	*ACR RIS*	2017.07.12										5	1				
FB1146	*ACR SCH*	2017.07.12										8	3				
FB1147	*HIP ICT*	2017.07.12		1													
FB1148	*LOC LUS*	2017.07.12											2				
FB1149	*LOC FLU*	2017.07.12		1													
FB1152	*LOC LUS*	2017.07.13										4	4				
FB1153	*TUR MER*	2017.07.14		2									1				
FB1154	*ACR SCH*	2017.07.14											1				

SAMPLE ID	HOST	DATE	*I. ricinus* L	*I. ricinus* N	*I. frontalis* L	*I. frontalis* N	*I. frontalis* F	*I. festai* F	*I. festai* M	*I. lividus* N	*I. lividus* F	*Ha. concinna* L	*Ha. concinna* N	*Ha. punctata* L	*I. arboricola* N	*Hyalomma* sp. N	*D. reticulatus* F
FB1155	*LOC LUS*	2017.07.14											1				
FB1156	*TUR MER*	2017.07.14											1				
FB1157	*LUS MEG*	2017.07.15											2				
FB1158	*PHY COL*	2017.07.15		1													
FB1159	*ACR SCI*	2017.07.15											1				
FB1160	*TUR MER*	2017.07.15											1				
FB1162	*LOC LUS*	2017.07.17											3				
FB1163	*ACR SCI*	2017.07.17		1													
FB1164	*TUR MER*	2017.07.17										2	3				
FB1165	*LUS MEG*	2017.07.17	1	1													
FB1166	*TUR MER*	2017.07.17											1				
FB1167	*LUS MEG*	2017.07.18		1													
FB1168	*LOC LUS*	2017.07.19										17					
FB1169	*ACR RIS*	2017.07.20		1									1				
FB1172	*LOC LUS*	2017.07.21											2				
FB1173	*PHY TRO*	2017.07.21	1														
FB1177	*LUS MEG*	2017.07.22											2				
FB1178	*ACR RIS*	2017.07.22		1													
FB1179	*ACR SCI*	2017.07.22		1													
FB1180	*LOC LUS*	2017.07.23											1				
FB1181	*ACR RIS*	2017.07.23		1													
FB1182	*ACR RIS*	2017.07.23											1				
FB1183	*ACR SCI*	2017.07.24		1													
FB1184	*ACR RIS*	2017.07.24											2				
FB1185	*LOC LUS*	2017.07.24		1													
FB1186	*PHY COL*	2017.07.26		1													
FB1188	*ACR SCI*	2017.07.27		2								1					
FB1189	*LAN COL*	2017.07.27		1													
FB1190	*ACR ARU*	2017.07.28	1														
FB1191	*ACR SCI*	2017.07.28		1													
FB1192	*ACR ARU*	2017.07.28	1														
FB1193	*ACR SCH*	2017.07.28										1	4				
FB1194	*LUS MEG*	2017.07.29	1	1													
FB1195	*ACR RIS*	2017.07.29		1													
FB1196	*ACR RIS*	2017.07.29	3	1													
FB1197	*LUS MEG*	2017.07.29	2														
FB1198	*LOC LUS*	2017.07.29											2				
FB1199	*ACR RIS*	2017.07.29	1	1													
FB1201	*ACR ARU*	2017.07.30										1	1				
FB1202	*LOC FLU*	2017.07.30										3	2				
FB1203	*LOC LUS*	2017.07.30											1				
FB1204	*ACR SCI*	2017.07.30											1				
FB1205	*LUS MEG*	2017.07.30	2										1				
FB1206	*LOC LUS*	2017.07.30											1				
FB1207	*LOC LUS*	2017.07.30										1	4				
FB1208	*LOC LUS*	2017.07.30											3				
FB1210	*LOC LUS*	2017.08.02		1								9	3				
FB1211	*LOC LUS*	2017.08.02										1					
FB1213	*LOC LUS*	2017.08.02											2				
FB1214	*TUR PHI*	2017.08.03	1														
FB1215	*ACR ARU*	2017.08.04											1				
FB1216	*LUS MEG*	2017.08.04	2														
FB1217	*LOC LUS*	2017.08.05											2				
FB1218	*ACR RIS*	2017.08.06	2														
FB1219	*ACR RIS*	2017.08.06										2					
FB1220	*ACR RIS*	2017.08.06		1													
FB1221	*LUS MEG*	2017.08.06		1													
FB1222	*ERI RUB*	2017.08.08		1													

SAMPLE ID	HOST	DATE	*I. ricinus* L	*I. ricinus* N	*I. frontalis* L	*I. frontalis* N	*I. frontalis* F	*I. festai* F	*I. festai* M	*I. lividus* N	*I. lividus* F	*Ha. concinna* L	*Ha. concinna* N	*Ha. punctata* L	*I. arboricola* N	*Hyalomma* sp. N	*D. reticulatus* F
FB1223	*LOC LUS*	2017.08.08											3				
FB1224	*SYL COM*	2017.08.09		2													
FB1225	*LUS LUS*	2017.08.09	1	2													
FB1226	*TUR MER*	2017.08.09										3	1				
FB1228	*ACR SCI*	2017.08.11											1				
FB1229	*ACR SCI*	2017.08.11											1				
FB1230	*STU VUL*	2017.08.14											1				
FB1231	*ACR RIS*	2017.08.16		1								3					
FB1233	*PHY COL*	2017.08.16	2	1								1					
FB1234	*SYL COM*	2017.08.18		1													
FB1235	*ACR RIS*	2017.08.18		3													
FB1236	*TUR MER*	2017.08.19										1					
FB1237	*LUS MEG*	2017.08.19	1									3					
FB1238	*LOC LUS*	2017.08.19										2	1				
FB1239	*ACR RIS*	2017.08.21		3													
FB1240	*LOC LUS*	2017.08.21										15	1				
FB1241	*ACR RIS*	2017.08.21		1													
FB1242	*ACR RIS*	2017.08.21	5	3													
FB1244	*ACR RIS*	2017.08.22	2	1													
FB1245	*ACR SCH*	2017.08.22	1														
FB1246	*TUR MER*	2017.08.22	4										1				
FB1247	*TRO TRO*	2017.08.23	4														
FB1248	*ACR RIS*	2017.08.24	2	1													
FB1249	*ACR SCI*	2017.08.25		1													
FB1250	*SYL BOR*	2017.08.25		1													
FB1251	*ACR RIS*	2017.08.25		1								1					
FB1253	*SYL ATR*	2017.08.28		1													
FB1254	*ACR SCI*	2017.08.28		1													
FB1255	*ACR SCH*	2017.08.29		1													
FB1256	*ACR SCI*	2017.08.29		1													
FB1257	*LOC FLU*	2017.08.30											1				
FB1258	*LOC LUS*	2017.08.30										5	2				
FB1259	*LUS LUS*	2017.08.31	1														
FB1260	*SYL ATR*	2017.08.31	1	2													
FB1261	*LUS MEG*	2017.08.31	1														
FB1262	*ERI RUB*	2017.08.31	3														
FB1263	*ERI RUB*	2017.09.02	1														
FB1264	*LOC LUS*	2017.09.02											1				
FB1267	*SYL ATR*	2017.09.09		1													
FB1268	*SYL ATR*	2017.09.09	2														
FB1269	*ERI RUB*	2017.09.09	1	1													
FB1270	*SYL ATR*	2017.09.09	1														
FB1271	*ACR SCI*	2017.09.09	1	1													
FB1272	*SYL ATR*	2017.09.10	1														
FB1273	*ACR SCI*	2017.09.10											1				
FB1275	*SYL ATR*	2017.09.11		1													
FB1276	*ERI RUB*	2017.09.11		1													
FB1277	*PHY COL*	2017.09.11	3														
FB1279	*ACR RIS*	2017.09.13		3													
FB1280	*SYL ATR*	2017.09.16	10	1													
FB1281	*ERI RUB*	2017.09.23	1														
FB1282	*ERI RUB*	2017.09.23	1														
FB1283	*ERI RUB*	2017.09.24		1													
FB1288	*ERI RUB*	2017.09.28		1													
FB1289	*TUR MER*	2017.09.29		1													
FB1290	*ERI RUB*	2017.09.30		1													
FB1291	*TUR PHI*	2017.09.30		1													
FB1292	*TUR MER*	2017.09.30		1													

SAMPLE ID	HOST	DATE	*I. ricinus* L	*I. ricinus* N	*I. frontalis* L	*I. frontalis* N	*I. frontalis* F	*I. festai* F	*I. festai* M	*I. lividus* N	*I. lividus* F	*Ha. concinna* L	*Ha. concinna* N	*Ha. punctata* L	*I. arboricola* N	*Hyalomma* sp. N	*D. reticulatus* F
FB1293	*SYL ATR*	2017.09.30		1													
FB1294	*SYL ATR*	2017.09.30	1														
FB1295	*ERI RUB*	2017.09.30		1													
FB1296	*ERI RUB*	2017.09.30	3	1													
FB1297	*ERI RUB*	2017.10.01		1													
FB1298	*PHY COL*	2017.10.02		1													
FB1299	*ERI RUB*	2017.10.02	3														
FB1300	*PAR MAJ*	2017.10.06		2													
FB1301	*ERI RUB*	2017.10.06		1													
FB1302	*ERI RUB*	2017.10.07	6	1													
FB1303	*ERI RUB*	2017.10.07	4	1													
FB1304	*ERI RUB*	2017.10.07		1													
FB1305	*ERI RUB*	2017.10.07	3	1													
FB1307	*ERI RUB*	2017.10.07	2														
FB1308	*ERI RUB*	2017.10.07		1													
FB1309	*ERI RUB*	2017.10.07	1														
FB1310	*SYL ATR*	2017.10.07		1													
FB1311	*ERI RUB*	2017.10.07		1													
FB1312	*ERI RUB*	2017.10.07		1													
FB1313	*ERI RUB*	2017.10.07		1													
FB1314	*ERI RUB*	2017.10.07	1	1													
FB1315	*ERI RUB*	2017.10.07		1													
FB1316	*ERI RUB*	2017.10.07	1														
FB1317	*ERI RUB*	2017.10.07	3	6													
FB1318	*TRO TRO*	2017.10.14		1													
FB1319	*ERI RUB*	2017.10.14	1														
FB1320	*TRO TRO*	2017.10.14		1													
FB1321	*TRO TRO*	2017.10.14	1	1													
FB1323	*ERI RUB*	2017.10.15	1														
FB1324	*PAR MAJ*	2017.10.16		1													
FB1325	*ERI RUB*	2017.10.17	1														
FB1326	*PAR MAJ*	2017.10.17		1													
FB1327	*TUR PHI*	2017.10.19		1													
FB1329	*TRO TRO*	2017.10.19	2	1													
FB1330	*ERI RUB*	2017.10.21		1													
FB1331	*TUR PHI*	2017.10.21		1													
FB1332	*TUR MER*	2017.10.21		1													
FB1333	*TUR PHI*	2017.10.21		1													
FB1334	*ERI RUB*	2017.10.22	1														
FB1335	*PAR MAJ*	2017.11.05					1										
FB1336	*FRI MON*	2017.11.05		1													
FB1337	*TUR MER*	2017.11.10		1													
FB1338	*TRO TRO*	2017.10.14		1													
FB1339	*PRU MOD*	2017.10.17		1													
FB1340	*PYR PYR*	2017.10.18		1													
FB1341	*PAR CAE*	2018.01.03		1													
FB1342	*ERI RUB*	2018.03.10		1													
FB1343	*COC COC*	2018.03.14				1											
FB1344	*TUR MER*	2018.03.15		1													
FB1345	*TUR MER*	2018.03.15		1													
FB1346	*PRU MOD*	2018.03.15	2														
FB1347	*TUR PHI*	2018.03.15		1													
FB1348	*PAR MAJ*	2018.03.15		1													
FB1349	*TUR MER*	2018.03.15		1													
FB1350	*PAR MAJ*	2018.03.19		1											1		
FB1351	*FRI MON*	2018.03.19		1													
FB1352	*TUR MER*	2018.03.21		1													
FB1353	*TUR MER*	2018.03.25		1													

SAMPLE ID	HOST	DATE	*I. ricinus* L	*I. ricinus* N	*I. frontalis* L	*I. frontalis* N	*I. frontalis* F	*I. festai* F	*I. festai* M	*I. lividus* N	*I. lividus* F	*Ha. concinna* L	*Ha. concinna* N	*Ha. punctata* L	*I. arboricola* N	*Hyalomma* sp. N	*D. reticulatus* F
FB1354	*PHY COL*	2018.03.26		1													
FB1355	*PAR MAJ*	2018.03.26		2													
FB1356	*PHY COL*	2018.03.26		1													
FB1357	*PAR MAJ*	2018.03.26		1													
FB1359	*ERI RUB*	2018.03.28		1													
FB1360	*TUR PHI*	2018.03.28		1													
FB1361	*ERI RUB*	2018.03.28		1													
FB1362	*ERI RUB*	2018.03.28		2													
FB1363	*TUR MER*	2018.03.28		2													
FB1364	*ERI RUB*	2018.03.28					1										
FB1365	*TUR MER*	2018.03.29		6													
FB1366	*TRO TRO*	2018.03.29		1													
FB1367	*ERI RUB*	2018.03.30		1													
FB1368	*PAR MAJ*	2018.03.30		2													
FB1369	*PAR MAJ*	2018.03.30		1													
HS001	*TUR PHI*	2018.03.31				1											
HS002	*ERI RUB*	2018.03.31				2											
HS003	*TUR MER*	2018.03.31		1													
HS004	*PAR MAJ*	2018.03.31		2													
HS005	*TUR MER*	2018.04.01		2													
HS006	*ERI RUB*	2018.04.02	1	1													
HS007	*ERI RUB*	2018.04.02	1														
HS008	*ERI RUB*	2018.04.03		1													
HS009	*PAR MAJ*	2018.04.03		5													
HS010	*ERI RUB*	2018.04.03		2													
HS011	*ERI RUB*	2018.04.03		1													
HS012	*TUR PHI*	2018.04.03		2													
HS013	*FRI COE*	2018.04.03		1													
HS014	*TUR MER*	2018.04.04		5													
HS015	*SYL ATR*	2018.04.04		2													
HS016	*CAR CHL*	2018.04.04		1													
HS017	*ERI RUB*	2018.04.04		1													
HS018	*PAR CAE*	2018.04.05		1													
HS019	*SYL CUR*	2018.04.05	1														
HS020	*TUR MER*	2018.04.05		6													
HS021	*TUR PHI*	2018.04.05		1													
HS022	*PAR MAJ*	2018.04.06		1													
HS023	*ERI RUB*	2018.04.06		1													
HS024	*TUR MER*	2018.04.06		1													
HS025	*TUR MER*	2018.04.06		3													
HS026	*ERI RUB*	2018.04.07		1													
HS027	*FRI COE*	2018.04.07	1	1													
HS028	*CAR CHL*	2018.04.07		1													
HS029	*TUR MER*	2018.04.07		11													
HS030	*ERI RUB*	2018.04.07		2													
HS031	*PAR MAJ*	2018.04.07		2													
HS032	*TUR PHI*	2018.04.07		1													
HS033	*SYL ATR*	2018.04.09		1													
HS034	*FRI COE*	2018.04.09		2													
HS035	*LUS MEG*	2018.04.09		1													
HS036	*SYL ATR*	2018.04.12		1													
HS037	*TUR PHI*	2018.04.12		1													
HS038	*TUR MER*	2018.04.14		1													
HS039	*SYL ATR*	2018.04.14		1													
HS040	*TUR MER*	2018.04.14		1													
HS041	*TUR MER*	2018.04.14		1													
HS042	*PAS MON*	2018.04.15		1													
HS043	*CAR CHL*	2018.04.17		2													

SAMPLE ID	HOST	DATE	*I. ricinus* L	*I. ricinus* N	*I. frontalis* L	*I. frontalis* N	*I. frontalis* F	*I. festai* F	*I. festai* M	*I. lividus* N	*I. lividus* F	*Ha. concinna* L	*Ha. concinna* N	*Ha. punctata* L	*I. arboricola* N	*Hyalomma* sp. N	*D. reticulatus* F
HS044	*CAR CHL*	2018.04.21		1													
HS045	*PHY COL*	2018.04.24		1													
HS046	*TUR MER*	2018.04.24		1									1				
HS047	*LUS MEG*	2018.04.26	1														
HS048	*TUR MER*	2018.05.10		1													
HS049	*LUS MEG*	2018.05.10		1													
HS050	*TUR MER*	2018.05.11		1													
HS051	*TUR MER*	2018.05.17		1									1				
HS052	*SYL ATR*	2018.05.17		2													
HS053	*TUR PHI*	2018.05.18											1				
HS054	*LUS MEG*	2018.05.24		1													
HS055	*CAR CHL*	2018.05.26		1													
HS057	*TUR PHI*	2018.05.29	1	1													
HS058	*SIT EUR*	2018.05.29		1													
HS059	*PAR MAJ*	2018.05.29		1													
HS060	*ACR RIS*	2018.06.01											1				
HS061	*TUR MER*	2018.06.06											1				
HS062	*SYL ATR*	2018.06.08		1													
HS063	*PAR MAJ*	2018.06.08		1													
HS067	*SYL ATR*	2018.06.17		1									1				
HS068	*PHY COL*	2018.06.17		1													
HS070	*LOC LUS*	2018.06.26										3	1				
HS071	*CER BRA*	2018.06.26		1													
HS072	*LOC LUS*	2018.06.30											2				
HS073	*TRO TRO*	2018.06.30	1														
HS074	*LOC LUS*	2018.06.30										2	1				
HS078	*ACR SCH*	2018.06.30		1													
HS079	*ACR SCI*	2018.06.30		1													
HS086	*PAR MAJ*	2018.07.06		1													
HS088	*ACR SCI*	2018.07.07										1	1				
HS089	*ACR RIS*	2018.07.07											1				
HS090	*ACR SCI*	2018.07.07											1				
HS091	*ACR SCI*	2018.07.07		1													
HS092	*ACR SCI*	2018.07.07											1				
HS094	*ERI RUB*	2018.07.08		1									1				
HS095	*LAN COL*	2018.07.10											1				
HS096	*LUS MEG*	2018.07.10		1													
HS097	*TUR PHI*	2018.07.10	2	1													
HS098	*ERI RUB*	2018.07.10											1				
HS099	*ERI RUB*	2018.07.10	1	1													
HS100	*ERI RUB*	2018.07.10	1														
HS102	*ACR SCI*	2018.07.10		1													
HS103	*ACR RIS*	2018.07.10		1													
HS104	*TUR PHI*	2018.07.10	4										1				
HS106	*TUR PHI*	2018.07.10										1					
HS107	*TUR MER*	2018.07.10											1				
HS108	*TUR PHI*	2018.07.10											2				
HS110	*ACR SCI*	2018.07.12											1				
HS111	*LUS MEG*	2018.07.12	1														
HS112	*TRO TRO*	2018.07.12	2														
HS113	*PAR MAJ*	2018.07.12	1														
HS114	*LOC LUS*	2018.07.12										1					
HS115	*LOC LUS*	2018.07.12											1				
HS118	*TUR PHI*	2018.07.14	14														
HS119	*ACR RIS*	2018.07.15		1								1	1				
HS120	*SYL ATR*	2018.07.15	1														
HS122	*LOC LUS*	2018.07.15											2				
HS123	*ACR SCH*	2018.07.15											1				

SAMPLE ID	HOST	DATE	*I. ricinus* L	*I. ricinus* N	*I. frontalis* L	*I. frontalis* N	*I. frontalis* F	*I. festai* F	*I. festai* M	*I. lividus* N	*I. lividus* F	*Ha. concinna* L	*Ha. concinna* N	*Ha. punctata* L	*I. arboricola* N	*Hyalomma* sp. N	*D. reticulatus* F
HS124	*LOC LUS*	2018.07.16											2				
HS125	*LOC LUS*	2018.07.16											1				
HS126	*LOC LUS*	2018.07.16		1									1				
HS129	*ACR RIS*	2018.07.18		2													
HS130	*ACR SCH*	2018.07.19											1				
HS131	*TUR PHI*	2018.07.20		1									1				
HS132	*ACR SCI*	2018.07.20		1													
HS134	*LOC LUS*	2018.07.20											1				
HS135	*TUR MER*	2018.07.21		1													
HS136	*ACR RIS*	2018.07.21		1													
HS137	*PAR MAJ*	2018.07.21		1													
HS138	*LUS MEG*	2018.07.21	2														
HS139	*TUR MER*	2018.07.21											1				
HS140	*LOC LUS*	2018.07.21										4	8				
HS141	*LUS MEG*	2018.07.22	1														
HS142	*LOC LUS*	2018.07.22											1				
HS143	*ACR SCI*	2018.07.22											1				
HS144	*ACR SCI*	2018.07.24											1				
HS145	*LUS MEG*	2018.07.24											1				
HS146	*LOC LUS*	2018.07.25											3				
HS147	*LUS MEG*	2018.07.25		1													
HS148	*TUR MER*	2018.07.25		1													
HS150	*ERI RUB*	2018.07.26	1														
HS151	*ACR SCI*	2018.07.26		1													
HS152	*LUS MEG*	2018.07.26	3														
HS153	*LUS MEG*	2018.07.26		2													
HS154	*LUS MEG*	2018.07.26		1													
HS155	*ACR SCI*	2018.07.26	2														
HS156	*LOC LUS*	2018.07.27											2				
HS157	*ACR SCI*	2018.07.27		1													
HS158	*LOC LUS*	2018.07.27											3				
HS159	*LOC LUS*	2018.07.28											2				
HS160	*LOC LUS*	2018.07.28											1				
HS162	*LOC LUS*	2018.07.29										8	9				
HS163	*LOC LUS*	2018.07.29											2				
HS164	*LOC LUS*	2018.07.29											1				
HS165	*LOC LUS*	2018.07.30											1				
HS166	*LOC LUS*	2018.07.30											1				
HS167	*LOC LUS*	2018.07.30											2				
HS168	*EMB SCH*	2018.07.30											3				
HS169	*LUS MEG*	2018.07.31	6														
HS170	*LOC LUS*	2018.07.31											1				
HS171	*ACR SCH*	2018.07.31											2				
HS172	*ACR RIS*	2018.08.01		1													
HS173	*LOC LUS*	2018.08.01											2				
HS174	*LUS LUS*	2018.08.01	4	1													
HS175	*LUS MEG*	2018.08.01		1													
HS176	*ACR SCI*	2018.08.02		1													
HS177	*ACR SCH*	2018.08.02											2				
HS178	*LOC LUS*	2018.08.03										4	3				
HS179	*TUR PHI*	2018.08.04											4				
HS180	*ACR ARU*	2018.08.04		1													
HS181	*SYL COM*	2018.08.05	5														
HS182	*LOC LUS*	2018.08.05											1				
HS184	*PAR MAJ*	2018.08.06	1														
HS185	*LOC LUS*	2018.08.06											1				
HS186	*LOC LUS*	2018.08.06											1				
HS187	*ACR RIS*	2018.08.06		1													

SAMPLE ID	HOST	DATE	*I. ricinus* L	*I. ricinus* N	*I. frontalis* L	*I. frontalis* N	*I. frontalis* F	*I. festai* F	*I. festai* M	*I. lividus* N	*I. lividus* F	*Ha. concinna* L	*Ha. concinna* N	*Ha. punctata* L	*I. arboricola* N	*Hyalomma* sp. N	*D. reticulatus* F
HS188	*SYL ATR*	2018.08.07	1														
HS189	*LOC LUS*	2018.08.07											1				
HS190	*LUS MEG*	2018.08.07	4	1													
HS191	*ACR SCH*	2018.08.07										23	6				
HS192	*TUR MER*	2018.08.07	1	1													
HS193	*ACR SCI*	2018.08.07										1					
HS195	*SYL ATR*	2018.08.07										1					
HS196	*TUR MER*	2018.08.07		1									1				
HS197	*ACR SCI*	2018.08.08										1					
HS198	*SYL ATR*	2018.08.08	1														
HS199	*ACR SCI*	2018.08.08		1													
HS200	*ACR ARU*	2018.08.08	1	3													
HS201	*RAL AQU*	2018.08.08											1				
HS202	*COT COT*	2018.08.09										2					
HS203	*LUS LUS*	2018.08.10		1													
HS204	*LOC LUS*	2018.08.10										5	4				
HS205	*LOC LUS*	2018.08.10											3				
HS206	*LOC LUS*	2018.08.10										4	1				
HS207	*SYL ATR*	2018.08.11	1														
HS208	*LOC LUS*	2018.08.11											4				
HS209	*LOC LUS*	2018.08.11											1				
HS210	*SYL ATR*	2018.08.11	1														
HS211	*LOC LUS*	2018.08.11											3				
HS212	*LOC LUS*	2018.08.12											1				
HS213	*TUR PHI*	2018.08.12											1				
HS214	*ACR RIS*	2018.08.12		1													
HS215	*LUS LUS*	2018.08.13	13	2													
HS216	*ACR SCH*	2018.08.13											1				
HS217	*LOC LUS*	2018.08.14											1				
HS218	*ACR SCH*	2018.08.15										6					
HS219	*ACR SCI*	2018.08.15										2	1				
HS220	*LOC LUS*	2018.08.15										4	11				
HS222	*LOC LUS*	2018.08.18											2				
HS223	*LOC LUS*	2018.08.18										17	2				
HS224	*SYL ATR*	2018.08.19	1														
HS225	*SYL COM*	2018.08.19	1														
HS226	*LUS MEG*	2018.08.19	3														
HS227	*ACR SCI*	2018.08.19		1													
HS228	*ACR SCI*	2018.08.19	1										1				
HS229	*SYL ATR*	2018.08.20		1													
HS230	*SYL ATR*	2018.08.20	1	1													
HS231	*ERI RUB*	2018.08.20	1														
HS232	*ACR RIS*	2018.08.20	1	1													
HS233	*SYL ATR*	2018.08.20	3														
HS235	*SYL ATR*	2018.08.21	1														
HS236	*LUS LUS*	2018.08.21		1													
HS237	*LOC LUS*	2018.08.21										7	3				
HS238	*LOC LUS*	2018.08.21										19	2				
HS239	*ACR SCI*	2018.08.21		1													
HS240	*ERI RUB*	2018.08.21	1														
HS241	*ACR SCI*	2018.08.21		2													
HS243	*SYL BOR*	2018.08.22		2													
HS244	*ACR SCI*	2018.08.22										14					
HS245	*LOC LUS*	2018.08.23										1	1				
HS246	*ACR SCH*	2018.08.24		1													
HS247	*ERI RUB*	2018.08.25	1	1													
HS248	*SYL COM*	2018.08.25		2													
HS249	*LUS MEG*	2018.08.25	3														

SAMPLE ID	HOST	DATE	*I. ricinus* L	*I. ricinus* N	*I. frontalis* L	*I. frontalis* N	*I. frontalis* F	*I. festai* F	*I. festai* M	*I. lividus* N	*I. lividus* F	*Ha. concinna* L	*Ha. concinna* N	*Ha. punctata* L	*I. arboricola* N	*Hyalomma* sp. N	*D. reticulatus* F
HS250	*LOC LUS*	2018.08.25										1	3				
HS251	*PHY TRO*	2018.08.28	1														
HS252	*SYL ATR*	2018.08.28	1														
HS253	*ACR SCI*	2018.08.28	1									1					
HS254	*ERI RUB*	2018.08.28		1													
HS255	*SYL ATR*	2018.08.29		1													
HS257	*LOC LUS*	2018.08.30											1				
HS259	*SYL ATR*	2018.08.30		1													
HS260	*ERI RUB*	2018.09.01	4														
HS261	*SYL ATR*	2018.09.05	5														
HS262	*SYL COM*	2018.09.06		1													
HS263	*SYL ATR*	2018.09.08	1														
HS264	*SYL ATR*	2018.09.09	2														
HS265	*ACR ARU*	2018.09.09		1													
HS267	*SYL ATR*	2018.09.11		1													
HS268	*ANT TRI*	2018.09.14		1													
HS269	*PHY TRO*	2018.09.15		1													
HS270	*SYL ATR*	2018.09.15		1													
HS271	*SYL ATR*	2018.09.15		1													
HS272	*SYL BOR*	2018.09.16		1													
HS273	*ERI RUB*	2018.09.18		2													
HS275	*TUR PHI*	2018.09.29	1	1													
HS276	*ERI RUB*	2018.09.29	7														
HS277	*ERI RUB*	2018.09.29		1													
HS278	*TUR MER*	2018.09.29		1													
HS279	*TUR MER*	2018.09.30		1													
HS280	*ERI RUB*	2018.09.30	2														
HS281	*TUR PHI*	2018.10.01		1													
HS282	*ERI RUB*	2018.10.02	2														
HS283	*ERI RUB*	2018.10.03	1														
HS284	*SYL ATR*	2018.10.03	1														
HS285	*ERI RUB*	2018.10.06		1													
HS286	*ERI RUB*	2018.10.06	2														
HS287	*TUR MER*	2018.10.06		1													
HS289	*PAR MAJ*	2018.10.11		1													
HS290	*TUR PHI*	2018.10.14		1													
HS291	*TRO TRO*	2018.10.17		1													
HS292	*ERI RUB*	2018.10.17	1	1													
HS293	*TRO TRO*	2018.10.19	4														
HS294	*TUR MER*	2018.10.20		1													
HS295	*ERI RUB*	2018.10.20		1													
HS296	*ERI RUB*	2018.10.20		1													
HS297	*ERI RUB*	2018.10.20		2													
HS298	*TUR MER*	2018.10.20	1	1													
HS299	*ERI RUB*	2018.10.20	2														
HS300	*ERI RUB*	2018.10.20		2													
HS301	*ERI RUB*	2018.10.20		1													
HS302	*ERI RUB*	2018.10.21	2	1													
HS303	*ERI RUB*	2018.10.22	2														
HS304	*ERI RUB*	2018.10.22	1														
HS305	*PAR MAJ*	2018.10.22		1													
HS306	*ERI RUB*	2018.10.27	1														
HS307	*ERI RUB*	2018.11.01	6														
HS308	*TUR MER*	2018.11.03		1													
HS309	*ERI RUB*	2018.11.05	1														
HS310	*ERI RUB*	2018.11.10	1														
HS311	*TUR MER*	2018.11.10		2													
HS312	*TUR MER*	2018.11.10		1													

SAMPLE ID	HOST	DATE	*I. ricinus* L	*I. ricinus* N	*I. frontalis* L	*I. frontalis* N	*I. frontalis* F	*I. festai* F	*I. festai* M	*I. lividus* N	*I. lividus* F	*Ha. concinna* L	*Ha. concinna* N	*Ha. punctata* L	*I. arboricola* N	*Hyalomma* sp. N	*D. reticulatus* F
HS313	*ERI RUB*	2019.03.12		1													
HS314	*PRU MOD*	2019.03.13					1										
HS315	*FRI COE*	2019.03.14		1													
HS316	*ERI RUB*	2019.03.15				1											
HS317	*TUR MER*	2019.03.16		2													
HS318	*TUR MER*	2019.03.16		1													
HS319	*FRI COE*	2019.03.16		1													
HS320	*TUR MER*	2019.03.17		3													
HS321	*ERI RUB*	2019.03.18		1													
HS322	*TUR MER*	2019.03.21		3													
HS323	*TUR MER*	2019.03.23		2													
HS324	*TUR MER*	2019.03.23		1													
HS325	*FRI COE*	2019.03.24		1													
HS326	*ERI RUB*	2019.03.24		1													
HS327	*ERI RUB*	2019.03.25		1													
HS328	*TUR MER*	2019.03.25		2													
HS329	*ERI RUB*	2019.03.25				1											
HS330	*ERI RUB*	2019.03.25		1													
HS331	*TUR PHI*	2019.03.28		2													
HS332	*PAR MAJ*	2019.03.28		1													
HS333	*TUR MER*	2019.03.28		2													
HS334	*TUR MER*	2019.03.28		2													
HS335	*ERI RUB*	2019.03.28		1													
HS336	*ERI RUB*	2019.03.28		2													
HS337	*TUR TOR*	2019.03.29		2													
HS338	*TUR MER*	2019.03.29		2													
HS339	*ERI RUB*	2019.03.29		1													
HS340	*TUR MER*	2019.03.30		15													
HS341	*ERI RUB*	2019.03.30		2													
HS343	*ERI RUB*	2019.04.01		2													
HS344	*TUR MER*	2019.04.02		1													
HS345	*TUR PHI*	2019.04.06		3													
HS346	*LUS MEG*	2019.04.06		3													
HS347	*ERI RUB*	2019.04.08		1													
HS348	*ERI RUB*	2019.04.11		1													
HS349	*ERI RUB*	2019.04.11		1													
HS350	*LUS MEG*	2019.04.11	1	1													
HS351	*PAR MAJ*	2019.04.11		1													
HS352	*SYL CUR*	2019.04.13		1													
HS353	*ERI RUB*	2019.04.17		1													
HS354	*TUR MER*	2019.04.18		1													
HS355	*ERI RUB*	2019.04.20		1													
HS356	*SYL ATR*	2019.04.20		1													
HS357	*TUR MER*	2019.04.21		9													
HS358	*TUR MER*	2019.04.21		2													
HS359	*TUR MER*	2019.04.21		7													
HS360	*PHY COL*	2019.04.29		1													
HS361	*LUS MEG*	2019.05.02	2	2													
HS362	*LUS MEG*	2019.05.03		1													
HS364	*ACR SCI*	2019.05.20		1													
HS365	*PAS MON*	2019.05.23		1									1				
HS366	*SYL ATR*	2019.05.29					1										
HS367	*TUR MER*	2019.05.31		3													
HS368	*ACR MEL*	2019.05.31											1				
HS369	*TUR MER*	2019.06.05		3									2				
HS370	*LAN COL*	2019.06.05		1													
HS371	*TUR PHI*	2019.06.14		3													
HS372	*TUR MER*	2019.06.14		1													

SAMPLE ID	HOST	DATE	*I. ricinus* L	*I. ricinus* N	*I. frontalis* L	*I. frontalis* N	*I. frontalis* F	*I. festai* F	*I. festai* M	*I. lividus* N	*I. lividus* F	*Ha. concinna* L	*Ha. concinna* N	*Ha. punctata* L	*I. arboricola* N	*Hyalomma* sp. N	*D. reticulatus* F
HS373	*ERI RUB*	2019.06.15		1													
HS374	*PHY COL*	2019.06.15		2													
HS375	*ACR SCI*	2019.06.18											1				
HS376	*ACR SCI*	2019.06.18		1													
HS377	*ACR SCI*	2019.06.19		1													
HS378	*LOC LUS*	2019.06.29											3				
HS379	*LOC LUS*	2019.06.29											1				
HS380	*LOC LUS*	2019.06.29											4				
HS381	*LOC LUS*	2019.06.29										1	7				
HS382	*LOC LUS*	2019.06.30											2				
HS383	*TUR PHI*	2019.06.30		3													
HS384	*ACR SCH*	2019.06.30											3				
HS385	*SYL ATR*	2019.06.30		1													
HS386	*TUR PHI*	2019.06.30											1				
HS387	*ACR SCI*	2019.07.06											1				
HS388	*PAR MAJ*	2019.07.07		2													
HS389	*SYL CUR*	2019.07.07											1				
HS390	*LOC LUS*	2019.07.07											3				
HS391	*TUR PHI*	2019.07.07		1									1				
HS392	*ACR RIS*	2019.07.07		1													
HS393	*ACR SCI*	2019.07.10											1				
HS394	*ACR SCI*	2019.07.10											1				
HS396	*LUS MEG*	2019.07.10	3	5									1				
HS401	*ACR SCI*	2019.07.11		2								2	3				
HS405	*LOC LUS*	2019.07.11											1				
HS406	*TUR PHI*	2019.07.12		1													
HS409	*ACR SCI*	2019.07.14											1				
HS413	*ACR SCH*	2019.07.15											1				
HS414	*TUR PHI*	2019.07.15	4	1													
HS416	*LOC LUS*	2019.07.15											1				
HS417	*LOC LUS*	2019.07.16											1				
HS418	*LOC LUS*	2019.07.16										1	7				
HS419	*ACR SCI*	2019.07.17		3													
HS420	*ACR SCI*	2019.07.18	5	1									1				
HS421	*LOC LUS*	2019.07.18											1				
HS422	*PAR MAJ*	2019.07.18	1														
HS423	*ACR SCI*	2019.07.18	1														
HS424	*ACR RIS*	2019.07.21	2														
HS425	*ACR RIS*	2019.07.21		2													
HS426	*ACR SCI*	2019.07.21											4				
HS427	*LOC LUS*	2019.07.22											1				
HS428	*LOC LUS*	2019.07.22											1				
HS429	*LOC LUS*	2019.07.22											1				
HS430	*ACR RIS*	2019.07.22		1													
HS431	*ERI RUB*	2019.07.22	1														
HS432	*LOC LUS*	2019.07.23											7				
HS435	*TUR PHI*	2019.07.24	3	1													
HS436	*LOC LUS*	2019.07.24											3				
HS437	*ACR SCI*	2019.07.24		1								1					
HS438	*ACR SCI*	2019.07.23											1				
HS439	*ACR SCI*	2019.07.24	2														
HS440	*TUR PHI*	2019.07.24	1										1				
HS442	*LUS MEG*	2019.07.25	1	1													
HS443	*ACR SCI*	2019.07.26											1				
HS444	*TUR PHI*	2019.07.26											2				
HS445	*LOC LUS*	2019.07.28										8	2				
HS446	*ACR SCI*	2019.07.28											1				
HS447	*ACR SCI*	2019.08.02											1				

SAMPLE ID	HOST	DATE	*I. ricinus* L	*I. ricinus* N	*I. frontalis* L	*I. frontalis* N	*I. frontalis* F	*I. festai* F	*I. festai* M	*I. lividus* N	*I. lividus* F	*Ha. concinna* L	*Ha. concinna* N	*Ha. punctata* L	*I. arboricola* N	*Hyalomma* sp. N	*D. reticulatus* F
HS448	*LUS MEG*	2019.08.03	1														
HS450	*LOC LUS*	2019.08.03											4				
HS451	*SYL BOR*	2019.08.06										1	1				
HS452	*LOC LUS*	2019.08.06										2	1				
HS453	*ACR SCI*	2019.08.07	2														
HS454	*LOC LUS*	2019.08.07											1				
HS455	*LOC LUS*	2019.08.07											1				
HS456	*ACR SCI*	2019.08.16		1									1				
HS457	*ACR SCI*	2019.08.20	1	1													
HS458	LUS LUS	2019.08.22	4														
HS459	*SYL ATR*	2019.08.22	1														
HS460	*LOC LUS*	2019.08.22											1				
HS461	*ERI RUB*	2019.08.22	1														
HS462	*TUR MER*	2019.08.22		1								1	1				
HS463	*ACR SCI*	2019.08.22				1											
HS464	*TUR PHI*	2019.08.23										1					
HS465	*ERI RUB*	2019.08.23	1														
HS498	*LUS LUS*	2019.08.23	1	4									1				
HS466	*ACR RIS*	2019.08.25		1													
HS467	*SYL COM*	2019.08.25		2													
HS495	*ERI RUB*	2019.09.07	3														
HS497	*LOC LUS*	2019.08.27										2	2				
SH001	*ERI RUB*	2019.09.14	2														
SH002	*ERI RUB*	2019.09.14	1														
SH003	*SYL ATR*	2019.09.14	1														
SH004	*SYL ATR*	2019.09.14		1													
SH005	*ERI RUB*	2019.09.14	1														
SH006	*ERI RUB*	2019.09.14	1														
SH007	*LUS MEG*	2019.09.14		1													
SH008	*ERI RUB*	2019.09.14	1														
SH009	*TUR MER*	2019.09.14	1	5													
SH010	*ERI RUB*	2019.09.14	3														
SH012	*SYL ATR*	2019.09.21		1													
SH013	*ERI RUB*	2019.09.22	2									1					
SH014	*SYL ATR*	2019.09.22										1					
SH015	*ACR ARU*	2019.09.22		2													
SH016	*ERI RUB*	2019.09.29	1														
SH017	*SYL ATR*	2019.09.29		1													
SH018	*ERI RUB*	2019.09.29	1	1													
SH019	*ERI RUB*	2019.09.29	3														
SH020	*ERI RUB*	2019.09.29	4	1													
SH022	*ERI RUB*	2019.10.02	1														
SH024	*ERI RUB*	2019.10.06		1													
SH025	*SYL ATR*	2019.10.07		2													
SH026	*ERI RUB*	2019.10.07		1													
SH027	*ERI RUB*	2019.10.12		1													
SH028	*ERI RUB*	2019.10.12		1													
SH030	*ERI RUB*	2019.10.26		1													
SH031	*ERI RUB*	2019.10.29		1													
SH032	*ERI RUB*	2019.10.29		1													
SH033	*ERI RUB*	2019.10.29		1													
SH034	*ERI RUB*	2019.10.29	1														
SH035	*TUR MER*	2019.10.31	1	1													
SH036	*TUR MER*	2019.11.01		1													
SH037	*ERI RUB*	2019.11.01	1														
SH038	*SIT EUR*	2019.11.01		1													
SH039	*TUR MER*	2019.11.02		1													
SH041	*TUR MER*	2019.11.30		1													

SAMPLE ID	HOST	DATE	*I. ricinus* L	*I. ricinus* N	*I. frontalis* L	*I. frontalis* N	*I. frontalis* F	*I. festai* F	*I. festai* M	*I. lividus* N	*I. lividus* F	*Ha. concinna* L	*Ha. concinna* N	*Ha. punctata* L	*I. arboricola* N	*Hyalomma* sp. N	*D. reticulatus* F
SH042	PAR MAJ	2020.03.09				1											
SH050	ERI RUB	2020.03.17		1													
SH043	ERI RUB	2020.03.17		1													
SH044	TUR MER	2020.04.11		3													
SH045	TUR MER	2020.04.11		1													
SH046	ACR SCI	2020.05.02		1													
SH047	LUS MEG	2020.05.02		2													
SH048	LAN COL	2020.05.09		1													
SH049	LUS MEG	2020.05.10		3													
SH051	TUR MER	2020.05.23		5													
SH052	TUR PHI	2020.05.23		8													
SH053	TUR MER	2020.05.23		3													
SH054	LUS MEG	2020.05.26		2													
SH055	LUS MEG	2020.05.26		4													
SH056	LUS MEG	2020.05.30		1													
SH057	TUR PHI	2020.05.30		1													
SH058	ACR SCI	2020.05.30		1													
SH059	TUR MER	2020.05.30		4													
SH060	TUR PHI	2020.05.30											1				
SH061	TUR MER	2020.05.30		7													
SH062	TUR PHI	2020.05.30		3													
SH063	LUS MEG	2020.05.30		8													
SH064	TUR PHI	2020.05.30		5													
SH065	TUR PHI	2020.05.30		1													
SH067	LOC LUS	2020.05.30											1				
SH071	SYL ATR	2020.06.06		2													
SH072	PAR MAJ	2020.06.06		1													
SH073	SYL ATR	2020.06.06		4													
SH074	ACR SCH	2020.06.06		1													
SH075	TUR MER	2020.06.06		5													
SH078	TUR PHI	2020.06.06		3													
SH079	SYL ATR	2020.06.06		1													
SH080	PAR MAJ	2020.06.06		1													
SH081	TUR PHI	2020.06.06		1													
SH082	TUR PHI	2020.06.06		4													
SH084	TUR PHI	2020.06.27		1													
SH085	TUR MER	2020.06.27		2													
SH086	LUS MEG	2020.06.27		1													
SH087	LOC LUS	2020.06.28										1					
SH088	LOC LUS	2020.06.28											1				
SH091	LOC LUS	2020.07.10											1				
SH093	ACR SCH	2020.07.10										1	1				
SH094	LOC LUS	2020.07.10											1				
SH095	TUR PHI	2020.07.10											1				
SH096	TUR MER	2020.07.10	2	3								13	1				
SH097	TUR PHI	2020.07.10											6				
SH098	TUR MER	2020.07.10		3													
SH099	SYL ATR	2020.07.10		1													
SH101	LOC LUS	2020.07.10											2				
SH102	ERI RUB	2020.07.10	5														
SH103	ERI RUB	2020.07.10	4														
SH104	LOC LUS	2020.07.10	1	2													
SH105	TUR MER	2020.07.10	1	1									2				
SH106	LOC LUS	2020.07.11										1					
SH107	LOC LUS	2020.07.11										9	1				
SH108	ACR SCH	2020.07.11											1				
SH109	LOC LUS	2020.07.11											2				
SH110	LOC LUS	2020.07.11										1	1				

SAMPLE ID	HOST	DATE	*I. ricinus* L	*I. ricinus* N	*I. frontalis* L	*I. frontalis* N	*I. frontalis* F	*I. festai* F	*I. festai* M	*I. lividus* N	*I. lividus* F	*Ha. concinna* L	*Ha. concinna* N	*Ha. punctata* L	*I. arboricola* N	*Hyalomma* sp. N	*D. reticulatus* F
SH111	EMB SCH	2020.07.11											7				
SH112	LOC LUS	2020.07.11											1				
SH113	SYL ATR	2020.07.11		1													
SH114	TUR PHI	2020.07.11										1	1				
SH115	TUR PHI	2020.07.11											2				
SH116	ACR SCI	2020.07.11											1				
SH117	LOC LUS	2020.07.11											1				
SH118	ACR SCH	2020.07.11											1				
SH119	ACR SCI	2020.07.12		1													
SH121	LOC LUS	2020.07.12										3	1				
SH122	TUR PHI	2020.07.16	9	7									1				
SH123	ACR SCI	2020.07.16										8	1				
SH124	ACR SCI	2020.07.16	2										1				
SH125	ACR SCI	2020.07.16	6	2								4					
SH126	LOC LUS	2020.07.17										1	7				
SH127	TUR MER	2020.07.17	1														
SH128	ACR SCI	2020.07.17	1														
SH143	ACR RIS	2020.07.26		2													
SH144	ACR SCI	2020.07.26											1				
SH145	ACR SCI	2020.07.26											1				
SH146	ACR SCH	2020.07.26											2				
SH147	TUR PHI	2020.07.26		1									2				
SH148	ACR SCI	2020.07.26											1				
SH149	LUS MEG	2020.07.27	5	1													
SH152	LOC LUS	2020.08.01											2				
SH153	LOC LUS	2020.08.01											1				
SH154	LUS MEG	2020.08.01											2				
SH155	PHY COL	2020.08.01		1													
SH156	LOC LUS	2020.08.01											1				
SH157	LOC LUS	2020.08.01											2				
SH158	ACR ARU	2020.08.08	2	1													
SH159	LUS LUS	2020.08.08	4														
SH160	SYL ATR	2020.08.08		1													
SH161	TUR MER	2020.08.08	1														
SH162	ACR SCI	2020.08.08		2													
SH163	ACR RIS	2020.08.08	1	1													
SH164	TUR MER	2020.07.30	6	1													
SH166	LUS MEG	2020.08.09	1														
SH167	LUS MEG	2020.08.09	7									1					
SH168	LUS MEG	2020.08.09		1													
SH169	ACR SCI	2020.08.11	3														
SH170	ACR RIS	2020.08.12	4														
SH171	LUS MEG	2020.08.12	2														
SH172	LUS MEG	2020.08.12	3														
SH173	LUS LUS	2020.08.13	1														
SH174	ACR RIS	2020.08.13		1													
SH176	TUR PHI	2020.08.29	2														
SH177	ERI RUB	2020.08.29	5														
SH178	TUR PHI	2020.08.29	3														
SH179	TUR PHI	2020.08.29	1	1								2					
SH180	ERI RUB	2020.08.29	3														
SH181	LUS LUS	2020.08.29	1	1													
SH182	LOC LUS	2020.08.29										1	1				
SH183	ACR SCI	2020.08.29											1				
SH184	ACR SCI	2020.08.29		1													
SH199	ACR ARU	2020.09.04	1														
SH200	SYL BOR	2020.09.04		1													
SH185	SYL ATR	2020.09.04	1														

SAMPLE ID	HOST	DATE	*I. ricinus* L	*I. ricinus* N	*I. frontalis* L	*I. frontalis* N	*I. frontalis* F	*I. festai* F	*I. festai* M	*I. lividus* N	*I. lividus* F	*Ha. concinna* L	*Ha. concinna* N	*Ha. punctata* L	*I. arboricola* N	*Hyalomma* sp. N	*D. reticulatus* F
SH186	SYL ATR	2020.09.04	1	1													
SH187	SYL ATR	2020.09.04	1														
SH188	TUR PHI	2020.09.05	2														
SH189	LUS MEG	2020.09.05	3														
SH191	LOC LUS	2020.09.05										7					
SH194	TUR MER	2020.09.05		1													
SH195	SYL ATR	2020.09.05	1														
SH196	SYL ATR	2020.09.06		1													
SH197	SYL ATR	2020.09.06		1													
SH198	SYL ATR	2020.09.11	2														
OC01	ACR RIS	2020.09.12	1	1													
OC02	SYL ATR	2020.09.12	2														
OC03	LUS MEG	2020.09.12	2									3					
OC04	ERI RUB	2020.09.12		1													
OC05	PAR CAE	2020.09.12		1													
OC06	SYL ATR	2020.09.12		1													
OC07	ERI RUB	2020.09.12	2														
OC08	SYL ATR	2020.09.12		1													
OC09	SYL BOR	2020.09.13		1													
OC10	ANT TRI	2020.09.13	1	1													
OC12	ERI RUB	2020.09.20	1														
OC13	ERI RUB	2020.09.23	1														
OC14	ERI RUB	2020.09.25	1														
OC15	ERI RUB	2020.09.25	4														
OC16	ERI RUB	2020.09.27	2														
OC17	ERI RUB	2020.09.27	1														
OC18	ERI RUB	2020.09.27	1														
OC19	ERI RUB	2020.09.27		1													
OC20	ERI RUB	2020.09.30		1													
OC21	SYL ATR	2020.10.02		1													
OC22	ERI RUB	2020.10.04				1											
OC24	SYL ATR	2020.10.07		1													
OC25	TUR MER	2020.10.10		2													
OC26	TUR MER	2020.10.10		1													
OC27	ERI RUB	2020.10.10		1													
OC28	PHY COL	2020.10.14		1													
OC29	ERI RUB	2020.10.16		1													
OC30	ERI RUB	2020.10.18		1													
OC31	ERI RUB	2020.10.18	1														
OC32	FRI COE	2020.10.19		1													
OC33	ERI RUB	2020.10.22		1													
OC35	TUR MER	2020.11.03		1													
OC36	TRO TRO	2020.11.07	1														
OC37	ERI RUB	2021.03.13		1													
OC38	TUR PHI	2021.03.15				1											
OC39	TUR MER	2021.03.15		1													
OC40	TUR PHI	2021.03.15		2													
OC41	TUR MER	2021.03.15				1											
OC42	TUR MER	2021.03.20		1													
OC43	TUR PHI	2021.03.20		1													
OC44	TUR PHI	2021.03.20		2													
OC45	ERI RUB	2021.03.29					1										
OC46	ERI RUB	2021.03.30		1													
OC47	ERI RUB	2021.03.30		1													
OC48	PRU MOD	2021.03.31		1													
OC49	PRU MOD	2021.03.31					1										
OC50	PRU MOD	2021.04.01		2				2									
OC51	ERI RUB	2021.04.01		1													

SAMPLE ID	HOST	DATE	*I. ricinus* L	*I. ricinus* N	*I. frontalis* L	*I. frontalis* N	*I. frontalis* F	*I. festai* F	*I. festai* M	*I. lividus* N	*I. lividus* F	*Ha. concinna* L	*Ha. concinna* N	*Ha. punctata* L	*I. arboricola* N	*Hyalomma* sp. N	*D. reticulatus* F
OC52	PRU MOD	2021.04.03		3													
OC53	ERI RUB	2021.04.04		1													
OC54	ERI RUB	2021.04.10		1													
OC55	ERI RUB	2021.04.10		2													
OC56	ERI RUB	2021.04.10		1													
OC57	LUS MEG	2021.04.10		3													
OC58	TUR MER	2021.04.10		6													
OC59	ERI RUB	2021.04.10		1													
OC60	TUR PHI	2021.04.10		4													
OC61	TUR MER	2021.04.10		1													
OC62	ERI RUB	2021.04.10	1	3													
OC63	TUR MER	2021.04.10		3													
OC64	PAR MAJ	2021.05.01		1													
OC65	ACR SCI	2021.05.01		1													
OC66	ACR SCI	2021.05.01		1													
OC67	TUR PHI	2021.05.01		6													
OC69	SYL COM	2021.05.08		5													
OC70	PHY COL	2021.05.08		1													
OC71	TUR MER	2021.05.08		12													
OC72	TUR MER	2021.05.08		4													
OC73	TUR MER	2021.05.15		2													
OC75	TUR PHI	2021.05.16		3													
OC76	TUR PHI	2021.05.16		1													
OC77	TUR MER	2021.05.16		1													
OC78	ACR SCI	2021.05.16		1													
OC80	TUR MER	2021.05.16		1													
OC81	ACR SCI	2021.05.29		1													
OC82	LOC LUS	2021.05.29		1													
OC83	TUR MER	2021.05.29		3													
OC84	TUR MER	2021.05.29		4													
OC85	TUR MER	2021.05.29		8													
OC86	TUR MER	2021.05.30	2	8									3				
OC87	LUS MEG	2021.06.05		1													
OC88	ACR SCI	2021.06.19		3													
OC89	PHY COL	2021.06.19		1													
OC90	SYL ATR	2021.06.19		1													
OC91	SYL ATR	2021.06.19		2													
OC92	SYL ATR	2021.06.19		1													
OC93	SYL ATR	2021.06.19		2													
OC94	PAR MAJ	2021.06.19		1													
OC95	ERI RUB	2021.07.03		1													
OC96	LUS MEG	2021.07.10		1													
OC97	SYL ATR	2021.07.10		1													
OC98	ACR SCH	2021.07.10											1				
OC99	ACR RIS	2021.07.11		1													
OC100	ERI RUB	2021.07.11	1										1				
OC101	LOC LUS	2021.07.18											2				
OC102	LOC LUS	2021.07.18											1				
OC103	POR ANA	2021.07.23		1													
OC104	TUR MER	2021.07.24											1				
OC105	TUR PHI	2021.07.24	2	1									2				
OC106	ACR ARU	2021.07.24											1				
OC107	ACR SCI	2021.07.24		1													
OC109	ACR RIS	2021.07.24		1									1				
OC111	ACR ARU	2021.07.24										1	1				
OC112	ACR SCI	2021.07.24											1				
OC113	LOC LUS	2021.07.24											2				
OC114	LOC FLU	2021.07.24										1	2				

SAMPLE ID	HOST	DATE	*I. ricinus* L	*I. ricinus* N	*I. frontalis* L	*I. frontalis* N	*I. frontalis* F	*I. festai* F	*I. festai* M	*I. lividus* N	*I. lividus* F	*Ha. concinna* L	*Ha. concinna* N	*Ha. punctata* L	*I. arboricola* N	*Hyalomma* sp. N	*D. reticulatus* F
OC115	LOC LUS	2021.07.28											1				
OC116	LOC LUS	2021.07.28											2				
OC117	LOC LUS	2021.07.28											1				
OC118	LOC LUS	2021.07.28											4				
OC119	ACR RIS	2021.07.31	2														
OC120	ACR SCI	2021.07.31											1				
OC122	LOC LUS	2021.07.31											3				
OC123	PAR MAJ	2021.07.31	8	5													
OC124	PAR MAJ	2021.07.31	3	2													
OC125	LOC LUS	2021.08.07											1				
OC126	LOC LUS	2021.08.07											1				
OC127	TUR PHI		4										2				
OC128	TUR PHI	2021.08.07	2	2													
OC129	LOC LUS	2021.08.07											2				
OC130	SYL ATR	2021.08.07											1				
OC131	LOC LUS	2021.08.07											1				
OC132	ACR SCH	2021.08.07											1				
OC133	LOC LUS	2021.08.07		1									4				
OC135	TUR PHI	2021.08.10	2	2													
OC136	LOC LUS	2021.08.11										1	4				
OC137	LOC LUS	2021.08.12	2														
OC138	LOC LUS	2021.08.12											1				
OC139	ACR SCH	2021.08.12										1	4				
OC140	SYL ATR	2021.08.14	1														
OC141	LUS LUS	2021.08.14	2														
OC142	ACR RIS	2021.08.14		1													
OC143	LOC LUS	2021.08.14										5	3				
OC144	LOC LUS	2021.08.14										8	3				
OC145	LUS MEG	2021.08.14		1													
OC146	SYL ATR	2021.08.14		2													
OC147	SYL ATR	2021.08.14										1					
OC148	SYL COM	2021.08.14		2													
OC149	LOC LUS	2021.08.14										5	4				
OC151	ACR SCI	2021.08.14		1													
OC152	SYL COM	2021.08.15	1	1													
OC153	ACR SCI	2021.08.15		2													
OC155	SYL BOR	2021.08.20		1													
OC156	ACR SCI	2021.08.21		1													
OC157	LUS LUS	2021.08.21	1														
OC158	LUS MEG	2021.08.21		1													
OC159	ERI RUB	2021.08.22	4														
OC160	LUS MEG	2021.08.22	4										1				
OC162	ACR SCI	2021.08.26											1				
OC164	ERI RUB	2021.08.28	1														
OC165	SYL BOR	2021.08.28		1													
OC166	SYL ATR	2021.08.28	1														
OC167	SYL ATR	2021.08.28										1					
OC169	SYL BOR	2021.08.29		1													
OC170	LUS MEG	2021.09.04	1														
OC171	LUS LUS	2021.09.04		1													
OC173	SYL ATR	2021.09.04	1														
OC174	SYL BOR	2021.09.04		1													
OC176	ACR SCH	2021.09.04		1													
OC177	SYL ATR	2021.09.04	4														
OC178	ERI RUB	2021.09.04	2	1													
OC179	TUR PHI	2021.09.04		1													
OC181	SYL ATR	2021.09.04	1														
OC183	SYL ATR	2021.09.11	1														

SAMPLE ID	HOST	DATE	*I. ricinus* L	*I. ricinus* N	*I. frontalis* L	*I. frontalis* N	*I. frontalis* F	*I. festai* F	*I. festai* M	*I. lividus* N	*I. lividus* F	*Ha. concinna* L	*Ha. concinna* N	*Ha. punctata* L	*I. arboricola* N	*Hyalomma* sp. N	*D. reticulatus* F
OC184	SYL ATR	2021.09.11	1														
OC185	SYL ATR	2021.09.11	1														
OC186	PHY TRO	2021.09.11		1													
OC187	SYL ATR	2021.09.11	1														
OC188	SYL ATR	2021.09.11	1														
OC189	ERI RUB	2021.09.11	1	1													
OC190	ERI RUB	2021.09.11		1													
OC191	ERI RUB	2021.09.12	1														
OC192	ERI RUB	2021.09.19		1													
OC193	PHY TRO	2021.09.19		1													
OC194	SIT EUR	2021.09.19		1													
OC195	ERI RUB	2021.09.25		3													
OC196	SYL ATR	2021.09.25		1													
OC197	ERI RUB	2021.09.25	1	1													
OC198	ERI RUB	2021.09.25		1													
OC199	ERI RUB	2021.09.26		1													
OC200	SYL ATR	2021.09.26		1													
KG1	ERI RUB	2021.09.26		2													
KG2	ERI RUB	2021.09.26		1													
KG3	ERI RUB	2021.09.26	3	2													
KG4	ERI RUB	2021.10.10		1													
KG5	ERI RUB	2021.10.16		1													
KG6	TUR MER	2021.10.16		1													
KG7	TUR MER	2021.10.16		2													
KG8	TUR MER	2021.10.16		1													
KG10	ERI RUB	2021.10.16		1													
KG11	ERI RUB	2021.10.23	1														
KG12	TUR MER	2021.10.24		3													
KG13	ERI RUB	2021.10.24	1	1													
KG14	ERI RUB	2021.10.26		1													
KG15	ERI RUB	2021.10.26		1													
KG16	ERI RUB	2021.10.27		4													
KG17	ERI RUB	2021.10.28		1													
KG18	PAR MAJ	2021.10.29		1													
KG19	TUR ILI	2021.10.30		4													
KG20	ERI RUB	2021.10.30		1													
KG21	PAR MAJ	2021.11.13					1										
KG22	TUR MER	2022.03.18		2													
KG23	TUR MER	2022.03.18		11													
KG24	PAR MAJ	2022.03.19		1													
KG25	PAR MAJ	2022.03.19		1													
KG26	PAR MAJ	2022.03.19		2													
KG27	TUR MER	2022.03.19		2													
KG28	COC COC	2022.03.22		1													
KG29	TUR PHI	2022.03.24		1													
KG30	ERI RUB	2022.03.25		1													
KG31	TUR MER	2022.03.25		1													1
KG32	ERI RUB	2022.04.04		1													
KG34	TUR MER	2022.04.10		5													
KG35	TUR MER	2022.04.10		8													
KG36	ERI RUB	2022.04.10		5													
KG37	TRO TRO	2022.04.11		1													
KG38	TRO TRO	2022.04.11		1													
KG39	ERI RUB	2022.04.12		2													
KG40	ERI RUB	2022.04.13		2													
KG41	TUR PHI	2022.04.13		2													
KG42	ERI RUB	2022.04.16		3													
KG43	TUR MER	2022.04.16		3													

SAMPLE ID	HOST	DATE	*I. ricinus* L	*I. ricinus* N	*I. frontalis* L	*I. frontalis* N	*I. frontalis* F	*I. festai* F	*I. festai* M	*I. lividus* N	*I. lividus* F	*Ha. concinna* L	*Ha. concinna* N	*Ha. punctata* L	*I. arboricola* N	*Hyalomma* sp. N	*D. reticulatus* F
KG44	TUR MER	2022.04.16		1													
KG45	TRO TRO	2022.04.16		2													
KG46	TUR MER	2022.04.16		3													
KG47	ACR SCH	2022.04.16		1													
KG48	TUR MER	2022.04.23		1													
KG49	TUR MER	2022.04.23		1													
KG50	SYL ATR	2022.04.23		1													
KG51	ACR SCH	2022.04.23		3													
KG52	SYL COM	2022.04.23		1													
KG53	ACR SCH	2022.04.23											1				
KG54	ACR SCI	2022.04.23				1											
KG55	ACR SCI	2022.04.30		1													
KG56	PHY COL	2022.05.07		1													
KG57	LUS MEG	2022.05.07		2													
KG58	PHY COL	2022.05.07		1													
KG59	LOC LUS	2022.05.07											1				
KG60	LUS MEG	2022.05.07	1	2													
KG61	TUR MER	2022.05.15	1	9													
KG62	TUR PHI	2022.05.15		1													
KG63	SYL ATR	2022.05.15		1													
KG65	PAR MAJ	2022.05.28		1													
KG66	TUR PHI	2022.06.11		1													
KG67	SIT EUR	2022.06.11		1													
KG68	TUR PHI	2022.06.11		1									1				
KG69	LUS MEG	2022.06.12		2													
KG70	SYL ATR	2022.06.18		1													
KG71	SYL ATR	2022.06.18		6													
KG72	LOC LUS	2022.06.18		1								2	5				
KG73	TRO TRO	2022.06.18		1													
KG74	TUR PHI	2022.06.18		3									1				
KG75	LOC LUS	2022.06.18										17	6				
KG76	LOC LUS	2022.06.18		1								21	6				
KG77	ACR RIS	2022.07.08		1													
KG78	ACR SCI	2022.07.08		1													
KG79	LOC LUS	2022.07.08											1				
KG80	LOC LUS	2022.07.08										1	2				
KG81	LOC LUS	2022.07.08										6	3				
KG82	TUR MER	2022.07.08		1													
KG83	LOC LUS	2022.07.08											1				
KG84	LOC LUS	2022.07.08											2				
KG85	ACR SCH	2022.07.08											1				
KG86	LOC LUS	2022.07.10											1				
KG87	ACR SCI	2022.07.10											1				
KG88	LOC LUS	2022.07.10											2				
KG89	LOC LUS	2022.07.10										5					
KG90	LOC LUS	2022.07.10											1				
KG93	ACR SCI	2022.07.11											1				
KG94	ACR SCH	2022.07.11											2				
KG95	LOC LUS	2022.07.13											2				
KG96	ACR SCH	2022.07.13											1				
KG97	ACR SCH	2022.07.13											2				
KG98	TUR MER	2022.07.16											1				
KG99	ACR MEL	2022.07.16		2									1				
KG100	TUR PHI	2022.07.16		1													
KG101	LOC LUS	2022.07.16		1									1				
KG102	ACR RIS	2022.07.16											1				
KG103	ACR MEL	2022.07.16										1	1				
KG104	ACR SCI	2022.07.16		1													

SAMPLE ID	HOST	DATE	*I. ricinus* L	*I. ricinus* N	*I. frontalis* L	*I. frontalis* N	*I. frontalis* F	*I. festai* F	*I. festai* M	*I. lividus* N	*I. lividus* F	*Ha. concinna* L	*Ha. concinna* N	*Ha. punctata* L	*I. arboricola* N	*Hyalomma* sp. N	*D. reticulatus* F
KG109	ACR RIS	2022.07.19											1				
KG110	ACR SCI	2022.07.20		1													
KG111	ACR RIS	2022.07.20		1													
KG112	ACR SCH	2022.07.23											1				
KG113	ACR RIS	2022.07.23		2													
KG114	ACR RIS	2022.07.23		1									2				
KG115	LOC LUS	2022.07.23										2	3				
KG116	LOC LUS	2022.07.25		1									4				
KG117	LUS MEG	2022.07.26											1				
KG118	ACR RIS	2022.07.30											1				
KG121	LOC LUS	2022.08.06											1				
KG122	SYL ATR	2022.08.06	1														
KG124	SYL COM	2022.08.06	1														
KG125	ACR SCI	2022.08.06										1	2				
KG127	SYL BOR	2022.08.14		1													
KG128	ACR SCI	2022.08.14											1				
KG129	LUS LUS	2022.08.14		2													
KG130	ERI RUB	2022.08.15		1													
KG131	ERI RUB	2022.08.17	1														
KG132	LUS LUS	2022.08.17	1														
KG133	SYL ATR	2022.08.17		1													
KG134	LUS LUS	2022.08.18	1														
KG135	SYL ATR	2022.08.18	3	1													
KG136	LUS LUS	2022.08.18	1														
KG137	LUS LUS	2022.08.18		4													
KG138	ERI RUB	2022.08.19		1													
KG139	LUS MEG	2022.08.19	2	1								1					
KG140	LUS LUS	2022.08.22	1														
KG141	SYL BOR	2022.08.22		1													
KG142	LUS LUS	2022.08.23	2	1													
KG143	ERI RUB	2022.08.23	1														
KG145	LUS MEG	2022.08.23		1													
KG148	ACR SCI	2022.08.24											1				
KG152	ACR RIS	2022.08.25		1													
KG153	ERI RUB	2022.08.25	1														
KG155	ACR RIS	2022.08.25		1													
KG156	ERI RUB	2022.08.25	2														
KG157	SYL COM	2022.08.25		1													
KG159	SYL COM	2022.08.26		1													
KG160	LUS MEG	2022.08.26	1														
KG161	SYL COM	2022.08.26		1													
KG162	SYL ATR	2022.08.26	1														
KG163	LUS MEG	2022.08.26	2														
KG164	SYL ATR	2022.08.26	2	1													
KG165	LUS MEG	2022.08.27	1														
KG166	ACR SCI	2022.08.27		1													
KG167	SYL ATR	2022.08.28	1														
KG170	SYL COM	2022.09.03		1													
KG173	ERI RUB	2022.09.08		1													
KG174	ACR RIS	2022.09.11		1													
KG175	TUR MER	2022.09.16	1	1													
KG176	SYL ATR	2022.09.23		1													
KG177	ERI RUB	2022.09.23		1													
KG178	ACR SCH	2022.09.24		1													
KG179	ERI RUB	2022.09.25	1	1													
KG180	TUR PHI	2022.09.25		1													
KG181	SYL ATR	2022.09.25		1													
KG182	CER BRA	2022.09.25		1													

SAMPLE ID	HOST	DATE	*I. ricinus* L	*I. ricinus* N	*I. frontalis* L	*I. frontalis* N	*I. frontalis* F	*I. festai* F	*I. festai* M	*I. lividus* N	*I. lividus* F	*Ha. concinna* L	*Ha. concinna* N	*Ha. punctata* L	*I. arboricola* N	*Hyalomma* sp. N	*D. reticulatus* F
KG183	ERI RUB	2022.10.03		1													
KG184	TUR PHI	2022.10.08	1			1						1					
KG185	ERI RUB	2022.10.08	1														
KG186	ERI RUB	2022.10.08		1													
KG187	FRI COE	2022.10.08		1													
KG188	ERI RUB	2022.10.21	2	1													
KG189	ERI RUB	2022.10.22	1														
KG190	TUR MER	2022.10.22		1													
KG191	PAR MAJ	2022.10.22		1													
KG192	TUR MER	2022.10.22		1													
KG193	TUR MER	2022.10.22		3													
KG194	ERI RUB	2022.10.22		1													
KG195	ERI RUB	2022.10.22		3													
KG301	PAS MON	2022.10.27					1										
KG302	ERI RUB	2022.10.29		1													
KG303	TUR MER	2022.10.29		3													
KG304	TUR MER	2022.10.29	1	1													
KG305	TUR MER	2022.11.12		1													
		**TOTAL:**	**1229**	**2742**	**52**	**38**	**12**	**6**	**2**	**1**	**12**	**698**	**1008**	**28**	**1**	**3**	**1**
