# Supplementary material for: Long term evaluation of factors influencing the association of ixodid ticks with birds in Central Europe, Hungary
Source: Sci Rep. 2024 Feb 29;14:4958. doi: 10.1038/s41598-024-55021-9 (PMC10902401; doi:10.1038/s41598-024-55021-9)
Supplement: Supplementary file 7 — Supplementary Table 2. [file 41598_2024_55021_MOESM7_ESM.pdf]

Supplementary Table 2 - Bird species according to their migration habits, with the total number of ticks found on them

		I. ricinus	*I. frontalis*	*I. arboricola*	*I. lividus*	*I. festai*	*Ha. concinna*	*Ha. punctata*	*Hy. marginatum*	*Hy. rufipes*	*D. reticulatus*	Number of tick species	Total number of ticks	Total number of infested birds
LDM	ACR ARU	26	0	0	0	0	15	0	0	0	0	2	41	27
LDM	ACR RIS	158	0	0	0	0	72	0	0	0	0	2	230	118
LDM	ACR SCH	13	0	0	0	0	135	0	0	0	0	2	148	54
LDM	ACR SCI	138	2	0	0	0	157	0	0	0	0	3	297	167
LDM	ANT TRI	13	0	0	0	0	0	0	0	0	0	1	13	8
LDM	FIC ALB	0	0	0	0	0	0	0	0	1	0	1	1	1
LDM	FIC HYP	2	0	0	0	0	0	0	0	0	0	1	2	1
LDM	HYP ICT	3	0	0	0	0	0	0	0	0	0	1	3	3
LDM	LAN COL	12	0	0	0	0	1	0	0	0	0	2	13	6
LDM	LOC FLU	1	0	0	0	0	56	0	0	0	0	2	57	9
LDM	LOC LUS	19	0	0	0	0	933	0	0	0	0	2	952	243
LDM	LUS LUS	114	0	0	0	0	1	0	0	0	0	2	115	35
LDM	LUS MEG	187	0	0	0	0	30	0	0	0	0	2	217	95
LDM	LUS SVE	1	0	0	0	0	0	0	0	0	0	1	1	1
LDM	PHO PHO	5	0	0	0	0	0	0	0	0	0	1	5	2
LDM	PHY TRO	11	0	0	0	0	0	0	0	0	0	1	11	9
LDM	RIP RIP	0	0	0	13	0	0	0	0	0	0	1	13	4
LDM	SYL BOR	16	0	0	0	0	3	0	0	0	0	2	19	16
LDM	SYL COM	43	0	0	0	0	1	0	0	0	0	2	44	28
LDM	SYL CUR	3	0	0	0	0	1	0	0	0	0	2	4	4
LDM	SYL NIS	2	0	0	0	0	1	0	0	0	0	2	3	2
MDM	FRI MON	5	0	0	0	0	0	0	0	0	0	1	5	4
R	PAR CAE	5	0	0	0	0	0	0	0	0	0	1	5	5
R	PAR MAJ	98	5	1	0	0	0	0	0	0	0	3	104	63
R	PAS MON	2	1	0	0	0	1	0	0	0	0	3	4	3
R	REM PEN	0	1	0	0	0	0	0	0	0	0	1	1	1
R	SIT EUR	7	0	0	0	0	0	0	0	0	0	1	7	6
R/MDM	CAR CHL	27	1	0	0	0	2	0	0	0	0	3	30	20
R/MDM	COC COC	46	1	0	0	0	0	0	0	0	0	2	47	12
R/SDM	AEG CAU	1	0	0	0	0	0	0	0	0	0	1	1	1
R/SDM	CER BRA	3	0	0	0	0	0	0	0	0	0	1	3	3
R/SDM	EMB CIT	0	0	0	0	0	2	0	0	0	0	1	2	1
R/SDM	EMB SCH	1	0	0	0	0	15	0	0	0	0	2	16	4
R/SDM	ERI RUB	924	52	0	0	0	23	0	1	0	0	4	1000	521
R/SDM	FRI COE	25	1	0	0	0	0	0	0	0	0	2	26	18
R/SDM	GAR GLA	8	0	0	0	0	0	0	0	0	0	1	8	3
R/SDM	RAL AQU	0	0	0	0	0	1	0	0	0	0	1	1	1
R/SDM	REG REG	1	0	0	0	0	0	0	0	0	0	1	1	1
R/SDM	TUR MER	1073	18	0	0	5	129	0	0	0	1	5	1226	359
SDM	ACR MEL	2	0	0	0	0	5	0	0	0	0	2	7	4
SDM	COT COT	1	0	0	0	0	2	28	0	0	0	3	31	3
SDM	PHY COL	46	2	0	0	0	2	0	0	0	0	3	50	31
SDM	POR ANA	1	0	0	0	0	0	0	0	0	0	1	1	1
SDM	PRU MOD	262	11	0	0	3	3	0	0	0	0	4	279	90
SDM	PYR PYR	2	0	0	0	0	0	0	0	0	0	1	2	2
SDM	STU VUL	0	0	0	0	0	1	0	0	0	0	1	1	1
SDM	SYL ATR	277	1	0	0	0	12	0	0	0	0	3	290	199
SDM	TRO TRO	50	0	0	0	0	0	0	0	0	0	1	50	32
SDM	TUR ILI	25	0	0	0	0	0	0	0	0	0	1	25	4
SDM	TUR PHI	310	6	0	0	0	102	0	1	0	0	4	419	168
SDM	TUR TOR	2	0	0	0	0	0	0	0	0	0	1	2	1

R: resident, **SDM**: short-distance migrants, **MDM**: middle distance migrants, **R/SDM**: resident or short distance migrants, **R/MDM**: residents ot middle distance migrants, **LDM**: long-distance migrants
