# Supplementary material for: Long term evaluation of factors influencing the association of ixodid ticks with birds in Central Europe, Hungary
Source: Sci Rep. 2024 Feb 29;14:4958. doi: 10.1038/s41598-024-55021-9 (PMC10902401; doi:10.1038/s41598-024-55021-9)
Supplement: Supplementary file 8 — Supplementary Table 3. [file 41598_2024_55021_MOESM8_ESM.pdf]

Supplementary Table 3: Categorization of bird species according to their migration habit, habitat type, feeding place and weight

Bird names			Migration habit	Habitat type	Feeding place	Weight (g)
**English name**	**Scientific name**	**HURING code**	**Resident (R), Short (SDM), Middle (MDM), Long-distance migrant (LDM)**	**Forest (F), Meadow (M), Reed (R)**	**Ground level (G) or Above ground level (A)**	**min - max**
Great Reed Warbler	*Acrocephalus arundinaceus*	ACR ARU	LDM	R	A	24-40
Moustached Warbler	*Acrocephalus melanopogon*	ACR MEL	SDM	R	A	10-14
Marsh Warbler	*Acrocephalus palustris*	ACR RIS	LDM	R	A	10-14
Sedge Warbler	*Acrocephalus schoenobaenus*	ACR SCH	LDM	R	A	9-15
Eurasian Reed Warbler	*Acrocephalus scirpaceus*	ACR SCI	LDM	R	A	10-16
Long-tailed Tit	*Aegithalos caudatus*	AEG CAU	R/SDM	F	A	7-10
Tree Pipit	*Anthus trivialis*	ANT TRI	LDM	F/M	G/A	18-29
European Greenfinch	*Chloris chloris*	CAR CHL	R/MDM	F	G	24-31
Short-toed Treecreeper	*Certhia brachydactyla*	CER BRA	R/SDM	F	A	8-11
Hawfinch	*Coccothraustes coccothraustes*	COC COC	R/MDM	F	A	46-80
Common Quail	*Coturnix coturnix*	COT COT	SDM	M	G	75-135
Yellowhammer	*Emberiza citrinella*	EMB CIT	R/SDM	M	A	25-36
Common Reed Bunting	*Emberiza schoeniclus*	EMB SCH	R/SDM	R	A	16-25
European Robin	*Erithacus rubecula*	ERI RUB	R/SDM	F	G	14-21
Collared Flycatcher	*Ficedula albicollis*	FIC ALB	LDM	F	A	12-16
European Pied Flycatcher	*Ficedula hypoleuca*	FIC HYP	LDM	F	A	10-15
Common Chaffinch	*Fringilla coelebs*	FRI COE	R/SDM	F	A	18-29
Brambling	*Fringilla montifringilla*	FRI MON	MDM	F	A	17-30
Eurasian Jay	*Garrulus glandarius*	GAR GLA	R/SDM	F	G/A	140-190
Icterine Warbler	*Hippolais icterina*	HYP ICT	LDM	F	A	10-15
Bluethroat	*Luscinia svecica*	LUS SVE	LDM	R	G	15-25
Red-backed Shrike	*Lanius collurio*	LAN COL	LDM	M	G	25-35
River Warbler	*Locustella fluviatilis*	LOC FLU	LDM	F	G	15-19
Savi’s Warbler	*Locustella luscinioides*	LOC LUS	LDM	R	A	14-17
Thrush Nightingale	*Luscinia luscinia*	LUS LUS	LDM	F	G	24-30
Common Nightingale	*Luscinia megarhynchos*	LUS MEG	LDM	F	G	17-24
Eurasian Blue Tit	*Cyanistes caeruleus*	PAR CAE	R	F	A	9-13
Great Tit	*Parus major*	PAR MAJ	R	F	A	14-22
Eurasian Tree Sparrow	*Passer montanus*	PAS MON	R	M	G	18-29
Common Redstart	*Phoenicurus phoenicurus*	PHO PHO	LDM	F	G	11-19
Common Chiffchaff	*Phylloscopus collybita*	PHY COL	SDM	F	A	6-10
Willow Warbler	*Phylloscopus trochilus*	PHY TRO	LDM	F	A	7-12
Spotted Crake	*Porzana porzana*	POR ANA	SDM	R	A	70-110
Dunnock	*Prunella modularis*	PRU MOD	SDM	F	A	16-25
Eurasian Bullfinch	*Pyrrhula pyrrhula*	PYR PYR	SDM	F	A	16-38
Water Rail	*Rallus aquaticus*	RAL AQU	R/SDM	R	G	80-180
Goldcrest	*Regulus regulus*	REG REG	R/SDM	F	A	4-7
Eurasian Penduline Tit	*Remiz pendulinus*	REM PEN	R	R	A	8-11
Sand Martin	*Riparia riparia*	RIP RIP	LDM	sand walls	only flying insects	11-16
Eurasian Nuthatch	*Sitta europaea*	SIT EUR	R	F	A	21-26
Common Starling	*Sturnus vulgaris*	STU VUL	SDM	F	A	60-90
Eurasian Blackcap	*Sylvia atricapilla*	SYL ATR	SDM	F	A	16-25
Garden Warbler	*Sylvia borin*	SYL BOR	LDM	F	A	16-22
Common Whitethroat	*Curruca communis*	SYL COM	LDM	M	A	13-18
Lesser Whitethroat	*Curruca curruca*	SYL CUR	LDM	M	A	10-14
Barred Warbler	*Curruca nisoria*	SYL NIS	LDM	M	A	22-28
Winter Wren	*Troglodytes troglodytes*	TRO TRO	SDM	F	G	7-12
Redwing	*Turdus iliacus*	TUR ILI	SDM	F	G	50-75
Common Blackbird	*Turdus merula*	TUR MER	R/SDM	F/M	G	80-125
Song Thrush	*Turdus philomelos*	TUR PHI	SDM	F	G	65-100
Ring Ouzel	*Turdus torquatus*	TUR TOR	SDM	F	A	92-138
